# GHF-ACL: A novel contrastive learning framework with multi-order graph structures for herb-disease association prediction

**DOI:** 10.1371/journal.pcbi.1014461

**Published:** 2026-06-29

**Authors:** Yunmeng Zhang, Xiuhong Wu, Qiutong Wang, Lin Shi, Meiling Liu, Guohua Wang

**Affiliations:** 1 College of Computer Science and Artificial Intelligence, Northeast Forestry University, Harbin, Heilongjiang, China; 2 College of Pharmacy, Heilongjiang University of Chinese Medicine, Harbin, Heilongjiang, China; 3 School of Computer Science and Technology, Harbin Institute of Technology, Harbin, Heilongjiang, China; Xinjiang Technical Institute of Physics and Chemistry, CHINA

## Abstract

Predicting Herb–Disease Associations (HDA) is pivotal for modernizing Traditional Chinese Medicine (TCM); however, this is impeded by data heterogeneity and the complex, multi-component mechanisms of herbal medicines. Existing drug–disease prediction models often struggle to capture high-order structural patterns and resolve semantic inconsistencies intrinsic to herbs. To overcome these limitations, we present HData, a standardized benchmark dataset that integrates herbal medicinal properties, chemical compositions, and disease associations. We further propose GHF-ACL, a novel multi-order graph contrastive learning framework designed for HDA prediction. Specifically, GHF-ACL explicitly models low-order functional similarities via a herb–disease similarity graph while capturing high-order component interactions through a herb–chemical hypergraph. Furthermore, an adaptive gating-guided structural interaction module aligns heterogeneous graph representations into a unified latent space, and hierarchical contrastive learning enforces consistency across structural views. Extensive experiments on five datasets demonstrate that GHF-ACL achieves superior or competitive performance over six state-of-the-art models across most metrics, with significant improvements over the best-performing baseline model in AUPR (+4.8% on LRSSL, + 3.81% on Cdata), F1 score, and Recall. These results underscore the model’s superior capability in detecting true positive associations within imbalanced biomedical data. By synergizing multi-view graph modeling, semantic fusion, and contrastive regularization, this work establishes a unified framework for HDA prediction, offering valuable insights for computational TCM and data-driven drug discovery.

## Author summary

Traditional Chinese Medicine has been used for centuries, but understanding how it works at a scientific level is still difficult. One reason is that herbs are naturally complex. Instead of relying on a single active substance, most herbs contain many components that work together to influence the body. This makes them hard to study using modern computer tools that are designed for simpler drugs. In our work, we aim to make this complexity easier to understand using artificial intelligence. We first collected and organized scattered information about herbs and diseases into a single, standardized data resource. We then built a computer model that looks not only at similarities between herbs and diseases, but also at how different components within an herb may work together. When we tested our approach, it was more successful than existing methods at identifying meaningful connections between herbs and diseases. As an example, our model highlighted a possible role for the herb Niuxi in treating skin cancers, which aligns with recent biological findings. Overall, we hope our study provides a clearer way to study traditional herbal medicine and helps connect ancient medical knowledge with modern, data-driven drug discovery.

## 1 Introduction

Traditional Chinese Medicine (TCM), founded upon the holistic theories of Yin-Yang and the Five Elements, constitutes a sophisticated academic system. Over thousands of years, its clinical efficacy has been empirically validated, resulting in a rich accumulation of herb-disease remedies [1].

In the context of modern medical theory, Chinese herbal medicines are characterized by synergistic multi-component and multi-target mechanisms. This inherent complexity renders the conventional reductionist approach—typical of Western medicine, which isolates single-molecule actions—insufficient for verifying the full spectrum of active ingredients in herbs. Furthermore, the experiential nature of TCM clinical practice often hinders rapid discovery in high-throughput laboratory settings, resulting in a delayed response to novel diseases [2,3]. Consequently, the concept of drug repositioning has been introduced into the TCM domain, formalized as the Herb-Disease Association (HDA) prediction task. This paradigm aims to elucidate the latent therapeutic potential of existing herbs and extend their utility beyond established indications, holding significant biological and medical implications [4,5].

In recent years, researchers have extensively explored computational methodologies for drug repositioning, such as machine learning, matrix factorization, and neural networks [4,6]. For instance, GraphDTA [7], proposed by Nguyen et al., conceptualizes drug molecules as graph structures and employs graph neural networks (GNNs) to predict drug-target affinity. Wan et al. introduced NeoDTI [8], which significantly enhances drug-target embeddings by constructing a heterogeneous network of drugs, targets, and other molecular entities, leveraging GNNs to automatically learn representation embeddings. Similarly, HGDrug [9], proposed by Jin et al., incorporates drug substructures and functional motifs to construct a hypergraph, designing a multi-branch hypergraph attention network for multi-task prediction. Hyper-Mol [10], proposed by Cui et al., encodes molecular fingerprint substructures via a hypergraph model to capture higher-order associations. Specifically addressing the multi-component and multi-target nature of TCM, HTInet [11] utilized graph embedding technology to learn low-dimensional feature representations of herbs and target proteins, pioneering the prediction of potential herb-target interactions in the TCM domain. Furthermore, HGHDA [12] was the first to employ the Transformer mechanism to aggregate dual-channel association features of herbal molecules and targets, encoding the Multi-Target Multi-Component (MTMC) properties from a pharmacological perspective. However, these existing methods often lack robust mechanisms for structural interaction and representation consistency. Critically, they fail to distinguish between diverse structural properties within the graph, making it challenging to effectively capture the high-order clustering commonalities intrinsic to TCM components.

To address these limitations, we systematically organized and standardized heterogeneous data comprising herbal properties, components, and disease associations to construct **HData**, a dedicated benchmark dataset for herb-disease prediction. Furthermore, we propose a structural-semantic alignment-driven framework named **GHF-ACL**. This approach goes beyond traditional single-graph modeling by integrating a multi-view [13], multi-level heterogeneous network. Specifically, GHF-ACL explicitly models the functional similarity between herbs and diseases via graph structures while capturing the high-order clustering semantics of herbal components through hypergraphs [14,15], thereby achieving multi-scale representation. A gating-guided structural interaction module is designed to dynamically capture complementary relationships between heterogeneous structures, facilitating hierarchical semantic alignment within a shared embedding space [16]. Additionally, local and global contrastive learning mechanisms enforce consistency at both node and subgraph levels, significantly enhancing the model’s capability to capture multi-component synergistic effects and its generalization performance [17,18].

The main contributions of this paper are summarized as follows:

- **Dataset Construction**: We constructed HData, the first standardized and multi-relational benchmark for herb–disease association prediction, which integrates herbal properties, chemical compositions, and disease ontologies to resolve data heterogeneity.
- **Methodological Innovation**: We proposed GHF-ACL, a dual-view representation learning framework that jointly models pairwise herb–disease therapeutic relationships and higher-order herb–component associations. To reconcile the semantic discrepancy between these complementary structural views, we introduce cross-view contrastive alignment and adaptive node-level fusion mechanisms, enabling more robust herb representations for association prediction.
- **Performance Superiority**: Extensive experiments on five datasets demonstrate that GHF-ACL consistently outperforms baselines across AUPR, Recall, and F1 metrics, exhibiting remarkable robustness particularly in imbalanced prediction scenarios.

Through comprehensive evaluation, we validated our method on four existing datasets in addition to HData. The results demonstrate that GHF-ACL not only achieves superior prediction accuracy on herb-disease datasets but also outperforms six state-of-the-art methods on cross-domain drug-disease datasets, highlighting its versatility and effectiveness.

## 2 Materials and methods

### 2.1 Dataset

Given the absence of a standardized dataset integrating traits, meridian medicinal properties, Yin-Yang, Five Elements, and disease associations of Chinese herbal medicine, we expanded the data sources and structural dimensions by building upon HTINet and HGHDA. We systematically compiled a large body of data on the links between medicinal herbs and diseases from authoritative databases, including TCM-ID, NPACT, the Chinese TCM Database, and NMPA. We standardized herb nomenclature by adopting the names from Chinese Materia Medica as the sole authoritative source. All collected herb data were deduplicated and mapped to their corresponding standard Latin names. Based on the deduplicated dataset, we further extracted pharmacological properties—including the Five Elements, Four Qi, Five Flavors, meridian tropism, and toxicity—by referring to the Chinese Pharmacopoeia and the TCMSP database. For herbs lacking explicitly documented properties in the Pharmacopoeia, missing attributes were imputed using the average characteristics of their corresponding plant family or genus. Herbs with confidence levels below a predefined threshold were excluded to ensure overall data quality. Active compounds and molecular structure information were extracted from the TCMSP and PubChem databases to collect the chemical constituents of each herb. These compounds were then filtered based on ADME criteria, retaining only those with oral bioavailability (OB) ≥ 30% and drug-likeness (DL) ≥ 0.18. All selected compounds were standardized into SMILES format and stored alongside the herb data in a many-to-many mapping structure. We standardized all disease names in the original association data by aligning and deduplicating them using MeSH IDs, resulting in a final curated herb–disease association dataset with normalized nomenclature. The final statistics of the organized nodes and associations are presented in Table 1.

**Table 1:** Entity and relation statistics in herbal dataset.

Herb	Disease	Chemical	Herb-Disease	Herb-Chemical
321	1712	7279	65536	9845

The above statistics reflect a multi-stage curation process. A total of 19,595 molecule–herb records were initially collected from TCMSP and PubChem, of which 7,279 unique compounds were retained after ADME-based filtering, representing a retention rate of 37.1%. The herb set was further reduced from 499 candidate entries to 321 by excluding herbs with insufficient high-confidence compound records. For herb–disease associations, 124,583 raw records were collected across the four databases; after MeSH-based disease normalization, deduplication, and nomenclature standardization, 65,536 associations were retained (retention rate 52.6%). The resulting dataset density of 11.93% is substantially higher than conventional drug–disease benchmarks, which is consistent with the broad-spectrum therapeutic characteristics of herbal medicines documented across multiple independent sources.

Isolated herb or disease nodes — those with no known associations in any of the collected databases — were removed during preprocessing, as they carry no supervisory signal for the link prediction task. This is consistent with standard practice in graph-based biomedical association prediction and was applied uniformly across all methods evaluated in this work.

Given that herbal medicines contain multiple chemical constituents with structural features comparable to those of modern drugs, and recognizing that publicly available drug–disease datasets offer superior data scale and standardization for benchmarking, we extended our comparative experiments to include four standard drug–disease association datasets in addition to the Chinese herb–disease benchmark dataset.

The detailed statistics of these datasets are summarized in Table 2. Compared with existing drug–disease datasets such as Edata, LRSSL, and Cdata, the proposed Hdata dataset exhibits several distinctive characteristics. First, Hdata is specifically designed for herbal medicine, rather than mixing herbs with chemical drugs. Second, it integrates herb–disease associations from multiple sources and adopts standardized herb nomenclature to ensure data consistency. In addition, Hdata contains a larger number of associations and richer structural information, making it more suitable for graph-based learning methods. Detailed comparisons are presented in Table 3.

**Table 2:** Dataset statistics for herb/drug-disease association prediction.

Dataset	Drugs/Herbs	Diseases	Known Associations	Sparsity
Hdata	321	1712	65536	11.9253%
Edata	3039	909	16234	0.5876%
LRSSL	763	681	3051	0.5872%
Ldata	269	598	18416	11.4483%
Cdata	663	409	2532	0.9337%

**Table 3:** Feature comparison of herb/drug–disease datasets.

Feature	Hdata (Ours)	Edata	LRSSL	Ldata	Cdata
Herb-specific dataset	Yes	Yes	No	No	No
Multi-source integration	Yes	No	No	No	No
Standardized herb names	Yes	No	No	No	No
Herb attribute information	Yes	No	No	No	No

### 2.2 Multi-order graph structure modeling framework

The proposed framework consists of four key components: a graph encoder, a hypergraph encoder, a gating-based fusion module, and a contrastive learning objective. Specifically, the graph encoder learns node representations from the heterogeneous herb–disease network, capturing pairwise associations between herbs and diseases. In parallel, the hypergraph encoder models higher-order relationships among herbs by leveraging shared chemical components.

The representations generated by these two encoders are then adaptively integrated through an adaptive gating fusion mechanism, enabling the model to effectively balance structural and high-order semantic information. Finally, a contrastive learning objective is employed to enhance the robustness and discriminative power of the learned embeddings, which are subsequently utilized for herb–disease association prediction. Unlike existing frameworks that treat these components independently, GHF-ACL integrates them as mutually dependent modules, each specifically motivated by the structural and pharmacological characteristics of TCM data.

#### 2.2.1 Multi-order similarity structure construction.

**Fig 1:**
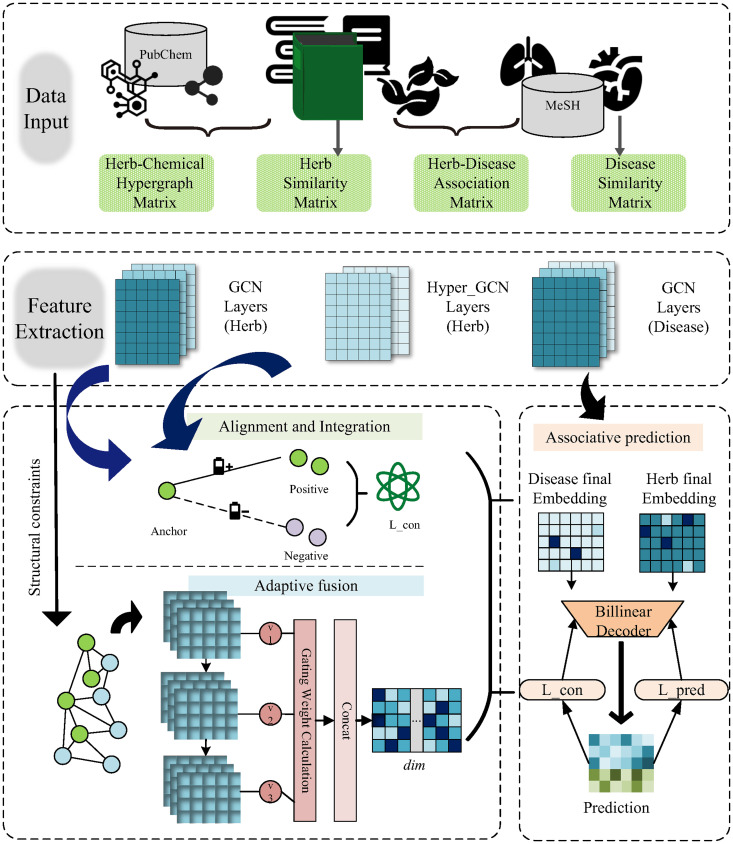
General architecture diagram. Adaptive feature fusion denotes the feature alignment module following the two convolutional layers. The green round nodes represent herbal entities, and the blue round nodes denote disease entities. (Some graphical elements in this figure were adapted from the following sources: heart organ icon, https://commons.wikimedia.org/wiki/File:Heart_organ_Pinhead_icon.svg, CC0 1.0; molecule icon, https://commons.wikimedia.org/wiki/File:Microsoft_Fluent_UI_–_ic_fluent_molecule_28_filled.svg, MIT License; three books icon, https://commons.wikimedia.org/wiki/File:Three_books_stacked_Pinhead_icon.svg, CC0 1.0; book with bookmark icon, https://commons.wikimedia.org/wiki/File:Book_with_bookmark_Pinhead_icon.svg, CC0 1.0; lungs icon, https://commons.wikimedia.org/wiki/File:Lungs_with_right_side_spots_Pinhead_icon.svg, CC0 1.0; molecule game-icon, https://commons.wikimedia.org/wiki/File:Molecule_-_Lorc_-_game-icons.svg, CC BY 3.0; leaf icon, https://commons.wikimedia.org/wiki/File:Font_Awesome_5_solid_leaf.svg, CC BY 4.0; book icon, https://bioicons.com/icons/cc-by-3.0/General_items/Servier/book.svg, CC BY 3.0.).

Firstly, regarding the construction of the low-order functional similarity structure, we calculate the similarities of herbs and diseases independently to establish a functional similarity network. Subsequently, we model the relationships between herbs and chemical components as a hypergraph, where each chemical component is represented as a hyperedge connecting all herbs that contain it. This modeling strategy is grounded in pharmacological principles of traditional Chinese medicine, which posit that the therapeutic effects of herbs are primarily mediated by their bioactive chemical constituents. Consequently, herbs sharing similar chemical components are likely to exhibit overlapping pharmacological activities, or act on similar biological targets and signaling pathways. This hypergraph modeling allows us to mine potential synergistic component groups and effectively reflect the high-order structural commonalities of herbal medicines, as illustrated in Fig 1.

According to Traditional Chinese Medicine (TCM) theory, the therapeutic efficacy of herbs is intrinsically linked to their mechanisms of action against specific diseases [1]. For instance, herbs possessing properties such as “warming Yang” or “clearing heat” exert therapeutic effects aligned with the “Cold/Heat” and “Yin/Yang” theories of TCM [3]. Consequently, to quantify the similarity between herbs, we utilize their medicinal properties as the foundational data for calculation. These properties encompass the Four Natures (cold, heat, warm, cool, and neutral), the Five Flavors (sour, bitter, sweet, pungent, salty, astringent, and bland), Meridian Tropism (heart, lung, bladder, liver, gallbladder, spleen, kidney, stomach, large intestine, small intestine, San Jiao, and pericardium), and Toxicity. We encode these attributes into binary feature vectors: if an herb possesses a specific attribute, it is marked as *‘*1’; otherwise ’0’. Finally, we employ the Jaccard index to calculate the medicinal property-based similarity between herbs [18]. The similarity between two binary feature vectors, $x_{i}$ and $x_{j}$, is computed using Eq 1.


$$\begin{array}{c} \text{Jaccard}({𝐱}_{i},{𝐱}_{j})=\frac{\sum_{k=1}^{d}x_{ik}\cdot x_{jk}}{\sum_{k=1}^{d}\max(x_{ik},x_{jk})} \end{array}$$ (1)

This encoding strategy is motivated by the categorical nature of TCM pharmacological attributes, which do not possess a natural numerical ordering. Binary encoding faithfully preserves this categorical structure, and Jaccard similarity is particularly appropriate for the resulting sparse vectors, as it measures the proportion of shared attributes relative to their union while remaining robust to the large number of shared absent features that would otherwise inflate cosine-based similarity estimates.

Following the normalization of disease names, we calculate disease similarity utilizing the MeSH system [19]. Specifically, the semantic structure of diseases in MeSH can be represented as hierarchical Directed Acyclic Graphs (DAGs) [20]. For a given disease *d*, we denote its hierarchical relationship as *DAG*(*d*)=(*N*(*d*), *E*(*d*)), where *N*(*d*) is the set of nodes comprising *d* and its ancestors, and *E*(*d*) denotes the set of directed edges connecting parent nodes to their children [21]. Based on this DAG structure, the contribution of a node *n* in *DAG*(*d*) to the semantic value of disease *d* is defined as:


$$\begin{array}{c} C_{d}(n)=\left\{ \begin{array}{cc} 1 & \text{ if }n=d\\ \Delta \cdot \max_{n^{\prime }\in \text{ children }(n)}C_{d}\left(n^{\prime }\right) & \text{ if }n\neq d \end{array}\right. \end{array}$$ (2)

When $n=d_{n}$, it indicates that the current node corresponds to the target disease itself, and its semantic contribution is defined as 1. Conversely, when $n\neq d_{n}$, the node is not the target disease, and its semantic contribution is calculated as the maximum contribution of all its child nodes multiplied by a decay factor Δ (0<Δ<1). This factor signifies that semantic information attenuates as it propagates upward from child nodes. Based on the hypothesis that diseases sharing a greater number of common ancestors in the DAG exhibit higher semantic similarity [22], we let *N*(*d*) denote the set of all ancestor nodes of disease d in the disease directed acyclic graph (DAG), including the disease itself. The semantic value of disease d is defined as follows:


$$\begin{array}{c} DV(d)=\sum_{n\in N(d)}C_{d}(n) \end{array}$$ (3)

Furthermore, *N*(*di*) and *N*(*dj*) represent the sets of ancestor nodes of diseases di and dj, respectively, in the disease DAG. we calculate the pairwise semantic similarity between diseases $d_{i}$ and $d_{j}$ using Eq 3 to construct the disease similarity matrix [23].


$$\begin{array}{c} S_{ij}^{d}=\frac{1}{DV\left(d_{i}\right)+DV\left(d_{j}\right)}\cdot \sum_{n\in 𝒩\left(d_{i}\right)\cap 𝒲\left(d_{j}\right)}\left[C_{d_{i}}(n)+C_{d_{j}}(n)\right] \end{array}$$ (4)

We construct a heterogeneous network by integrating herb–disease associations, herb–herb similarities, and disease–disease similarities [24]. Initially, the herb–disease association is represented as a binary matrix $A\in \{0,1{\}}^{N\times M}$, where *N* and *M* denote the number of herbs and diseases, respectively. The entry $A_{ij}$ equals 1 if herb $h_{i}$ is associated with disease $d_{j}$, and 0 otherwise. Furthermore, we denote the herb similarity matrix as $S_{h}\in {\mathbb{R}}^{N\times N}$ and the disease similarity matrix as $S_{d}\in {\mathbb{R}}^{M\times M}$. By normalizing and combining these similarity matrices, we obtain the unified adjacency matrix *A*.


$$\begin{array}{c} {𝒜}_{H}=\left(\begin{array}{cc} D_{r}^{-\frac{1}{2}}S^{r}D_{r}^{-\frac{1}{2}} & A\\ A^{⊤} & {\tilde{D}}_{d}^{-\frac{1}{2}}S^{d}{\tilde{D}}_{d}^{-\frac{1}{2}} \end{array}\right)\in {\mathbb{R}}^{\left(N+M)\times (N+M\right)} \end{array}$$ (5)

We conceptualize each chemical component as a hyperedge connecting all herb nodes containing that specific constituent [5]. The herb-component incidence matrix is denoted as *H*, where rows represent herbs and columns represent chemical components; specifically, $H_{ij}=1$ indicates that herb *i* contains component *j*. The degree *d* (i.e., the number of herbs associated with a component) is calculated for each hyperedge. Subsequently, the weight of each hyperedge is normalized using the inverse degree matrix *W* = diag(1/*d*) [24]. This weighting scheme penalizes the contribution of large hyperedges (common components associated with many herbs) while amplifying the significance of smaller hyperedges (unique components) [25]. Finally, based on the hypergraph structure, a weighted pairwise adjacency matrix is constructed through the following matrix operation:


$$\begin{array}{c} A_{\text{hyper}}=HWH^{T}, \end{array}$$

**Fig 2:**
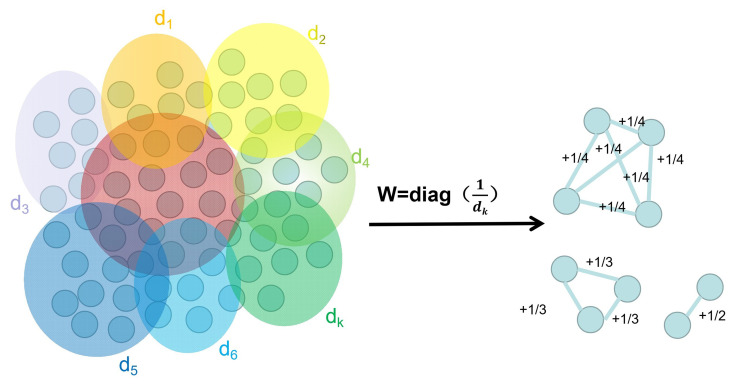
Clique expansion with uniform edge weighting. (This figure was created by the authors using Microsoft PowerPoint.).

Here, $A_{hyper}$ denotes the clique expansion of the hypergraph, which provides an intuitive pairwise representation of shared components among herbs. Acting as a weighted adjacency matrix, $A_{hyper}$ does not directly serve as an operator for hypergraph convolution. Instead, it transforms the higher-order hypergraph structure into a weighted first-order graph, thereby enabling the application of standard graph convolutional propagation rules. Consequently, each element *A*_hyper_[*i*,*j*] represents the cumulative weight between the two herbs, aggregated over all shared chemical components [26]. As illustrated in Fig 2, $d_{k}$ denotes the number of herb nodes connected by the *k*-th chemical component (hyperedge), where the weight is incremented by $\frac{1}{d_{k}}$ whenever two herbs share the chemical component *k*.

In this formulation, the weight of each hyperedge is evenly distributed among all pairs of herb nodes that share the corresponding chemical component, including self-connections. This ensures that components associated with multiple herbs do not disproportionately dominate the resulting adjacency structure.

#### 2.2.2 Multi-order embedding representation learning.

**Fig 3:**
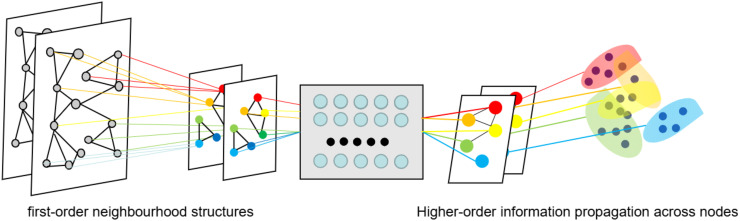
Multi-order embedding function diagram. (This figure was created by the authors using Microsoft PowerPoint.).

The graph encoder takes the unified heterogeneous adjacency matrix $A_{H}$ as input and employs graph convolution to learn node representations for both herbs and diseases. In parallel, the hypergraph encoder operates on the herb–component hypergraph defined by the incidence matrix *H*, capturing higher-order relationships among herbs that share common chemical components. We construct embedding modules on the functional graph *G* and the constituent hypergraph *H*, respectively, to realize the collaborative learning of semantics from both first-order and higher-order structures, as illustrated in Fig 3.

We employ Graph Convolutional Networks (GCN) for feature extraction of herbs and diseases within the functional graph *G*. The encoding of higher-order structural features is achieved by stacking multi-layer graph convolution operations, which allows nodes to iteratively aggregate semantic information from increasingly distant neighbors within the graph structure [27]. The core principle is to locally smooth node features via neighborhood relations, thereby promoting similarity among adjacent nodes in the representation space. This process effectively captures the latent clustering structures and semantic associations inherent in the graph. In each GCN layer, the node embeddings are updated through the weighted aggregation of neighboring node representations, facilitating feature learning that propagates from immediate first-order neighbors to multi-order neighbors.

Its standard inter-layer propagation rule can be formalized as follows:


$$\begin{array}{c} X^{\left(l+1\right)}=\sigma \left(A_{H}X^{\left(l\right)}W^{\left(l\right)}\right) \end{array}$$ (6)

where *X*^(*l*)^ denotes the node embedding in the *l* -th layer, $A_{H}$ represents the normalized graph adjacency matrix, *W*^(*l*)^ indicates the trainable weight matrix in layer *l*, and σ(·) is the nonlinear activation function. The initia*l* node embedding *X*^(0)^ can be initialized as an identity matrix or via random initialization. Consequently, the first layer of the GCN encoder corresponds to the case where *l* = 0 in the inter-layer propagation rule. Subsequent layers adhere to the aforementioned propagation ru*l*e, ultimately generating the sequence of layer-wise node embeddings:


$$\begin{array}{c} X^{\left(1\right)},\;X^{\left(2\right)},\;X^{\left(3\right)},\;\ldots ,\;X^{\left(l\right)}. \end{array}$$

Higher-order representations of herbs within the component view are extracted using HyperGCN on the component hypergraph *H*, implementation of spectral convolution using a normalised propagation operator based on the hypergraph Laplacian. A key advantage of this module lies in its capability to capture non-pairwise higher-order relationships among multiple herbs connected by shared constituents. This, in turn, reveals the latent modular distribution of specific herbs within the constituent functional space. We define the herbal node degree matrix


$$\begin{array}{c} D_{V}=diag\left(d_{V_{1}},\cdots ,d_{V_{n}}\right) \end{array}$$

where $d_{V_{i}}=\sum_{j}w_{j}H_{ij}$ represents the weighted quantity of the components contained in the herbal medicine $v_{i}$. The super-edge matrix is defined as


$$\begin{array}{c} D_{E}=diag\left(\delta_{e_{1}},\cdots ,\delta_{e_{n}}\right) \end{array}$$

where $\delta_{e_{j}}=\sum_{i}H_{ij}$ denotes the number of herbs containing the component $e_{j}$. Since the hyperedge weight matrix W takes the form of the reciprocals of the hyperedge degrees, and in conjunction with the normalisation $D_{E}^{-1}$ in the propagation operator, the effective weight of hyperedge $e_{j}$ is:


$$\begin{array}{c} \frac{w_{j}}{\delta_{e_{j}}}=\frac{1}{\delta_{e_{j}}^{2}} \end{array}$$

This mechanism naturally mitigates the excessive influence of commonly shared components on node representations during the aggregation process, thereby enabling nodes with rare shared components to acquire relatively stronger semantic representations.

The normalised hypergraph Laplace operator *L* is defined as


$$\begin{array}{c} L=I-D_{v}^{-\frac{1}{2}}HWD_{e}^{-1}H^{⊤}D_{v}^{-\frac{1}{2}} \end{array}$$ (7)

where *W* is the hyperedge weight matrix.We perform the eigendecomposition of the hypergraph Laplacian *L* as


$$\begin{array}{c} L=U\Lambda U^{⊤} \end{array}$$

where the eigenvector matrix *U* forms the hypergraph Fourier basis, and the eigenvalues $\lambda_{k}\in [0,2]$ correspond to frequency components on the hypergraph. The Fourier transform of a hypergraph signal *f* is then defined as


$$\begin{array}{c} \hat{f}=U^{⊤}f \end{array}$$

We adopt a first-order polynomial approximation filter


$$\begin{array}{c} g_{\theta }(\lambda_{k})=1-\lambda_{k} \end{array}$$

Since this response function approaches 1 when $\lambda_{k}\approx 0$ and approaches 0 when $\lambda_{k}\approx 1$, it exhibits a clear low-pass filtering characteristic. The corresponding spatial-domain propagation operator is given by:


$$\begin{array}{c} \Theta =I-L=D_{V}^{-\frac{1}{2}}HWD_{E}^{-1}H^{T}D_{V}^{-\frac{1}{2}} \end{array}$$ (8)

It should be emphasized that the low-pass characteristic originates from the eigenvalue decay mechanism of the filter response function


$$\begin{array}{c} g(\lambda )=1-\lambda \end{array}$$

rather than from the Laplacian operator **L** itself. The forward propagation of the *l*-th layer in HyperGCN consists of the fo*l*lowing three steps:

1. Hyperedge aggregation: Each hyperedge $e_{j}$ aggregates the embeddings of its member herb nodes by normalized averaging:
  $$\begin{array}{c} {𝐌}^{\left(l\right)}=D_{E}^{-1}H^{⊤}{𝐙}^{\left(l\right)}\in {\mathbb{R}}^{m\times d_{l}} \end{array}$$ (9)
2. Weighted reverse distribution: Each herb node receives the aggregated information from its incident hyperedges:
  $$\begin{array}{c} {\tilde{𝐙}}^{\left(l\right)}=HW{𝐌}^{\left(l\right)}=HWD_{E}^{-1}H^{⊤}{𝐙}^{\left(l\right)}\in {\mathbb{R}}^{n\times d_{l}} \end{array}$$ (10)
  where *W* is a diagonal matrix of hyperedge weights.
3. Symmetric normalization and linear transformation: The node embeddings are symmetrically normalized by node degrees and passed through a learnable linear transformation:
  $$\begin{array}{c} {𝐙}^{\left(l+1\right)}=\sigma \left(D_{V}^{-1/2}HWD_{E}^{-1}H^{⊤}D_{V}^{-1/2}{𝐙}^{\left(l\right)}\Theta^{\left(l\right)}\right),\;{𝐙}^{\left(0\right)}={𝐗}_{h} \end{array}$$ (11)
  where ${𝐗}_{h}\in {\mathbb{R}}^{N\times d}$ is the initial feature matrix of herb nodes, $\Theta^{\left(l\right)}\in {\mathbb{R}}^{d_{l}\times d_{l+1}}$ is the learnable weight matrix at layer *l*, and σ is a non-linear activation function.

After stacking *L* layers of convolution, the embedding of herb node $v_{i}$ at layer *l* can be expanded as:


$$\begin{array}{c} z_{i}^{\left(l+1\right)}=\sigma \left(\frac{1}{d_{v_{i}}^{\frac{1}{2}}}\sum_{j:v_{i}\in e_{j}}\frac{1}{\delta_{e_{j}}^{2}}\sum_{k:v_{k}\in e_{j}}\frac{z_{k}^{\left(l\right)}}{d_{v_{k}}^{\frac{1}{2}}}\cdot \Theta^{\left(l\right)}\right) \end{array}$$ (12)

It can be observed that each herb node not only directly aggregates information from its associated components but also indirectly acquires semantic information from other herbs through shared component hyperedges. Specifically, node $v_{i}$ aggregates the embeddings of all herbs $v_{k}$ within each hyperedge $e_{j}$ it belongs to, with a weighting factor of $1/\delta_{e_{j}}^{2}$, which effectively suppresses the dominating effect of large hyperedges. This mechanism enables multi-hop, high-order co-occurrence semantic propagation in the component space. The final output ${𝐙}^{\left(L\right)}$ serves as the structural representation of each herb in the component modality, providing high-order topological features for subsequent multi-modal fusion.

#### 2.2.3 Multi-order structural alignment with contrastive learning.

**Fig 4:**
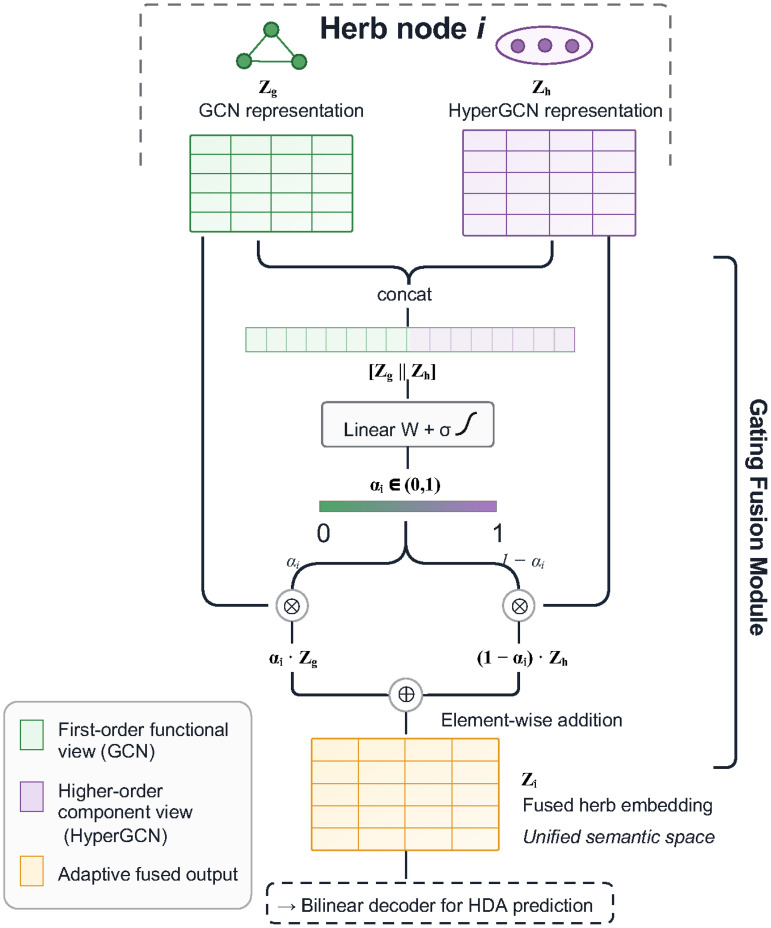
Multi-order structural alignment based on the gating fusion mechanism.

Due to the fundamental discrepancies between GCN and HyperGCN in relational modeling, the node representations learned by these two paradigms are typically embedded in heterogeneous latent spaces. Consequently, direct fusion may result in semantic inconsistency and potential information interference. To mitigate this issue, we propose a contrastive learning-guided gating-based fusion mechanism, based on the scalar gating mechanism, which facilitates the adaptive integration of multi-order structural information while explicitly aligning representations derived from different structural views, as illustrated in Fig 4. Notably, as the hypergraph is constructed solely within the herb node space, the multi-structure representation alignment is conducted exclusively on herb nodes.

Specifically, we first employ the gating mechanism to fuse the node representations derived from the two structural views. For each herb node *i*, let $z_{i}^{\left(g\right)}$ and $z_{i}^{\left(h\right)}$ denote its representations in the graph and hypergraph, respectively. The fusion weights are computed as follows, yielding the final representation:


$$\begin{array}{c} \alpha_{i}=\sigma (W[z_{i}^{\left(g\right)}\;∥\;z_{i}^{\left(h\right)}]) \end{array}$$

$$\begin{array}{c} z_{i}=\alpha_{i}z_{i}^{g}+\left(1-\alpha_{i}\right)z_{i}^{h} \end{array}$$

Here, $\alpha_{i}$ denotes the adaptive weight of a node across different structural representations, $W\in R^{1\times 2d}$, input concatenated vectors $[z_{i}^{\left(g\right)}\;∥\;z_{i}^{\left(h\right)}]\in {\mathbb{R}}^{2d}$ ensures dimensional consistency in gating weight calculations. To control model complexity and prevent overfitting, we adopt a lightweight scalar gating mechanism to weight the fusion of representations from different structures, thereby enhancing training stability while preserving expressive capacity. However, due to the semantic shift between the two structural spaces, relying solely on the gating mechanism for fusion may amplify the noise introduced by misaligned representations. To address this, we further introduce contrastive learning as an alignment constraint, explicitly aligning the multi-order representations prior to fusion.

Specifically, for each herb node, its representation $z_{i}^{\left(g\right)}$ in the GCN is treated as an anchor, while the corresponding representation $z_{i}^{\left(h\right)}$ in the HyperGCN serves as a positive sample. Negative samples are constructed from other nodes across the two structural views to enforce cross-view contrastive learning. We adopt a 1:n negative sampling strategy. Specifically, for each positive pair $\left(z_{i}^{\left(g\right)},z_{i}^{\left(h\right)}\right)$, we randomly sample n hypergraph representations $z_{k}^{\left(h\right)}$ of different nodes within the current mini-batch as negative samples, thereby constructing the following cross-view contrastive loss:


$$\begin{array}{c} L_{con}=-\sum_{i}\log\frac{\exp(sim(z_{i}^{\left(g\right)},z_{i}^{\left(h\right)})/\tau )}{\exp(sim(z_{i}^{\left(g\right)},z_{i}^{\left(h\right)})/\tau )+\sum_{k\neq i}\exp(sim(z_{i}^{\left(g\right)},z_{k}^{\left(h\right)})/\tau )} \end{array}$$ (13)

This loss encourages the representations of the same node across different structures to be close in the embedding space, while pushing apart the representations of different nodes, thereby achieving consistent alignment of multi-order structural semantic spaces. It is worth noting that negative samples are constructed only across views, without involving intra-view contrast, allowing the model to focus specifically on the cross-structure alignment task. Building on this, contrastive learning not only serves as an auxiliary supervisory signal but also acts as a regularization constraint for the gating-based fusion process. This encourages the gating mechanism to perform information selection within a unified semantic space, mitigating bias toward misaligned or noisy features and enabling more stable and reliable feature fusion.

Furthermore, at the global level, we formulate herb–disease association prediction as a supervised learning task and employ a weighted binary cross-entropy loss to alleviate class imbalance. This loss is defined as the supervised prediction loss:


$$\begin{array}{c} L_{pred}=-\sum_{(i,j)\in Y^{+}}W^{+}\log{\hat{y}}_{ij}-\sum_{(i,j)\in Y^{-}}W^{-}\log(1-{\hat{y}}_{ij}) \end{array}$$ (14)

Here, ${\hat{y}}_{ij}$ denotes the predicted probability of an association between herb $h_{i}$ and disease $d_{j}$.*W*^+^ and $W^{-}$ represent the weights for positive and negative samples, respectively, designed to mitigate class imbalance. By incorporating this weighting mechanism, we increase the contribution of positive samples in the loss function, thereby enhancing the model’s discriminative capability for the minority class. In practice, the weights are set according to the ratio of positive to negative samples, as follows:


$$\begin{array}{c} W^{+}\propto \frac{1}{|Y^{+}|},W^{-}\propto \frac{1}{|Y^{-}|} \end{array}$$

This ensures that the different categories have a relatively balanced influence during the training process. Finally, the overall optimization objective of the model is defined as:


$$\begin{array}{c} L=L_{pred}+\lambda L_{con} \end{array}$$

Here, λ is used to balance the relative importance of representation alignment and the association prediction tasks. It is important to note that $L_{pred}$ directly optimizes the performance of association prediction, while $L_{con}$ enforces consistency between representations from different structures; the two terms work synergistically within a unified framework. To further enhance the model’s generalization ability, node dropout is applied in certain graph convolutional layers, and standard dropout is employed during representation learning to prevent overfitting.

#### 2.2.4 Prediction and joint optimization objective.

Finally, we employ a bilinear scoring mechanism to compute the herb-disease association scores. Specifically, let ${𝐳}_{i}^{\left(h\right)}\in {\mathbb{R}}^{d}$ and ${𝐳}_{j}^{\left(d\right)}\in {\mathbb{R}}^{d}$ denote the corresponding embedding vectors for herb node $h_{i}$ and disease node $d_{j}$, respectively. We model the interactions between these nodes via a learnable bilinear weight matrix $W\in {\mathbb{R}}^{d\times d}$, and compute the association scores using the following equation:


$$\begin{array}{c} {\hat{y}}_{ij}=\sigma (Z_{i}^{\left(h\right)}WZ_{j}^{\left(d\right)}) \end{array}$$ (15)

Here, σ(·) denotes the sigmoid activation function, which maps the output to the [0,1] range, representing the probability of an association between herb $h_{i}$ and disease $d_{j}$.

Compared to simple vector inner product or concatenation methods, the bilinear decoder introduces a learnable weight matrix *W*, enabling explicit modeling of interactions across different latent dimensions and capturing more complex feature crossover patterns. Essentially, the decoder implements a second-order feature interaction function, representing a weighted combination of latent semantic dimensions. Coupled with the previously described gating-based fusion and contrastive alignment mechanisms, the herb node representations $z_{i}^{h}$ have already integrated multi-order structural information within a unified semantic space. This provides a more consistent and discriminative foundation for the bilinear decoder to model herb–disease associations.

In other words, the graph encoder captures the heterogeneous network topology, the hypergraph encoder models higher-order component-sharing relationships among herbs, the gating module integrates multi-order graph representations, and the contrastive learning objective further enhances embedding quality. During training, the parameter matrix *W* is dynamically updated via backpropagation to fit known association patterns, thereby enhancing the model’s predictive capability and generalization performance for potential unknown associations.

### 2.3 Data flow

For clarity, we describe the data flow of the proposed model in a dimension-consistent manner. The model takes as input a standard adjacency matrix of size (2033,2033) and a hypergraph-based feature matrix of size (2033,7279). The standard feature matrix is processed by the GCN branch and projected into a latent space of size (2033,128) through multiple graph convolution layers, followed by layer-wise aggregation to obtain the final GCN embedding. In parallel, the hypergraph feature matrix is transformed into the same latent space (2033,128) and further refined via hypergraph convolution, resulting in the hypergraph embedding of size (2033,128). The two embeddings are then used to compute a similarity matrix of size (2033,2033) for contrastive learning. After cross-view alignment, they are fused into a unified representation of size (2033,128). The fused representation is subsequently divided into herb embeddings of size (321,128) and disease embeddings of size (1712,128), which are used to generate a prediction matrix of size (321,1712).

## 3 Experimental results

### 3.1 Experimental setup

To evaluate the performance of GHF-ACL, experiments were conducted using a self-constructed herb–disease association dataset, which integrates herbal medicinal properties and incorporates molecular hypergraphs based on herb–chemical mappings. We employed a five-fold cross-validation strategy to ensure robustness and assess generalization capabilities. The dataset was randomly partitioned into five equal subsets; in each iteration, one subset served as the test set while the remaining four constituted the training set. This procedure was repeated five times, and the results were averaged to mitigate randomness and enhance statistical reliability.

Additionally, the Receiver Operating Characteristic (ROC) curve and the Precision-Recall (PR) curve were utilized to evaluate the model’s binary classification performance across varying thresholds. The ROC curve depicts the True Positive Rate (TPR) against the False Positive Rate (FPR), while the PR curve illustrates Precision against Recall. These metrics are calculated as follows:


$$\begin{array}{c} TPR=Recall=\frac{TP}{P}=\frac{TP}{TP+FN} \end{array}$$ (16)

$$\begin{array}{c} FPR=\frac{FP}{N}=\frac{FP}{FP+TN} \end{array}$$ (17)

$$\begin{array}{c} Precision=\frac{TP}{TP+FP} \end{array}$$ (18)

where TP, FP, and TN denote true positives, false positives, and true negatives, respectively.

For the supervised prediction task, positive samples consist of known herb–disease associations in the training set. Negative samples for supervised learning are randomly drawn from unobserved herb–disease pairs at a ratio of 1:10 (positive to negative). For the cross-view contrastive learning objective, we adopt an InfoNCE-based strategy in which, for each anchor node, the representations of other herb nodes within the same mini-batch are treated as negative samples. Since the mini-batch composition varies across training iterations, the negative set is dynamically updated, which helps mitigate the bias introduced by fixed negative sampling.

To ensure clarity and consistency, we explicitly distinguish three types of negative samples used in different stages of the framework:

1. Negative samples for supervised link prediction: sampled at 1:10 ratio during training;
2. Negative samples for contrastive learning: dynamically constructed within mini-batches for cross-view alignment, independent of association labels;
3. Negative samples for evaluation: all unobserved herb–disease pairs in the test set are treated as negatives without any sampling, to reflect real-world imbalance.

This unified evaluation protocol is applied to both standard five-fold cross-validation (edge-level split) and node-level (cold-start) split to ensure fairness.

Given the highly imbalanced nature of the herb–disease association prediction task, AUPR is adopted as the primary evaluation metric, as it emphasizes the model’s ability to correctly identify positive associations. In contrast, accuracy can be misleading in such scenarios due to the predominance of negative samples. In addition, we also report other metrics, including AUC, F1-score, precision, and recall, to provide a complementary assessment of the model’s performance from multiple perspectives.

### 3.2 Experimental and comparative results

**Fig 5:**
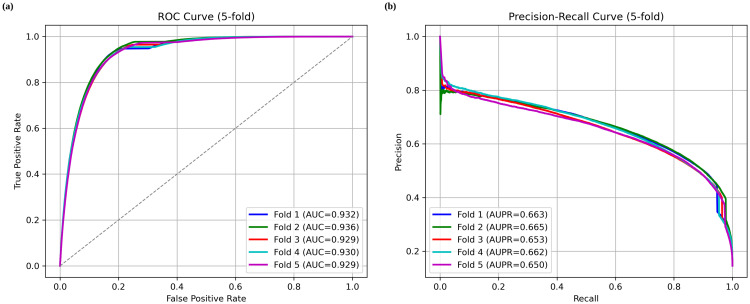
Performance comparison of the GHF-ACL model on the Hdata dataset under 5-fold cross-validation. (a) ROC curves of the GHF-ACL model under 5-fold cross-validation on the Hdata dataset; (b) PR curves of the GHF-ACL model under 5-fold cross-validation on the Hdata dataset.

The performance of GHF-ACL was evaluated via five-fold cross-validation on the Hdata dataset. The specific AUROC and AUPR values for each fold are presented in Table 4, while the corresponding ROC and PR curves are illustrated in Fig 5(a) and 5(b), respectively. The model achieved an average AUROC of 0.9312 and an average AUPR of 0.6586, indicating robust predictive performance on the HAD task. Furthermore, the high consistency observed across the five AUROC and AUPR values demonstrates the model’s stability. This conclusion is further corroborated by the close alignment of the five ROC curves depicted in Fig 5(a).

**Table 4:** Five-fold cross-validation results of the GHF-ACL model on Hdata.

Fold	AUROC	AUPR
Fold 1	0.932	0.663
Fold 2	0.936	0.665
Fold 3	0.929	0.653
Fold 4	0.930	0.662
Fold 5	0.929	0.650
**Mean**	**0.9312**	**0.6586**

We compared our proposed model against six classical association prediction methods and five models specifically tailored for the HDA task. A brief overview of these baseline models is provided below:

- LAGCN [28]: Adaptively fuses feature representations across different layers of a graph convolutional network (GCN) via a layer-wise attention mechanism to address the over-smoothing problem inherent in traditional GCNs.
- HDGAT [29]: A Hierarchical Dual Graph Attention Network designed to model both the local and global semantics of drug–disease associations.
- DRWBNCF [30]: A collaborative filtering framework that combines deep residual networks with a Bayesian weighting strategy to effectively manage implicit feedback data.
- DD-HGNN+ [31]: A Dual-Domain Heterogeneous Graph Neural Network that integrates drug–disease associations with functional similarity information.
- AdaDR [32]: An adaptive deep regression model that jointly optimizes classification and regression objectives to enhance predictive robustness.
- DRAGNN [33]: A relational attention-based graph neural network that explicitly models multiple relationship types within drug–disease associations.
- HGHDA [12]: A Dual-Channel Hypergraph Convolutional Neural Network that integrates information regarding herb components and targets.
- HTInet-KNN [12]: A method utilizing the node2vec algorithm to learn feature representations of herbs and proteins within the network, subsequently employing a K-Nearest Neighbor (KNN) classifier.
- HTInet-RF [12]: A method utilizing the node2vec algorithm to learn feature representations of herbs and proteins within the network, subsequently applying a Random Forest (RF) classifier.
- GeNNius [34]: A graph neural network-based method for ultra-fast inference of drug–target interactions.
- HDCTI [35]: A graph learning framework that leverages hypergraph-based representation learning to model higher-order and complex relationships among herbs, enabling the prediction of novel targets for natural compounds.

To ensure fairness and transparency in the comparison, all baseline models are implemented and evaluated under a unified experimental protocol. Specifically:

(1) Data splitting: All methods adopt the same partition of training, validation, and test sets.
(2) Input features: All models are provided with identical input data, including herb–disease associations and node features, without incorporating any additional external information.
(3) Negative sampling: A consistent random negative sampling strategy is applied across all methods to construct training instances. To better simulate real-world scenarios, the ratio of positive to negative samples is fixed at 1:10.
(4) Evaluation metrics: All methods are evaluated using the same metrics, including AUC, AUPR, and F1-score. Under the global negative sampling protocol, the task becomes significantly more challenging due to increased data sparsity. Therefore, AUPR is regarded as the primary evaluation metric, as it is more sensitive to precision in highly imbalanced settings.

The results in Tables 5 and 6 present a comparative analysis of various methods under five-fold cross-validation. GHF-ACL consistently outperforms the other models in AUPR, Recall, and F1 Score across all evaluated datasets, while AUC performance varies across datasets due to differences in class imbalance severity. Notably, GHF-ACL achieves the highest AUPR values across all three datasets. This indicates that the model possesses a robust capability for identifying positive samples, making it particularly effective for addressing the sample imbalance problem that is prevalent in association prediction tasks.

**Table 5:** Performance comparison of different models on LRSSL, Ldata and Cdata datasets.

DataSet	Model	AUPR	Accuracy	Precision	Recall	F1 Score	AUC
LRSSL	LAGCN	0.3503	0.3955	0.0091	0.6875	0.0168	0.6044
	HDGAT	0.0610	0.9976	0.0787	0.0855	0.0797	0.6394
	DRWBNCF	0.2405	0.9693	0.3114	0.3027	0.2958	0.8933
	DD-HGNN+	0.7460	0.9996	0.9082	0.7624	0.8289	0.9991
	AdaDR	0.4269	0.9947	0.5786	0.4038	0.4716	0.9023
	DRAGNN	0.5082	0.9940	0.4943	0.5654	0.5274	0.9576
	GHF-ACL	**0.7821**	0.9988	0.7247	**0.9922**	**0.8510**	0.9347
Ldata	LAGCN	0.3007	0.9616	0.2892	0.3482	0.3140	0.8730
	HDGAT	0.2665	0.9614	0.2762	0.3221	0.2964	0.8642
	DRWBNCF	0.4274	0.8460	0.3794	0.5413	0.4460	0.8269
	DD-HGNN+	0.6886	0.9820	0.6527	0.6128	0.6325	0.9780
	AdaDR	0.5381	0.8831	0.4932	0.5594	0.5228	0.8637
	DRAGNN	0.4233	0.8524	0.3922	0.5190	0.4463	0.8184
	GHF-ACL	**0.6979**	0.8140	**0.6719**	**0.8865**	0.5809	0.9273
Cdata	LAGCN	0.1254	0.9679	0.0142	0.2354	0.0269	0.8063
	HDGAT	0.0439	0.9861	0.0172	0.1122	0.0296	0.6593
	DRWBNCF	0.2803	0.9883	0.3854	0.3029	0.3336	0.8685
	DD-HGNN+	0.8954	0.9995	0.8356	0.9336	0.8819	0.9996
	AdaDR	0.6269	0.9939	0.7385	0.5375	0.6216	0.9449
	DRAGNN	0.6133	0.9934	0.6848	0.5549	0.6122	0.9562
	GHF-ACL	**0.9295**	**0.9996**	0.6355	**0.9528**	**0.9241**	0.8157

**Table 6:** Performance comparison of different models on Ethnobotany datasets.

DataSet	Model	AUPR	Precision	Recall	F1 Score	AUC
Ethnobotany	HGHDA	0.9033	0.8196	0.8618	0.8400	0.9028
	HTINet(KNN)	0.5785	0.5016	0.9956	0.6671	0.5707
	HTInet(RF)	0.7529	0.5566	0.8918	0.6852	0.7218
	GHF-ACL	**0.9324**	**0.7217**	**0.9955**	**0.9885**	**0.9296**

On the LRSSL, Ldata, and Cdata datasets, GHF-ACL achieved AUPR improvements of 4.8%, 1.3%, and 3.81% respectively over the second-best performing methods, thereby demonstrating superior discriminative capability for positive samples. Additionally, the model attained a Recall of 0.9922 and an F1 Score of 0.8510, indicating high sensitivity to true positives while maintaining a balanced performance.

To further evaluate the generalization capability of the model in realistic scenarios, we introduce a more challenging node-level splitting strategy in addition to the conventional random edge split. Under this setting, nodes appearing in the test set are entirely unseen during the training phase, posing a stricter evaluation of model robustness. Furthermore, to ensure consistent and fair evaluation under this strict node-level protocol, we use the same evaluation setting as in five-fold cross-validation: all unobserved herb–disease pairs in the test set are treated as negatives without sampling. This avoids evaluation bias and ensures comparability across all models. This design enables a more fine-grained evaluation of the model’s capability to distinguish subtle structural differences in local neighborhoods. We further benchmark our method against two state-of-the-art graph contrastive learning models, GeNNius and HDCTI, both of which are evaluated under this new protocol.

We adapted GeNNius [34] within a unified framework to ensure consistency with our method in terms of task definition and input format. Specifically, the original drug and target nodes in GeNNius were mapped to herb and disease nodes, respectively, and a bipartite graph was constructed based on known herb–disease associations. In this way, the original drug–target prediction problem is naturally transformed into a herb–disease association prediction task. To guarantee a fair comparison, GeNNius was trained and evaluated under the same data splits, negative sampling strategy, and evaluation metrics as our method.

Similarly, the original HDCTI [35] model was designed to predict interactions between natural compounds and biological targets, with its core relying on hypergraph-based representation learning to capture higher-order associations between compounds and targets. To adapt it to our task, we first performed a relation-level mapping, treating herb–disease associations as analogous to compound–target interactions in HDCTI. This allows the model to maintain its original architecture while aligning its prediction objective with the herb–disease association task. The results are summarized in Table 7. To ensure a fair and strict comparison, all baseline models are re evaluated under the same node level (cold start) split protocol.

**Table 7:** Performance comparison of graph-based models on Hdata under the node-level split (cold-start) setting.

Dataset	Model	AUPR	Precision	Recall	F1 Score	AUC
Hdata	GHF-ACL(Ours)	**0.8912**	**0.8281**	**0.8880**	**0.8572**	**0.9247**
	GeNNius	0.5983	0.5751	0.7940	0.6650	0.7054
	HDCTI	0.7292	0.7034	0.8040	0.7435	0.8103
	LAGCN	0.4377	0.3820	0.7652	0.5102	0.6235
	HDGAT	0.3584	0.3129	0.7281	0.4362	0.5898
	DD-HGNN+	0.7423	0.7215	0.8127	0.7646	0.8581
	AdaDR	0.3689	0.3452	0.6893	0.4596	0.6078
	DRAGNN	0.5588	0.5230	0.7617	0.6203	0.7289

While GHF-ACL did not consistently yield the highest Accuracy or Precision, it exhibited stable overall performance. Notably, it secured particularly strong results in AUPR and F1 Score, which are considered more reliable indicators of model efficacy in imbalanced scenarios. The discrepancy between AUC and AUPR indicates that, although some competing methods may achieve better overall ranking performance, GHF-ACL is more effective at prioritizing true positive associations, which is crucial in practical applications. Additionally, the slightly lower precision observed on the Cdata dataset can be attributed to its extreme sparsity. In such cases, GHF-ACL tends to emphasize higher recall to ensure broader coverage of potential associations.

Consequently, this architecture is particularly well-suited for TCM-based prediction tasks, where heterogeneous structures and sparse supervision present significant modeling challenges.

### 3.3 Visualisation results

To further evaluate whether the node representations learned by the model exhibit strong structural and semantic consistency, we perform a quantitative assessment from two perspectives: clustering quality and biological semantics.

**Fig 6:**
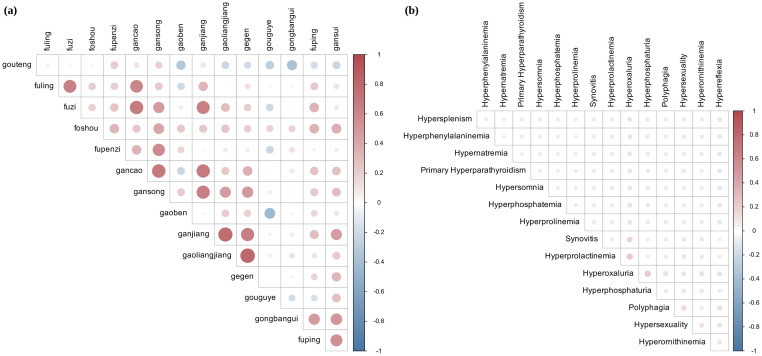
Similarity heatmaps of herbs and diseases. The color shades indicate the semantic similarity between nodes, where darker colors represent higher similarity. (a) Fifteen herbs similarity heatmap. (b) Fifteen diseases similarity heatmap.

Fig 6(a) presents the similarity heatmap derived from 15 representative herbs, illustrating the pairwise similarities among them. The chromatic intensity in the heatmap corresponds to the degree of similarity, with darker hues indicating higher similarity. Observations reveal that herbs sharing similar therapeutic effects or pharmacological classifications (e.g., Ganjiang and Gaoliangjiang) exhibit pronounced high-similarity regions within the heatmap. This indicates that the model effectively captures pharmacological affinities among herbs within the embedding space. These results suggest that the model learns robust herb representations by integrating both network structure and semantic features, thereby establishing a solid foundation for subsequent herb–disease association prediction.

Similarly, a selection of common disease nodes was utilized to compute pairwise similarities, with the resulting heatmap displayed in Fig 6(b). The analysis demonstrates that diseases associated with related pathological mechanisms exhibit distinct clustering patterns within the embedding space. This indicates that the model not only achieves effective structural representation learning for herbs but also captures semantic consistency at the disease level, which further supports the interpretability of the prediction results.

**Fig 7:**
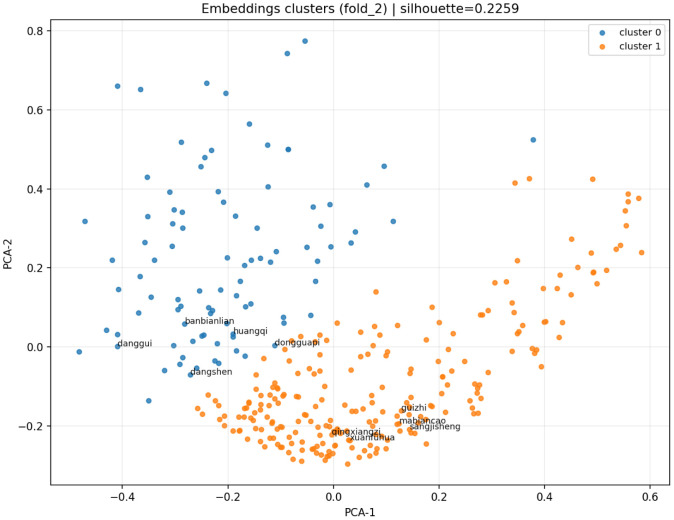
Figure PCA visualization of the learned herb embeddings for fold_2, with a silhouette score of 0.2259. The embeddings are clustered into two distinct groups, with representative herbs labeled near the cluster centroids. The visualization demonstrates that the model effectively groups herbs with similar pharmacological properties, validating the semantic consistency of the learned representations.

To improve interpretability, we employed PCA to visualize the clustering results. Unlike t-SNE, PCA preserves the global structure, rendering the observed clustering separation outcomes more reliable. As shown in the Fig 7, We further labeled the representative herbs closest to the cluster centroids and observed that each cluster integrates multiple medicinal herbs with distinct pharmacological properties. To verify the semantic consistency of the learned embeddings, we manually examined the representative Chinese medicinal herbs nearest to the cluster centroids. For instance, one cluster contains herbs such as Codonopsis pilosula (Dangshen), Astragalus membranaceus (Huangqi), and Angelica sinensis (Danggui), which are commonly associated with tonic and immunomodulatory functions. In contrast, the other cluster includes herbs like Cinnamomum cassia (Guizhi) and Taxillus chinensis (Sangjisheng), which are primarily used for dispelling wind-dampness and treating exterior syndromes. This observation demonstrates that the learned embeddings can group Chinese medicinal herbs with similar pharmacological properties, further validating that the model is capable of capturing meaningful biological semantics. Additionally, we calculated the average intra-cluster and inter-cluster distances in the embedding space.Intra-class distance:


$$\begin{array}{c} d_{intra}=\frac{1}{|C{|}^{2}}\sum_{i,j\in C}∥z_{i}-z_{j}∥ \end{array}$$ (19)

Distance between classes:


$$\begin{array}{c} d_{inter}=\frac{1}{|C_{1}||C_{2}|}\sum_{i\in C_{1},j\in C_{2}}∥z_{i}-z_{j}∥ \end{array}$$ (20)

We calculate the average distances within and between classes in the embedding space in Table 8. The experimental results show that:


$$\begin{array}{c} d_{intra}<d_{inter} \end{array}$$

**Table 8:** Intra- and inter-distance for each category.

Category	Intra-distance	Inter-distance
1	0.571	1.175
2	0.413	1.123

The clustering metrics indicate that, although the separation between clusters is not particularly pronounced, the learned embedding vectors still exhibit a certain degree of structural organisation.

### 3.4 Ablation experiments

**Fig 8:**
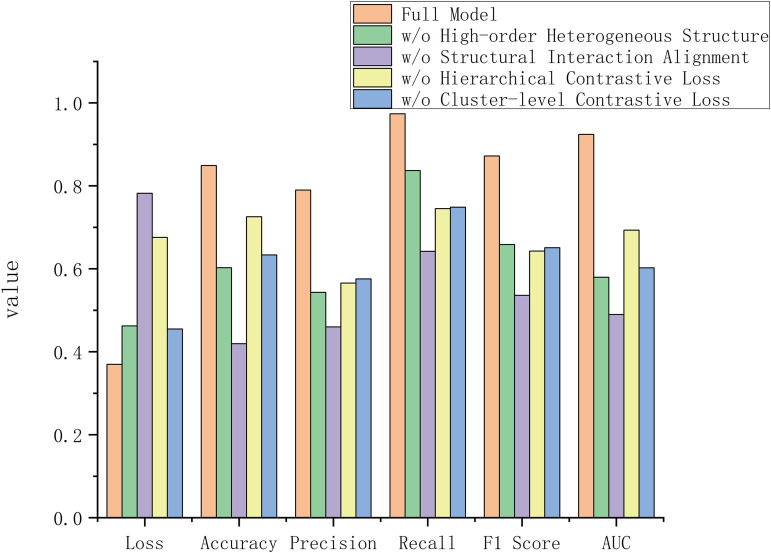
Comparative results of ablation experiments in Hdata.

To rigorously evaluate the impact of each key module on model performance, we designed a comprehensive set of ablation experiments. Utilizing the herb–disease dataset (results shown in Fig 8) and the open standard dataset LRSSL (results shown in Table 9), we compared the proposed model against four specific variants:

- **w/o High-order Heterogeneous Structure:** This variant removes the hypergraph constructed from herb–chemical compositions, retaining only the first-order herb–disease graph structure.
- **w/o Structural Interaction Alignment:** This variant replaces the adaptive gating fusion mechanism with simple concatenation.
- **w/o Hierarchical Contrastive Loss:** This variant eliminates the local bi-level contrastive learning loss, retaining only the cluster-level contrast as the optimization objective.
- **w/o Cluster-level Contrastive Loss:** This variant excludes the cluster-level contrastive component, retaining only the local contrast as the optimization objective.

**Table 9:** Comparative results of ablation experiments in LRSSL dataset.

Model Variant	Loss	Accuracy	Precision	Recall	F1 Score	AUC
Full Model	**0.2814**	**0.99877**	**0.7247**	**0.9922**	**0.8510**	**0.9347**
w/o High-order Heterogeneous Structure	1.09994	0.63682	0.2808	0.9597	0.4345	0.8672
w/o Structural Interaction Alignment	0.84918	0.37407	0.2855	0.9329	0.5456	0.8847
w/o Hierarchical Contrastive Loss	0.88720	0.28183	0.1914	0.9022	0.2459	0.8719
w/o Cluster-level Contrastive Loss	0.82725	0.46601	0.3781	0.9453	0.5901	0.8867

The quantitative results of these ablation studies on the two datasets are presented in Table 9 and Fig 8, respectively.

The performance of these variants was benchmarked against the full GHF-ACL model using five-fold cross-validation on the HData dataset. The results, summarized in Fig 8, underscore the critical contribution of each constituent module.

In particular, the exclusion of the hypergraph component precipitated a substantial decline in Recall (13.69%) and AUC (0.3445). This confirms that high-order semantic associations among herbs, induced by shared chemical constituents, are indispensable for identifying true positive links between herbs and diseases. Furthermore, replacing the structural alignment module with a naive fusion strategy resulted in the most significant deterioration in performance, with AUC decreasing by 0.4342 and Recall by 33.16%. This suggests that inconsistent representations across structural views significantly impair the model’s discriminative capability regarding associated and unassociated pairs.

Moreover, the removal of hierarchical contrastive learning led to a marked increase in Log Loss (+0.2058) and a reduction in F1 Score (-22.93%), reflecting weakened generalization and a suboptimal trade-off between precision and recall. Notably, ablating the cluster-level contrast exerted a more deleterious impact than removing the local contrast alone, resulting in a 22.14% drop in F1 Score and a 0.3215 decrease in AUC. This indicates that global supervision plays a dominant role in learning reliable herb–disease association patterns.

Overall, the full GHF-ACL model consistently outperforms all ablated variants. These results validate the necessity of integrating high-order structure modeling, attention-guided cross-graph fusion, and hierarchical contrastive learning to achieve robust and interpretable HDA prediction.

### 3.5 Experimental settings and hyperparameter analysis

The proposed model is implemented in Python, and all experiments are conducted under a unified configuration. The embedding dimension *d* is selected from {64, 128, 256}, and is finally set to 128. The learning rate η is searched within {0.001, 0.005, 0.01}, and 0.01 is chosen to ensure stable convergence.

To mitigate overfitting and oversmoothing in the heterogeneous graph, Dropout is applied on both structural and feature representations. Specifically, adjacency Dropout is set to 0.7, and feature Dropout is set to 0.5. The weight of the similarity loss λ is selected from {1, 3, 6, 10}, with the optimal value determined as 6. The maximum number of training epochs is set to 5000 to ensure sufficient optimization. Empirically, the model converges around 700 epochs, after which performance stabilizes with negligible improvement, indicating a well-behaved training process. To guarantee reproducibility, all experiments adopt a fixed random seed of 42.

Herb features are represented by 642-dimensional vectors, obtained by concatenating 321-dimensional chemical features with 321-dimensional similarity-based descriptors. Disease features are represented by 1712-dimensional vectors, derived from disease semantic similarity.

Notably, all hyperparameters are selected based on a combination of common practices in graph representation learning and empirical validation. In particular, the embedding dimension balances representational capacity and overfitting risk; Dropout rates are adjusted to alleviate oversmoothing in dense graph structures; and the similarity loss weight is introduced to balance structural learning and representation alignment, with its value determined through validation experiments.

**Fig 9:**
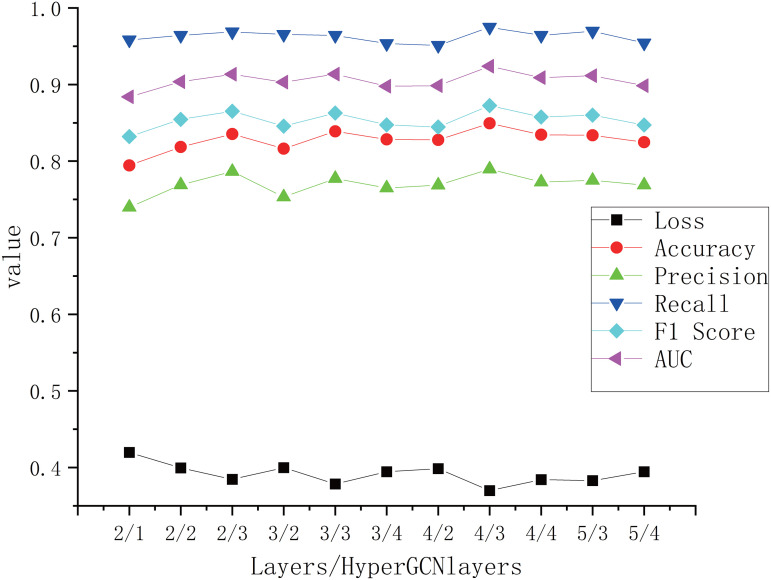
Experimental results with different layers.

To investigate the impact of model depth, we conducted combinatorial experiments by varying the number of GCN layers from 2 to 5 and HyperGCN layers from 1 to 4. The corresponding performance variations are illustrated in Fig 9. Empirical results indicate that the model achieves optimal performance, particularly in terms of AUC and AUPR, when configured with 4 GCN layers and 3 HyperGCN layers. This suggests that this specific depth combination facilitates the capture of richer semantics within the graph structure.

Within the structural interaction module, the adaptive gating mechanism outperforms naive aggregation (i.e., direct concatenation), demonstrating superior representational consistency across structural views.

**Fig 10:**
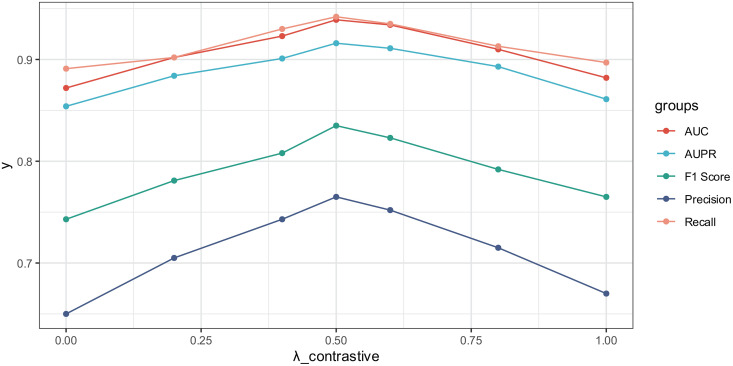
Experimental results with different weights λ_contrastive.

Furthermore, multi-order structural alignment with contrastive learning was employed to refine the consistency of herb and disease embeddings. Both cross-view contrastive learning loss and supervised prediction loss were introduced to model global herb–disease associations and local herb–chemical relationships, respectively. A grid search strategy was used to explore $\lambda_{\text{contrastive}}$, with performance evaluated via cross-validation using AUC and AUPR. The performance trends under different settings are presented in Fig 10. The optimal contrastive loss weight was determined to be 0.5, which balances the contrastive loss and the supervised prediction loss and effectively enhances the alignment of herb and disease embeddings.

## 4 Case study

To demonstrate the practical utility of GHF-ACL, the model was employed to predict associations within unlabelled herb–disease pairs. The top fifteen candidates, ranked by their confidence scores, are presented in Table 10. In the following section, we provide a detailed analysis of the highest-ranked herb–disease pair.

**Table 10:** Top 15 predicted herb–disease associations by GHF-ACL with the highest recommendation scores.

Herb	Disease Name	Recommendation Score
Niuxi	Skin Neoplasms	0.9750
Baqia	Chronic Periodontitis	0.9675
Shiwei	Prosopagnosia	0.9673
Kunbu	Infertility, Female	0.9667
Zhebeimu	Myotonia	0.9643
Kunbu	Primary Myelofibrosis	0.9508
Manjingzi	Hoarseness	0.9507
Huhuanglian	Mitochondrial Diseases	0.9424
Aiye	Anemia, Iron-Deficiency	0.9422
Lulutong	Amblyopia	0.9238
Baibu	Kallmann Syndrome	0.9190
Kudiding	Gastroesophageal Reflux	0.9032
Yadanzi	Renal Insufficiency, Chronic	0.8784
Badou	Pneumothorax	0.8664
Chuanxiong	Dementia	0.8500

It should be noted that all of these top-15 predicted associations do not exist in the original training set, eliminating any possibility of data leakage. From the perspective of the hypergraph, i.e., in the high-order embedding space, NiuXi is semantically closer to herbs known to treat skin diseases or skin tumors (including Yuanhua, Hai Feng Teng, and Xinyi) than in the first-order embedding space. This may indirectly guide the model to infer its potential association with skin tumors.

Niuxi (Achyranthes bidentata Radix), derived from the dried roots of Achyranthes bidentata, is a prominent herb in Traditional Chinese Medicine. The roots are typically harvested in winter following the senescence of stems and leaves, then processed to remove soil and rootlets before being sun-dried. In TCM theory, Niuxi is reputed for its ability to tonify the liver and kidneys, strengthen the musculoskeletal system, and promote the downward movement of blood; it is widely prescribed for conditions such as rheumatic arthralgia and weakness in the lower back and knees.

In recent years, modern pharmacological research has highlighted the potential of Niuxi in oncology, particularly regarding endocrine tumors. Alamri et al. [36] demonstrated that polysaccharides isolated from A. bidentata exhibit immunomodulatory, antitumor, and hepatoprotective activities, specifically targeting the highly mutated driver genes BRAF and NRAS in thyroid cancer. Notably, BRAF and NRAS are also the most prevalent driver mutations in melanoma. Furthermore, a study by Dong et al. [37] indicated a potential causal association between thyroid dysfunction and chronic cutaneous malignant melanoma.

Corroborating these findings, our model predictions support this biological hypothesis. Among all unlabelled herb–disease associations, the Niuxi–Skin Neoplasms pair achieved the highest prediction score. This suggests that Niuxi may exert a therapeutic effect on skin cancers, such as melanoma, potentially through the modulation of shared BRAF/NRAS-related signaling pathways.

## 5 Conclusion

Research on herb–disease association (HDA) prediction has historically been constrained by data scarcity and the complexity of structural patterns. To address this, we constructed the first standardized and reproducible benchmark dataset for HDA by structuring heterogeneous information regarding herbal components, properties, and disease associations, thereby filling a critical gap in public resources.

Building on this foundation, we propose GHF-ACL, a novel framework that integrates higher-order structural modeling with multi-level semantic contrastive learning. This approach effectively aligns cross-view representations while preserving the intrinsic complexity of herbal data. Furthermore, a structural semantic interaction module is introduced to dynamically fuse herbal features across graph orders, alleviating issues of information redundancy and inconsistency.

Extensive experiments demonstrate that GHF-ACL achieves superior performance in AUPR, Recall, and F1 Score on both herbal and cross-domain datasets, demonstrating particular strength in identifying true positive associations under class-imbalanced conditions. Ablation studies further validate the efficacy of each individual module. Overall, this work presents significant innovations in both data resource construction and methodological design, establishing a solid foundation for future HDA research and promoting the modernization of Traditional Chinese Medicine (TCM) and drug discovery.

## 6 Discussion

The strong performance of GHF-ACL can be attributed to its ability to jointly model higher-order structural relationships and semantic consistency across multiple views. Unlike traditional pairwise graph representations, the hypergraph formulation captures complex interactions among herbs and their constituent components, enabling a more comprehensive characterization of herbal properties. In addition, the contrastive learning strategy enhances representation robustness by aligning embeddings from different structural and semantic perspectives. The structural semantic interaction module further facilitates effective information integration across graph orders, reducing redundancy and improving feature consistency. These design choices collectively contribute to the improved predictive performance observed in our experiments.

Nevertheless, while the hypergraph-based representation enables the modeling of higher-order relationships between herbs and their constituent components, it inherently relies on the assumption that shared chemical components may indicate potential functional similarity among herbs. However, in real-world scenarios, the therapeutic effects of herbs are also influenced by factors such as dosage, synergistic interactions, and specific biological contexts.

Recent studies have demonstrated that heterogeneous information networks and geometric deep learning are highly effective in modeling complex biomedical associations. Inspired by these advances, future research could consider extending our framework by incorporating structure-aware mechanisms and biologically informed representation learning, thereby further enhancing the model’s interpretability and generalization capability.

## References

[1] Cheung F. TCM: made in China. Nature. 2011;480(7378):S82-3. 10.1038/480S82a 22190085

[2] Li Y, Liu X, Zhou J, Li F, Wang Y, Liu Q. Artificial intelligence in traditional Chinese medicine: advances in multi-metabolite multi-target interaction modeling. Front Pharmacol. 2025;16:1541509. doi: 10.3389/fphar.2025.1541509 40303920 PMC12037568

[3] Tang J-L, Liu B-Y, Ma K-W. Traditional Chinese medicine. Lancet. 2008;372(9654):1938–40. doi: 10.1016/S0140-6736(08)61354-9 18930523

[4] Zhang R, Zhu X, Bai H, Ning K. Network Pharmacology Databases for Traditional Chinese Medicine: Review and Assessment. Front Pharmacol. 2019;10:123. doi: 10.3389/fphar.2019.00123 30846939 PMC6393382

[5] Yang Z, Xu L, Zhao L. EFBH: Collaborative Filtering Model Based on Multi-Hypergraph Encoder. IEEE Trans Consumer Electron. 2024;70(1):2939–48. doi: 10.1109/tce.2023.3319632

[6] Min Y, Wenkel F, Wolf G. Scattering GCN: Overcoming Oversmoothness in Graph Convolutional Networks. Adv Neural Inf Process Syst. 2020;33:14498–508. 37337543 PMC10277640

[7] Nguyen T, Le H, Quinn TP, Nguyen T, Le TD, Venkatesh S. GraphDTA: predicting drug-target binding affinity with graph neural networks. Bioinformatics. 2021;37(8):1140–7. doi: 10.1093/bioinformatics/btaa921 33119053

[8] Wan F, Hong L, Xiao A, Jiang T, Zeng J. NeoDTI: neural integration of neighbor information from a heterogeneous network for discovering new drug-target interactions. Bioinformatics. 2019;35(1):104–11. doi: 10.1093/bioinformatics/bty543 30561548

[9] Jin S, Hong Y, Zeng L, Jiang Y, Lin Y, Wei L, et al. A general hypergraph learning algorithm for drug multi-task predictions in micro-to-macro biomedical networks. PLoS Comput Biol. 2023;19(11):e1011597. doi: 10.1371/journal.pcbi.1011597 37956212 PMC10681315

[10] Cui S, Li Q, Li D, Lian Z, Hou J. Hyper-Mol: Molecular Representation Learning via Fingerprint-Based Hypergraph. Comput Intell Neurosci. 2023;2023:3756102. doi: 10.1155/2023/3756102 36776618 PMC9908364

[11] Wang N, Li P, Hu X, Yang K, Peng Y, Zhu Q, et al. Herb Target Prediction Based on Representation Learning of Symptom related Heterogeneous Network. Comput Struct Biotechnol J. 2019;17:282–90. doi: 10.1016/j.csbj.2019.02.002 30867892 PMC6396098

[12] Hu L, Zhang M, Hu P, Zhang J, Niu C, Lu X, et al. Dual-channel hypergraph convolutional network for predicting herb-disease associations. Brief Bioinform. 2024;25(2):bbae067. doi: 10.1093/bib/bbae067 38426326 PMC10939431

[13] You Y, Chen T, Wang Z, Shen Y. L2-GCN: Layer-Wise and Learned Efficient Training of Graph Convolutional Networks. In: 2020 IEEE/CVF Conference on Computer Vision and Pattern Recognition (CVPR), 2020. 2124–32. 10.1109/cvpr42600.2020.00220

[14] Liao X, Xu Y, Ling H. Hypergraph Neural Networks for Hypergraph Matching. In: 2021 IEEE/CVF International Conference on Computer Vision (ICCV), 2021. 1246–55. 10.1109/iccv48922.2021.00130

[15] Wang X, Qi G-J. Contrastive Learning With Stronger Augmentations. IEEE Trans Pattern Anal Mach Intell. 2023;45(5):5549–60. doi: 10.1109/TPAMI.2022.3203630 36049010

[16] Feng Y, You H, Zhang Z, Ji R, Gao Y. Hypergraph Neural Networks. AAAI. 2019;33(01):3558–65. doi: 10.1609/aaai.v33i01.33013558

[17] You Y, Chen T, Sui Y, Chen T, Wang Z, Shen Y. Graph contrastive learning with augmentations. Advances in neural information processing systems. 2020;33:5812–23.

[18] Costa LF. Further generalizations of the Jaccard index. arXiv preprint. 2021. doi: 10.48550/arXiv.211009619

[19] Kim J, Bang C, Hwang H, Kim D, Park C, Park S. IMA: Identifying disease-related genes using MeSH terms and association rules. J Biomed Inform. 2017;76:110–23. doi: 10.1016/j.jbi.2017.11.009 29155333

[20] Mathur S, Dinakarpandian D. Finding disease similarity based on implicit semantic similarity. J Biomed Inform. 2012;45(2):363–71. doi: 10.1016/j.jbi.2011.11.017 22166490

[21] Nakazato T, Bono H, Matsuda H, Takagi T. Gendoo: functional profiling of gene and disease features using MeSH vocabulary. Nucleic Acids Res. 2009;37(Web Server issue):W166-9. doi: 10.1093/nar/gkp483 19498079 PMC2703956

[22] Zhu S, Zeng J, Mamitsuka H. Enhancing MEDLINE document clustering by incorporating MeSH semantic similarity. Bioinformatics. 2009;25(15):1944–51. doi: 10.1093/bioinformatics/btp338 19497938

[23] Chen H, Guo R, Li G, Zhang W, Zhang Z. Comparative analysis of similarity measurements in miRNAs with applications to miRNA-disease association predictions. BMC Bioinformatics. 2020;21(1):176. doi: 10.1186/s12859-020-3515-9 32366225 PMC7199309

[24] Hu Y, Li X, Wang Y, Wu Y, Zhao Y, Yan C, et al. Adaptive Hypergraph Auto-Encoder for Relational Data Clustering. IEEE Trans Knowl Data Eng. 2021;:1–1. doi: 10.1109/tkde.2021.310819236506788

[25] Fan H, Zhang F, Wei Y, Li Z, Zou C, Gao Y, et al. Heterogeneous Hypergraph Variational Autoencoder for Link Prediction. IEEE Trans Pattern Anal Mach Intell. 2022;44(8):4125–38. doi: 10.1109/TPAMI.2021.3059313 33587699

[26] Gao Y, Feng Y, Ji S, Ji R. Hgnn : General hypergraph neural networks. IEEE Transactions on Pattern Analysis and Machine Intelligence. 2022;45(3):3181–99. 10.1109/TPAMI.2022.3182052 35696461

[27] Pei H, Wei B, Chang KCC, Lei Y, Yang B. Geom-gcn: geometric graph convolutional networks. arXiv preprint. 2020. doi: 10.48550/arXiv.2002.05287

[28] Yu Z, Huang F, Zhao X, Xiao W, Zhang W. Predicting drug-disease associations through layer attention graph convolutional network. Brief Bioinform. 2021;22(4):bbaa243. doi: 10.1093/bib/bbaa243 33078832

[29] Huang S, Wang M, Zheng X, Chen J, Tang C. Hierarchical and dynamic graph attention network for drug-disease association prediction. IEEE J Biomed Health Inform. 2024;PP:10.1109/JBHI.2024.3363080. doi: 10.1109/JBHI.2024.3363080 38319783

[30] Meng Y, Lu C, Jin M, Xu J, Zeng X, Yang J. A weighted bilinear neural collaborative filtering approach for drug repositioning. Brief Bioinform. 2022;23(2):bbab581. doi: 10.1093/bib/bbab581 35039838

[31] Jin Z, Zheng X, Zhou H, Ji C, Xiang S, Tang C. DD-HGNN^+^: Drug-Disease Association Prediction via General Hypergraph Neural Network With Hierarchical Contrastive Learning and Cross Attention Learning. IEEE J Biomed Health Inform. 2025;29(11):7810–9. doi: 10.1109/JBHI.2025.3542784 40036538

[32] Sun X, Jia X, Lu Z, Tang J, Li M. Drug repositioning with adaptive graph convolutional networks. Bioinformatics. 2024;40(1):btad748. doi: 10.1093/bioinformatics/btad748 38070161 PMC10761094

[33] Meng Y, Wang Y, Xu J, Lu C, Tang X, Peng T, et al. Drug repositioning based on weighted local information augmented graph neural network. Brief Bioinform. 2023;25(1):bbad431. doi: 10.1093/bib/bbad431 38019732 PMC10686358

[34] Veleiro U, de la Fuente J, Serrano G, Pizurica M, Casals M, Pineda-Lucena A, et al. GeNNius: an ultrafast drug-target interaction inference method based on graph neural networks. Bioinformatics. 2024;40(1):btad774. doi: 10.1093/bioinformatics/btad774 38134424 PMC10766589

[35] Qiao Y, Hu L, Zhang J, Hu P, Luo X. Identifying novel therapeutic targets of natural compounds in traditional Chinese medicine herbs with hypergraph representation learning. Brief Bioinform. 2025;26(4):bbaf399. doi: 10.1093/bib/bbaf399 40794950 PMC12342802

[36] Alamri AM, Alkhilaiwi FA, Khan NU, Tasleem M. In silico Screening and Validation of Achyranthes aspera as a Potential Inhibitor of BRAF and NRAS in Controlling Thyroid Cancer. Anticancer Agents Med Chem. 2023;23(19):2111–26. doi: 10.2174/1871520623666230607125258 37287303

[37] Dong H, Pan L, Shen Y, Xu Q, Hu J, Hu Z, et al. Thyroid dysfunction and risk of cutaneous malignant melanoma: a bidirectional two-sample Mendelian randomization study. Front Endocrinol (Lausanne). 2023;14:1239883. doi: 10.3389/fendo.2023.1239883 38093968 PMC10716543

