# Supplementary material for: GHF-ACL: A novel contrastive learning framework with multi-order graph structures for herb-disease association prediction
Source: PLoS Comput Biol. 2026 Jun 29;22(6):e1014461. doi: 10.1371/journal.pcbi.1014461 (PMC13327522; doi:10.1371/journal.pcbi.1014461)
Supplement: Attachment — Submitted filename: Response to Reviewers.docx [file pcbi.1014461.s002.docx]

Dear Editor and Reviewers,

We would like to express our sincere gratitude for your insightful and constructive comments on our manuscript. We highly appreciate the time and effort you have devoted to reviewing our work.

In response to the reviewers’ comments, we have thoroughly revised the manuscript and improved it significantly. All modifications have been clearly marked in the revised version. Detailed responses to each comment are provided below.

During the revision, Xiuhong Wu has been included as a co-author due to her contributions to data analysis and manuscript improvement. As an expert in Traditional Chinese Medicine, she also provided valuable domain-specific insights that strengthened the interpretation of the results. All authors have approved this change. The updated author contributions have also been added to the submission system accordingly.

In addition, we have uploaded the complete LaTeX manuscript (.tex file) and provided all figures in .eps format as required. For the images with potential copyright concerns, we have replaced the original clip-art with appropriate alternatives from the resources recommended by the editor to ensure compliance with the journal’s licensing policy.

We hope that the revised manuscript meets the journal’s standards and your expectations.

Reviewer #1:

**Comment** **1**: the primary contribution of this work is framed around the introduction of a new dataset, hdata, yet the manuscript fails to provide a rigorous justification for its necessity or a comparative analysis against existing resources beyond basic statistical sparsity metrics reported in table 2 you must substantially expand the dataset description to include the sources and preprocessing steps for each data type (herbal properties, chemical compositions, and disease associations) with far greater detail additionally a direct quantitative and qualitative comparison against the existing datasets used in the baselines (e.g., lrsl, cdata, edata) is required to demonstrate that hdata offers unique challenges or information not already captured this is not merely a matter of curation but of establishing a new benchmark standard you need to clearly articulate what specific limitations in existing data hdata overcomes and ideally provide a side-by-side feature comparison table to justify its creation and adoption by the community

**Response**: We would like to thank the reviewers for their valuable suggestions regarding the rigor of our dataset construction; we fully agree that the creation of a benchmark dataset requires a more detailed description. We have therefore included a detailed account of the data pre-processing steps in the manuscript. This new section describes the sources from which the data was collected, as well as the standardisation of Chinese herbal medicine nomenclature, the verification and deduplication of chemical constituents, and the alignment of disease terminology during the construction of the dataset. To facilitate a more intuitive comparison with existing datasets, we have introduced a table comparing the functional attributes of various datasets. Existing datasets are primarily derived from a single database and contain only a small number of natural chemical constituents, with the remainder comprising synthetic small-molecule drugs; the drug data within these datasets consists almost exclusively of single-molecule drugs. In contrast, the Hdata dataset constructed in this paper takes “The Chinese Materia Medica” as its core reference, collecting herb-disease association data from multiple Chinese herbal medicine databases. It contains only natural herbal data, and the chemical constituents within the Chinese herbal medicines have undergone bioactivity screening and organization. Finally, researchers specialising in traditional Chinese medicine were invited to conduct manual spot-checks of the collected associations to ensure the accuracy of the dataset. Lines 78–96, where a detailed description of the dataset construction has been added.

**Comment** **2**: the core methodological novelty of your multi-order graph structure is undermined by a lack of clarity and, in some cases, what appears to be mathematical inconsistency in its formulation when you define the unified adjacency matrix a_h in equation 4 you combine normalized similarity matrices with the association matrix a however the notation for the disease side uses ~d^{-1/2}_d s_d ~d^{-1/2}_d without defining ~d_d or its relationship to the previously defined degree matrices this ambiguity makes it impossible to reproduce the construction of this critical input furthermore the description of the hypergraph construction in section 2.2.1 states that the hypergraph adjacency matrix a_hyper is obtained via a_hyper = h w h^t but then introduces figure 2 and text discussing clique expansion with uniform edge weighting it is imperative that you clarify whether a_hyper is used directly as a weighted graph adjacency matrix for a standard gcn or if this is the first step in a true hypergraph convolution you must provide the exact mathematical form of the input to the hypergcn module and reconcile it with the propagation rule defined later in equations 7 and 8.

**Response**: We would like to thank the reviewers for pointing out the ambiguity in the definition of the unified adjacency matrix; we do indeed lack a clear definition of the symbol D_d_ in the manuscript. Specifically, this normalization method is used to ensure the numerical stability of the Laplace operator during propagation; it involves summing each row of the two similarity matrices to obtain their respective diagonal matrices D. We have comprehensively revised the notation, standardising it to the normalized form, and have explicitly defined the degree matrices D_h_ and D_d_ for the herbal similarity matrix S_h_ and the disease similarity matrix S_d_, respectively:

$D_{h}\left( i,i \right)=\sum_{j} S_{h}\left( i,j \right)$,$D_{d}\left( i,i \right)=\sum_{j} S_{d}\left( i,j \right)$

Therefore, the heterogeneous adjacency matrix can be expressed as $A_{H}=\left( \begin{matrix} D_{h}^{-\frac{1}{2}}S_{h}D_{h}^{-\frac{1}{2}} & A \\ A^{T} & D_{d}^{-\frac{1}{2}}S_{d}D_{d}^{-\frac{1}{2}} \end{matrix} \right)$, This explanation ensures that the normalization scheme is mathematically consistent with the standard symmetric normalization scheme used in graph convolutional networks.

Furthermore, we also appreciate the reviewers' observation regarding the potential ambiguity between the hypergraph adjacency structure and the cluster expansion description. In the original text, the expression $A_{hyper}=HWH^{T}$, this is intended to provide an intuitive interpretation of the hypergraph structure, which corresponds to a cluster expansion representation in which herbs with the same chemical composition are interconnected via links weighted by $\frac{1}{d_{k}}$; it does not directly construct the hypergraph adjacency matrix. In the actual model, hypergraph convolution is implemented using the normalized hypergraph Laplacian operator defined in the modified Equation (7). This formula adheres to the standard HyperGCN spectral convolution framework and explicitly models higher-order relationships, rather than simplifying the hypergraph to a simple pairwise graph. We have added a corresponding explanation in Section 2.2.1 to clarify this distinction and avoid any misunderstanding (Lines 163-168,185-201).

Further details regarding the specific mathematical formulation of Formula 7—including the constructed hypergraph matrix H and weights W, as well as the vertex degree matrix Dv and hyperedge degree matrix De derived from H—have been added in Section 2.2.2 (Lines 236-239).

**Comment** 3. your description of the hypergraph convolution operation in section 2.2.2 contains serious technical inaccuracies and oversimplifications regarding spectral-based methods you state that the laplacian l = i - d_v^{-1/2} h w d_e^{-1} h^t d_v^{-1/2} is used to perform low-pass filtering in the fourier domain to capture global community structure and that the weight matrix w is adjusted by structural bias in accordance with the tf-idf principle this is a vague and misleading explanation first the equation provided is a specific formulation for a hypergraph laplacian but calling it a spectral convolution without defining the actual fourier basis or explaining how the filtering is achieved beyond a single layer operation is insufficient second the analogy to tf-idf is not mathematically defined within the equation or its description you must provide a clear, step-by-step mathematical derivation of how the forward pass of your hypergcn layer works starting from the incidence matrix h, defining the propagation rule explicitly (e.g., z^{(l+1)} = σ( d_v^{-1/2} h w d_e^{-1} h^t d_v^{-1/2} z^{(l)} θ^{(l)} )) and then explaining how this specific formulation achieves the claimed effect of attenuating high-frequency components or weighting hyperedges by their uniqueness the current description is not computationally reproducible

**Response**: We agree that the original description conflated the hypergraph Laplacian with the spectral convolution operator, and that the hypergraph Fourier basis was never explicitly defined. We have corrected this as follows.

The normalized hypergraph Laplacian $L=I-D_{V}^{-1/2}HWD_{E}^{-1}H^{\top}D_{V}^{-1/2}$ defines the frequency axis of the hypergraph via its eigendecomposition $L=U\Lambda U^{\top}$, where the eigenvector matrix $U$ constitutes the hypergraph Fourier basis and the eigenvalues $\lambda_{k}\in[0,2]$ correspond to frequency components. The Fourier transform of a hypergraph signal $f$ is defined as $\hat{f}=U^{\top}f$. The actual convolution is performed via a first-order polynomial filter:

$$g_{\theta}(\lambda_{k})=1-\lambda_{k}$$

The corresponding spatial propagation operator is:

$$\Theta=I-L=D_{V}^{-1/2}HWD_{E}^{-1}H^{\top}D_{V}^{-1/2}$$

We have explicitly stated in the revised draft that low-pass characteristics are properties of the filter response function, rather than properties of the Laplace operator L itself; we have also provided a complete definition of the hypergraph Fourier basis, while optimize strictly distinguishing the respective roles of $L$ and the propagation operator $\Theta$.

We acknowledge that the forward propagation formula in the original paper is incomplete. The revised version now includes a complete step-by-step derivation; the specific process of forward propagation in the $l$ th layer of HyperGCN comprises the following three steps. First, Hyperedge aggregation: For each hyperedge $e_{j}$, the normalized mean of the herb embeddings of its members is calculated:

$$M^{\left( l \right)}=D_{E}^{-1}H^{\top}Z^{\left( l \right)}\in\mathbb{R}^{m\times d_{l}}$$

Second, Weighted reverse distribution, each herb node receives weighted aggregated information from its incident hyperedges:

$$\tilde{Z}^{\left( l \right)}=HWD_{E}^{-1}H^{\top}Z^{\left( l \right)}\in\mathbb{R}^{N\times d_{l}}$$

Third, Symmetric normalization and linear transformation:

$$Z^{\left( l + 1 \right)}=\sigma\left( D_{V}^{-1/2}HWD_{E}^{-1}H^{\top}D_{V}^{-1/2}\text{ }Z^{\left( l \right)}\text{ }\Theta^{\left( l \right)} \right),Z^{\left( 0 \right)}=X_{h}$$

where $X_{h}\in\mathbb{R}^{N\times d}$ is the initial herb feature matrix, $\Theta^{\left( l \right)}\in\mathbb{R}^{d_{l}\times d_{l+1}}$is the learnable weight matrix at layer $l$, and $\sigma$is a nonlinear activation function. We have also added the node-level expansion of the propagation rule to make the aggregation mechanism fully explicit:

$$z_{i}^{\left( l + 1 \right)}=\sigma\left( \frac{1}{d_{v_{i}}^{1/2}}\sum_{j:\text{ }v_{i}\in e_{j}} \frac{1}{\delta_{e_{j}}^{2}}\sum_{k:\text{ }v_{k}\in e_{j}} \frac{z_{k}^{\left( l \right)}}{d_{v_{k}}^{1/2}} \cdot\Theta^{\left( l \right)} \right)$$

This makes explicit that node $v_{i}$ aggregates embeddings of all herbs $v_{k}$ sharing the same hyperedge $e_{j}$, weighted by $1/\delta_{e_{j}}^{2}$, which naturally suppresses the dominance of large hyperedges and gives the rare shared components specific to a particular formula a relatively stronger semantic representation.

We fully agree that it is inappropriate to refer to the TF-IDF principle without providing a precise mathematical definition. We have therefore removed all references to TF-IDF from the revised draft and replaced them with an explanation of structural normalization that is entirely consistent with the definition provided earlier in this paper.

Specifically, the hyperedge weight matrix is defined as:

$$W=\mathrm{diag}\left( \frac{1}{\delta_{e_{1}}},\ldots,\frac{1}{\delta_{e_{m}}} \right)$$

where $\delta_{e_{j}}=\sum_{i} H_{ij}$ is the degree of hyperedge $e_{j}$, i.e., the number of herbs containing constituent $e_{j}$. This inverse-degree weighting ensures that large hyperedges (constituents shared by many herbs) contribute less per connection, while small hyperedges (formula-specific constituents shared by few herbs) contribute more. Combined with the $D_{E}^{-1}$ normalization already present in the propagation operator, the effective weight of hyperedge $e_{j}$ is:

$$\frac{w_{j}}{\delta_{e_{j}}}=\frac{1}{\delta_{e_{j}}^{2}}$$

**Comment** 4:the attention-guided semantic interaction module described in section 2.2.3 and equations 9-12 raises significant concerns about its intended functionality and the dimensional consistency of its operations you define an attention score ~α_i = w^t · tanh(w1 h^g_i + w2 h^h_i + b) where h^g_i and h^h_i are the final layer embeddings from the gcn and hypergcn respectively for a single herb node i the output ~α_i is then passed through sigmoid and softmax to produce a final scalar weight α_i used to fuse the representations this appears to calculate a single, global attention weight per node based on its own features, which is not a standard cross-attention mechanism between the two views you must clarify what the vectors w, w1, and w2 are and how their dimensions align to produce a scalar from the concatenated or summed input features furthermore the application of softmax over a neighborhood n_i in equation 11 for a scalar value derived from a single node is conceptually confusing you need to provide a much more detailed explanation of this mechanism, possibly with a diagram showing the tensor dimensions at each step, to demonstrate that it is not simply learning a node-specific bias but is truly aligning the two structural representations.

**Response**: Thank you for your insightful comments on Sections 2.2.3 and 2.2.4. We have carefully reviewed the wording in this section and found that there are indeed some ambiguities in the explanation of the mechanism and the definition of the dimensions. Based on your suggestions, we have substantially revised and unified these sections into a single coherent module to improve clarity, rigor, and consistency. We agree that the original description could be misinterpreted as a standard cross-attention mechanism. The objective of this module is to achieve adaptive fusion and alignment between different structural views (GCN and HyperGCN) of the same herb node, rather than modeling interaction relationships between different nodes. To address the issue of unclear definitions of symbols (e.g., $w$, $W_{1}$, $W_{2}$) in the original formulation, we have adopted a more standardized and explicit representation in the revised manuscript (Lines 292-306).

For each herb node $i\in H$:

$$z_{i}^{\left( g \right)},z_{i}^{\left( h \right)}\in\mathbb{R}^{d}$$

The attention weight is computed as:

$$\alpha_{i}=\sigma(W[z_{i}^{\left( g \right)}\text{ }\parallel\text{ }z_{i}^{\left( h \right)}])$$

Where:

$$[z_{i}^{\left( g \right)}\text{ }\parallel\text{ }z_{i}^{\left( h \right)}]\in\mathbb{R}^{2d}$$

$$W\in\mathbb{R}^{1\times2d}$$

Thus, $\alpha_{i}$ is a scalar, which ensures dimensional consistency. The fused representation is:

$$z_{i}=\alpha_{i}z_{i}^{\left( g \right)}+(1-\alpha_{i})z_{i}^{\left( h \right)}$$

All parameters are shared across nodes, ensuring that the model learns structure-aware fusion patterns rather than node-specific parameters.

We thank the reviewer for pointing out the issue regarding the use of softmax. The original description of applying softmax over neighborhoods was indeed not sufficiently rigorous. In the revised manuscript, we have completely removed this statement and clarified that our method adopts a node-level gating mechanism (sigmoid), without involving any neighborhood-level normalization.

To prevent this module from degenerating into a simple node-specific weighting scheme, we further introduce contrastive learning as an alignment constraint in the revised version (lines 307-323). Specifically, we employ a local contrastive learning loss:

$$L_{con}=-\sum_{i} \log\frac{\exp(sim(z_{i}^{\left( g \right)},z_{i}^{\left( h \right)})/\tau)}{\exp(sim(z_{i}^{\left( g \right)},z_{i}^{\left( h \right)})/\tau)+\sum_{k\neq i} \exp(sim(z_{i}^{\left( g \right)},z_{k}^{\left( h \right)})/\tau)}$$

This loss function enforces the attention mechanism to operate within an aligned semantic space. Consequently, contrastive learning functions not only as an auxiliary supervision signal but also as a regularizer for the attention fusion process, effectively mitigating the risk of degeneration into node-specific bias.

**Comment** **5**: the hierarchical contrastive learning section introduces both cluster-level (binary cross-entropy) and local-level (contrastive) losses but the interaction and purpose of these two levels are not clearly delineated you refer to the binary cross-entropy in equation 13 as "cluster-level" contrast, but this is simply the standard supervised loss for the prediction task and is not a contrastive loss in the traditional sense (like info-nce) this conflates the objectives and misrepresents the architecture you should rename these components to avoid confusion for instance, calling l_bce the "supervised prediction loss" and l_local the "cross-view contrastive loss" would be far more accurate furthermore, the formulation of l_local in equation 14 is for a single positive pair but it is unclear how negative samples are constructed within a batch you must specify whether negative sampling is performed across nodes within the same view, across views, or both, and how this sampling strategy impacts the learned representations.

**Response**: We agree that the original manuscript’s classification of binary cross-entropy loss under hierarchical contrastive learning was not sufficiently rigorous. In the revised manuscript, we have clearly distinguished between the two types of objectives. Supervised prediction loss ($L_{\text{pred}}$) used to directly optimize the herbal medicine–disease association prediction task; cross-view contrast loss ($L_{\text{con}}$) used to align the node representations learned by GCN and HyperGCN. Through the renaming and functional division outlined above, we have established that $L_{\text{pred}}$ is responsible for prediction tasks, while $L_{\text{con}}$ is responsible for representation alignment; the two are optimized collaboratively within a unified framework. The revised $L_{\mathrm{con}}$ adopts an InfoNCE-based contrastive objective, which maximizes the consistency of representations of the same node across different structural views while minimizing the similarity between representations of different nodes, thereby achieving alignment across structural semantic spaces.

In the revised manuscript, we further provide a detailed description of the negative sampling strategy (lines 399-407). Specifically, for each positive pair $\left( z_{i}^{\left( g \right)} , z_{i}^{\left( h \right)} \right)$ within a mini-batch, we employ a 1:10 negative sampling scheme. That is, we randomly sample $n$ representations $z_{k}^{\left( h \right)}$from different nodes ($k\neq i$) as negative samples.

**Comment** **6**:the experimental comparison against baseline models is severely flawed by the inclusion of two variants of the same method (htinet-knn and htinet-rf) which are not state-of-the-art deep learning models but rather simple classifiers applied to node2vec features including these weak baselines artificially inflates the perceived performance of your model, as evidenced by their extremely poor precision and f1 scores in table 5 you must replace these with more recent and competitive baselines for graph-based association prediction such as mgat (multi-channel graph attention network), dgedti (dual-graph ensemble learning), or other relevant heterogeneous graph transformers a fair comparison should pit your complex, multi-order framework against other complex, end-to-end deep learning models, not against a two-year-old method using a random forest classifier this is a critical requirement for a valid experimental setup in a top-tier journal.

**Response**: We thank the reviewer for the constructive suggestion to include stronger and more competitive baseline models.

In the revised manuscript, we have incorporated two representative state-of-the-art deep learning models for a more rigorous comparison:
(1) GeNNius, an ultra-fast graph neural network-based method for drug–target interaction inference;
(2) HDCTI, a graph contrastive learning framework for predicting drug–target interactions.

To ensure consistency in task definition and input representation, we adapted GeNNius within a unified framework. Specifically, the original drug and target nodes were mapped to herb and disease nodes, respectively. Based on known herb–disease associations, we constructed a bipartite graph, thereby naturally transforming the original drug–target prediction task into a herb–disease association prediction task. For fairness, GeNNius was trained and evaluated under the same data splits, negative sampling strategy, and evaluation metrics as our proposed method.

The original HDCTI model is designed to predict interactions between natural product compounds and targets, with its core based on hypergraph representation learning to model high-order relationships between “compound–target” pairs. To adapt it to our task, we first performed a relation-level mapping by treating herb–disease associations as analogous to the “compound–target” relationships in HDCTI, thereby aligning the prediction objective without modifying the core model architecture. In terms of node and relation modeling, herbs were treated as compound nodes and diseases as target nodes, with known herb–disease associations forming the bipartite relationships. During training, we followed the original HDCTI paradigm, using known herb–disease associations as positive samples and constructing negative samples via random sampling for binary classification.

To enhance structural representation, we further incorporated herb–herb and disease–disease similarity relationships within a unified framework to assist node representation learning, while preserving the original hypergraph modeling mechanism of HDCTI. For evaluation, to ensure strict fairness, HDCTI was trained and tested under exactly the same data splits, negative sampling strategy, and evaluation protocol as our method. Specifically, all models were evaluated under identical experimental settings to guarantee comparability.

Experimental results demonstrate that, even with the inclusion of these strong baselines, our method consistently achieves superior performance across multiple evaluation metrics, further validating the effectiveness of the proposed multi-order structural modeling and contrastive learning mechanism for herb–disease association prediction.

**Comment** **7**: the analysis of the visualization results in section 3.3, particularly the t-sne plots in figure 7, is superficial and does not substantiate the claims made about the model's capabilities you state that the clustering patterns "underscore the model’s capacity to capture structural information" and "offer further interpretive support," but without any quantitative metric like silhouette score or a clear demonstration of how the clusters correspond to known pharmacological categories (e.g., herbs used for heat-clearing clustering together), these plots are merely qualitative and subjective you must provide a more rigorous analysis of the embedding space for example, you could select a few known functional classes of herbs and diseases and calculate the intra-class vs. inter-class distances in the embedding space to quantitatively demonstrate that the model has indeed learned meaningful semantic groupings this would transform a weak visual anecdote into a strong piece of evidence for representation quality.

**Response**: We would like to thank the reviewers for their constructive feedback. We agree that relying solely on t-SNE visualizations for qualitative analysis is insufficient to fully demonstrate the model’s capabilities in structural representation learning. Consequently, in the revised manuscript, we have significantly expanded Section 3.3, introducing a variety of quantitative evaluation metrics and biological semantic validation to provide a more rigorous analysis of the quality of the embedding space.

The specific improvements are as follows:

The t-SNE visualization is used only as an auxiliary intuitive illustration rather than as the primary evidence of model performance. To improve interpretability, we further employ PCA to visualize the clustering results. Unlike t-SNE, PCA preserves the global structure of the data, making the observed cluster separation more reliable. The clustering metrics indicate a moderate degree of structural organization in the embedding space. Given the complexity and multi-functionality of traditional Chinese medicine, this is expected, as herbs often exhibit overlapping pharmacological properties rather than forming strictly separable categories. Nevertheless, further analysis based on pharmacological classifications and representative herbs demonstrates that these clusters are semantically meaningful.

To further investigate the semantic consistency of the learned embeddings, we manually examined representative herbs that are closest to the cluster centroids. Interestingly, the identified clusters do not strictly align with traditional single-category classifications (e.g., cold vs. warm properties). Instead, they capture higher-level functional patterns that integrate multiple pharmacological characteristics.

For example, one cluster includes herbs such as Dangshen, Huangqi, and Danggui, which are commonly associated with tonic and immunoregulatory functions. In contrast, another cluster contains herbs such as Xianhecao and Jinyinhua, which are primarily used for dispelling wind-dampness and anti-inflammatory purposes. These observations suggest that the learned embeddings are capable of grouping herbs with similar pharmacological properties, providing further evidence that the model captures meaningful biological semantics.

In addition, we computed the average intra-cluster and inter-cluster distances in the embedding space. The intra-cluster distance (0.574) is significantly smaller than the inter-cluster distance (1.110), with a ratio of 0.517, indicating that the learned embeddings preserve meaningful structural separability. Although the clustering metrics suggest that the separation between clusters is not highly pronounced, the embeddings still exhibit a certain degree of structural organization. Importantly, the moderate level of cluster separability does not contradict the strong predictive performance of the model. This is because link prediction primarily relies on pairwise similarity rather than global clustering structure. In the context of traditional Chinese medicine, herbs often exhibit multi-functional properties, leading to overlapping clusters. Nevertheless, their interaction patterns with diseases remain sufficiently distinguishable, enabling accurate prediction.

**Comment** **8**. the case study presented in section 4, while interesting, suffers from a logical gap that weakens its validity as evidence for your model's predictive power you predict that niuxi is associated with skin neoplasms and then cite two separate studies one showing that niuxi polysaccharides target braf/nras in thyroid cancer and another showing a causal link between thyroid dysfunction and melanoma the logical connection you make is that because niuxi targets genes also mutated in melanoma, it may treat melanoma however, this is a post-hoc rationalization based on known literature, not a novel discovery validated by your model the model merely ranked the pair highly to prove your model's utility, you must demonstrate that this specific association (niuxi-skin neoplasms) is not explicitly present in the training data and is not trivially inferred from simple first-order similarities you should perform a deeper analysis perhaps showing that other herbs sharing similar chemical components with niuxi are also predicted to treat skin-related conditions, thereby providing a multi-evidenced, mechanistic hypothesis rooted in your model's high-order structure.

**Response**: Thank you for your insightful comment. We acknowledge that the original case study relied on retrospective, literature-based reasoning, which may not have fully demonstrated the model’s predictive capabilities. To address this issue, we have substantially revised the case study at a structural level.

(1) Data leakage analysis: We first verified that no association exists between Achyranthes root and skin neoplasms in the training dataset, and that such an association cannot be readily inferred through first-order connections; this ensures that the prediction results do not stem from information leakage or simple neighbourhood similarity.

(2) Structural analysis: We further analyzed the embedding space and found that Niuxi is located close to several herbs known to be associated with skin-related diseases or anti-tumor effects. Notably, these herbs do not exhibit strong similarity with Niuxi in the first-order graph, nor do they share exactly the same chemical components. Instead, they demonstrate consistent pharmacological characteristics (e.g., promoting blood circulation), suggesting that the model captures higher-order functional patterns rather than relying on direct similarity. From a hypergraph perspective, compared with the first-order relational space, Niuxi is positioned closer to herbs such as Yuanhua, Haifengteng, and Xinyi in the high-order embedding space. Existing studies have reported that these herbs exhibit therapeutic effects on skin-related diseases or tumor-associated pathological processes, providing additional support for the predicted associations.

(3) Literature evidence: We further reviewed existing biomedical databases and recent studies. A growing body of evidence has reported anti-tumor and anti-inflammatory properties of these herbs, both of which are closely linked to tumor progression. These findings provide indirect but compelling support for the biological plausibility of the predicted associations.

Importantly, none of the top-15 predicted associations are present in the training dataset, highlighting the model’s capacity to generalize beyond observed data and identify potentially novel herb–disease relationships.

**Comment** **9**: the ablation study results presented in table 6 contain numerical inconsistencies that cast doubt on the reliability of the experimental setup for the "w/o high-order heterogeneous structure" variant on the lrsl dataset, you report an accuracy of 0.99877 and a loss of 1.09994 it is highly unusual, if not indicative of a problem, for a model to have such high accuracy while simultaneously having a loss value above 1, especially with a binary cross-entropy loss which typically ranges from 0 to ~0.7 for well-fitted models you must investigate and explain this discrepancy is the model severely overconfident and wrong on certain samples? is the loss calculated on a different scale? this requires immediate clarification and correction as it undermines the credibility of your entire ablation analysis and suggests potential issues with overfitting, numerical instability, or a bug in the evaluation code.

**Response**: Firstly, we need to clarify that the model achieving an accuracy of 0.99877 on the LRRSL dataset is the full model, not the variant ‘w/o higher-order heterogeneous structure’. Due to the layout of the table, this may have led to some misunderstanding; we will revise the table to make the wording clearer.

Even when the model achieves high accuracy, the loss value can remain relatively high if some predictions are made with low confidence, or if a small number of misclassified samples are assigned excessively high (but incorrect) confidence scores.

This phenomenon can be attributed to two main factors:
(1) Class imbalance in the dataset, which may amplify the contribution of such samples to the overall loss;
(2) The loss is computed as the average over all samples, with different loss components directly summed, making it sensitive to high-confidence errors.

Regarding the relatively higher loss observed after removing the structural component, this behavior is consistent with the properties of binary cross-entropy loss, where high-confidence incorrect predictions incur disproportionately large penalties. This suggests that removing the high-order heterogeneous structure not only degrades predictive performance but also negatively affects probability calibration.

We will revise the manuscript to clarify the presentation of the table and provide a more explicit explanation of this observation.

**Comment** **10**: the manuscript suffers from significant issues in scholarly presentation, including a garbled and nonsensical block of text on page 7 that appears to be a formatting error with repeated "1 1 1" sequences and a figure reference that is out of place such technical errors in a submitted manuscript are unacceptable and create a strong impression of carelessness furthermore, there is a clear inconsistency in the abstract where you report a +4.8% improvement on "lrsl" but in the results section, table 4, the baseline model for comparison on this dataset is not specified with an improvement value you must meticulously proofread the entire document to correct all formatting errors and ensure that every claim made in the abstract, introduction, and conclusion is directly and accurately supported by a specific table or figure in the results section the current state of the manuscript does not meet the professional standards expected for publication

**Response**: We sincerely thank the reviewer for this careful and critical assessment. We fully acknowledge that the issues regarding formatting errors and inconsistencies in the manuscript are unacceptable and do not meet the expected standards of academic publication. We apologize for the oversight and any confusion caused.

Although these issues were not apparent in our local version, we recognize that they may arise due to font embedding or encoding inconsistencies across different systems. To address this, we have carefully recompiled and regenerated the manuscript to ensure full compatibility.

Formatting and technical errors: We have thoroughly proofread the entire manuscript and corrected all formatting issues. In addition, we have carefully revised all numerical references, equations, and symbols to ensure consistency and correctness throughout the manuscript.

(1) Consistency between abstract and results: We acknowledge that the description in the abstract regarding the reported improvement on the LRSSL dataset was not sufficiently clear. The reported +4.8% improvement is calculated relative to the best-performing baseline model (DD-HGNN+), which was not explicitly indicated in Table 4 in the original submission.

To address this issue, we have made the following revisions:

First, abstract has been updated to explicitly state that all improvements are reported with respect to the strongest baseline on each dataset; Second, table 5 has been revised to clearly highlight the best-performing baseline for each dataset; Third, clarifications have been added in the results section to ensure that all reported improvements can be directly verified from the corresponding tables.

(2) Overall manuscript quality: We have conducted a comprehensive revision of the manuscript to ensure that all statements in the abstract, introduction, and conclusion are directly and accurately supported by the experimental results (tables and figures).

We appreciate the reviewer’s comments, which have helped us significantly improve the clarity, consistency, and overall quality of the manuscript.

Reviewer #2:

The paper proposes a graph and hypergraph based contrastive learning model for predicting herb–disease associations in Traditional Chinese Medicine. Although the topic is interesting and the model integrates several modern graph learning techniques, the manuscript has several methodological, experimental, and presentation issues that significantly limit the clarity, reproducibility, and scientific rigor of the work. The following major concerns should be addressed before the manuscript can be considered for publication.

**Comment 1**: The novelty of the proposed framework is not sufficiently justified because the model mainly combines already established components such as graph convolutional networks, hypergraph neural networks, attention based fusion, and contrastive learning without clearly demonstrating what fundamentally new methodological contribution is introduced beyond integrating existing techniques.

**Response**: We thank the reviewer for this insightful comment. We agree that our framework incorporates several established techniques, such as graph convolutional networks, hypergraph neural networks, attention-based fusion, and contrastive learning. However, we would like to clarify that the novelty of our work does not lie in the individual components themselves, but in how they are systematically integrated to address the unique challenges of herb–disease association prediction.

Specifically, the novelty of our framework can be summarized in the following aspects:

(1) Novel heterogeneous hypergraph construction strategy.

Unlike conventional pairwise graph modeling, we construct a hypergraph that explicitly captures higher-order relationships among herbs, chemical components, and diseases. This enables the model to represent complex many-to-many interactions that cannot be effectively modeled by standard GNNs.

(2) Cross-view collaborative representation learning mechanism.

We design a dual-branch architecture that jointly models pairwise (GCN-based) and higher-order (hypergraph-based) structures. The interaction between these two views is not a simple combination, but a coordinated learning process that enhances structural consistency and representation robustness.

(3) Structure-aware contrastive learning objective.

We introduce a contrastive learning strategy tailored to heterogeneous biomedical networks, where positive and negative samples are constructed based on both topological proximity and semantic similarity. This differs from conventional contrastive learning schemes and is specifically designed to improve the discriminative power of herb–disease embeddings.

(4) Task-specific integration rather than generic stacking.

Importantly, our framework is not a straightforward stacking of existing modules. Each component is carefully adapted and jointly optimized for the herb–disease association prediction task, forming a unified learning objective that cannot be reduced to independent modules.

We will revise the manuscript to more clearly highlight these contributions and explicitly distinguish our method from existing hybrid frameworks.

**Comment** 2: The manuscript claims the construction of a new dataset named HData, yet the process of data collection, cleaning, integration, and validation is insufficiently described, particularly regarding how herb properties, chemical components, and disease associations were verified and how potential noise or inconsistent entries were handled.
**Response**: We would like to thank the reviewers for their valuable suggestions regarding the rigor of our dataset construction; we fully agree that the creation of a benchmark dataset requires a more detailed description. Regarding the issue of potential noise and inconsistent entries in the dataset collection process, we believe that, given the multi-component and multi-target nature of traditional Chinese medicines, fluctuations in component concentrations and variations in herbal materials from different origins may also lead to potential statistical bias. We therefore selected pairs of associations between traditional Chinese medicines and chemical molecules, or between traditional Chinese medicines and diseases, that are present in multiple databases or supported by literature. We then cross-validated these against independent databases to ensure the consistency of the core association data.

We have therefore included a detailed account of the data pre-processing steps in the manuscript (lines 78-110). This new section describes the sources from which the data was collected, as well as the standardisation of Chinese herbal medicine nomenclature, the verification and deduplication of chemical constituents, and the alignment of disease terminology during the construction of the dataset. To facilitate a more intuitive comparison with existing datasets, we have introduced a table comparing the functional attributes of various datasets. Existing datasets are primarily derived from a single database and contain only a small number of natural chemical constituents, with the remainder comprising synthetic small-molecule drugs; the drug data within these datasets consists almost exclusively of single-molecule drugs. In contrast, the Hdata dataset constructed in this paper takes “The Chinese Materia Medica” as its core reference, collecting herb-disease association data from multiple Chinese herbal medicine databases. It contains only natural herbal data, and the chemical constituents within the Chinese herbal medicines have undergone bioactivity screening and organization. Finally, researchers specialising in traditional Chinese medicine were invited to conduct manual spot-checks of the collected associations to ensure the accuracy of the dataset.
**Comment 3**:The dataset statistics appear questionable given that only a few hundred herbs and a limited number of diseases generate a very large number of confirmed herb–disease associations, which suggests an unusually dense relationship structure that may not reflect realistic biological knowledge and raises concerns about possible data leakage or artificially constructed associations.
**Response**: Unlike datasets such as Edata, which are constructed via random sampling over a full-spectrum distribution of herbs, the dataset used in this study is a high-confidence subset obtained through a filtering mechanism. The observed density is a direct consequence of our preprocessing procedure, in which isolated nodes were removed.

The resulting associations reflect the intrinsic multi-component nature of traditional Chinese medicine, where a single herb typically contains multiple active compounds that collectively act on specific diseases.

Each association in the Hdata dataset can be traced back to curated original literature and peer-reviewed biomedical databases. Therefore, although the dataset exhibits relatively high connectivity within a smaller set of nodes, this should not be interpreted as artificially constructed or spurious relationships, but rather as a reflection of biologically grounded interactions.

**Comment 4**: The procedure used to generate negative samples for the herb–disease association prediction task is not clearly explained, even though the learning objective assumes that all unobserved pairs are negative, which is a problematic assumption in biological interaction prediction where many unknown pairs may actually be undiscovered positives.

**Response**: We fully agree with the reviewer’s critical comment that unobserved herb–disease pairs cannot be simply treated as true negatives in biomedical interaction prediction, as many unknown pairs may correspond to undiscovered positive associations.

Given the current absence of experimentally validated gold-standard negative samples for herb–disease association prediction, we employ a dynamic negative sampling strategy to reduce the bias caused by fixed negative sampling. Specifically, inspired by the InfoNCE framework, we dynamically construct negative samples from other herb nodes within the same mini-batch during training. Since the batch composition changes iteratively, the negative set is continuously updated rather than fixed. This dynamic mechanism effectively alleviates the potential bias introduced by conventional static negative sampling.

We have revised the manuscript to provide a detailed and clear explanation of this negative sampling strategy (lines 399-407).

**Comment 5**: The description of similarity calculations for herbs and diseases lacks sufficient justification and validation, particularly the use of simple binary encoding of medicinal properties and the Jaccard similarity measure, which may oversimplify complex pharmacological relationships.
**Response**: The traditional pharmacological properties unique to traditional Chinese medicine are in fact highly generalized categorical characteristics. Using binary encoding combined with Jaccard similarity is a standard approach for measuring the similarity between discrete sets. This method can effectively preserve the ontological logic of traditional Chinese medicine in the model. Meanwhile, the hypergraph structure constructed from multiple chemical components corresponding to each herb, which we introduced in the model, functionally serves as a complement to herb similarity.

**Comment 6**: The construction of the heterogeneous adjacency matrix combining herb similarity, disease similarity, and association matrices is insufficiently explained, and the normalization procedure and its impact on graph structure and training stability are not analyzed.

**Response**: We sincerely thank the reviewer for this valuable suggestion. In the revised manuscript, we have provided a more detailed description of the construction and normalization strategy of the heterogeneous adjacency matrix (see the paragraph corresponding to Equation (4)).

Specifically, the heterogeneous adjacency matrix is constructed by integrating the herb similarity matrix, the disease similarity matrix, and the herb–disease association matrix. The upper-left block represents similarity relationships among herbs, while the lower-right block corresponds to similarity relationships among diseases. The off-diagonal blocks capture the associations between herbs and diseases, thereby forming a unified heterogeneous network structure.

In addition, we apply a normalization operation to the adjacency matrix during graph construction. This process is designed to alleviate the bias caused by highly connected nodes, ensuring a more balanced contribution of different nodes during information propagation and improving the stability of graph representation learning.

It is worth noting that weight adjustment in our model is performed at two stages: (1) normalization during the construction of the heterogeneous adjacency matrix, and (2) further weight assignment during the construction of higher-order structures, namely the hypergraph adjacency matrix.

**Comment 7**: The hypergraph modeling strategy based on herb–chemical relationships is conceptually interesting but lacks discussion regarding the biological validity of clique expansion and the assumption that shared chemical components directly imply functional similarity between herbs.

**Response**: We thank the reviewer for this insightful observation. Regarding the biological validity of group expansion and the potential limitation of the assumption that “shared components imply functional similarity,” we have carefully reflected on this issue and included additional discussion in the revised manuscript.

In our framework, each chemical component is modeled as a hyperedge connecting all herbs that contain this component. This design is grounded in the pharmacological principle that the therapeutic effects of herbs are primarily mediated by their bioactive chemical constituents. Therefore, herbs sharing the same chemical components may exhibit overlapping pharmacological activities or act on similar biological targets and pathways. Prior studies in natural product pharmacology and network pharmacology have shown that common active components often contribute to similar therapeutic effects across different herbs. From a network modeling perspective, hypergraphs provide a natural way to represent such many-to-many relationships. Unlike pairwise graphs that only capture binary interactions, hyperedges allow a single chemical component to simultaneously connect multiple herbs, thereby preserving the inherent higher-order structural information in herb–component associations.

Nevertheless, we acknowledge that the presence of shared chemical components does not necessarily guarantee identical therapeutic functions, as the pharmacological effects of herbs are also influenced by factors such as component concentration, synergistic interactions among compounds, and biological context. Therefore, in this study, the hypergraph representation is intended to capture potential functional similarity signals derived from shared components, rather than to assert strict functional equivalence.

To clarify this point, we have added a detailed discussion of the biological motivation and underlying assumptions of the hypergraph modeling strategy in Section 2.2.1 of the revised manuscript (lines 124-140).

**Comment** **8**: The model architecture description is fragmented and difficult to follow, as the roles of the graph encoder, hypergraph encoder, attention fusion module, and contrastive learning objectives are not clearly explained in a coherent pipeline.

**Response**: We have revised and reorganized the methodological description of our model and redesigned the overall framework diagram for better clarity. In the revised manuscript, a comprehensive overview of the entire model pipeline has been added in Section 2.3 to explicitly illustrate the data flow and interactions among different components (lines 367-380).

In brief, the proposed framework utilizes a graph encoder to capture structural information from the heterogeneous network, while a hypergraph encoder models higher-order relationships induced by shared chemical components among herbs. The representations learned from these two complementary views are then adaptively integrated through an attention-based fusion mechanism. In addition, a contrastive learning objective is introduced to further refine the representation space by enhancing consistency and discriminability. The final learned embeddings are subsequently used for herb–disease association prediction.

**Comment 9**: The attention based structural alignment module is insufficiently motivated because the manuscript does not demonstrate why this specific attention formulation is appropriate for aligning graph and hypergraph embeddings.

**Response**: We sincerely thank the reviewer for this valuable suggestion. The attention-based fusion module is designed to address the challenge that the graph encoder and the hypergraph encoder capture complementary structural information from different perspectives. Specifically, the graph encoder focuses on the topology of the heterogeneous network, while the hypergraph encoder models higher-order relationships among herbs induced by shared chemical components.

The relative importance of these two structural views may vary across different herbs, making fixed fusion strategies such as concatenation or simple averaging suboptimal. Therefore, we introduce an attention mechanism to adaptively learn node-specific weights for integrating the two embeddings. This enables the model to perform data-driven alignment between graph-based and hypergraph-based representations.

We have added a more detailed explanation of this module in the revised manuscript (lines 281-347).

**Comment** **10**: The paper does not provide sufficient information about hyperparameter tuning, sensitivity analysis, or the rationale behind choices such as embedding dimension, dropout rate, and learning rate, which raises concerns about reproducibility.

**Response**: We thank the reviewer for highlighting the importance of hyperparameter transparency and reproducibility. In the revised manuscript, we have provided a more detailed description of hyperparameter selection and sensitivity analysis (lines 605-620).

Specifically, the hyperparameters in our model are determined based on commonly adopted ranges in graph representation learning, combined with preliminary tuning experiments. We now explicitly report both the search space and the final selected values in the revised manuscript. The embedding dimension is selected from {64, 128, 256}, with 128 chosen as the final value. The learning rate is selected from {0.001, 0.005, 0.01}, with 0.01 adopted. For dropout, hyperdp is selected from {0.3, 0.5, 0.7} and dp from {0.3, 0.5}, with final values of 0.7 and 0.5, respectively. The similarity loss weight is chosen from {1, 3, 6, 10}, with 6 selected. The number of training epochs is set to 700 to ensure sufficient convergence.

In addition, we conducted sensitivity analyses on key hyperparameters, such as the embedding dimension and the similarity loss weight. The results show that the model performance remains relatively stable within reasonable ranges, demonstrating the robustness of the proposed method. To ensure reproducibility, all experiments are conducted with a fixed random seed (seed = 42).

Regarding input features, the herb feature dimension is 642, consisting of 321-dimensional chemical features and 321-dimensional similarity features, while the disease feature dimension is 1712, constructed based on disease semantic similarity. These feature dimensions are determined by the data sources rather than tuned as model hyperparameters.

**Comment 11**:The evaluation methodology relies exclusively on random five-fold cross validation, which is known to produce overly optimistic results in link prediction tasks when edges are randomly split instead of using more realistic evaluation strategies such as node level or cold start splits.
**Response**: We thank the reviewer for this valuable suggestion. We fully agree that random five-fold cross-validation in link prediction tasks may lead to overly optimistic evaluation results due to potential data leakage. To provide a more rigorous and realistic assessment, we have made the following improvements in the revised manuscript.

Specifically, we introduce additional experiments based on node-level splitting (lines 491-505). In this setting, nodes in the test set are entirely unseen during training, thereby simulating a more realistic “cold-start” scenario. Under the node-level split, we observe that changes in the graph topology make the original global negative sampling strategy result in extremely sparse samples. To better evaluate the model’s ability to discriminate potential links, we adopt a mini-batch-based negative sampling strategy during evaluation.

The experimental results show that, although the task becomes more challenging under this setting, our method still demonstrates strong robustness, particularly in terms of AUPR performance.

**Comment** **12**:- The comparative experiments with baseline methods are not sufficiently fair or transparent because there is no indication that the baseline models were properly tuned or implemented under identical experimental conditions.

**Response**: We thank the reviewer for the valuable comments regarding experimental fairness and transparency. We fully agree that all baseline methods should be evaluated under consistent and sufficiently optimized conditions to ensure a fair comparison.

In the revised manuscript, we have comprehensively refined and standardized the experimental settings to ensure that all models, including the proposed method and all baseline approaches, are evaluated within a unified framework. Specifically, all methods use the same training, validation, and test splits. They are provided with identical input information, including the same herb–disease association data and the same node features, without introducing any additional external information. Furthermore, a consistent random negative sampling strategy is adopted across all methods to construct training samples. The evaluation is conducted using the same set of metrics, including AUPR, Accuracy, Precision, Recall, and F1 Score.

In addition, we have supplemented the implementation details and hyperparameter settings of two baseline models in the revised manuscript to further improve transparency and reproducibility (lines 453-467). The updated experimental results demonstrate that, under these strictly controlled and unified conditions, the proposed method still outperforms all baseline models, thereby validating its effectiveness and robustness.

**Comment** **13**: The AUC values reported for some datasets are lower than competing methods while the paper still claims overall superiority, suggesting that the conclusions may selectively emphasize certain metrics while ignoring contradictory results.

**Response**: We thank the reviewer for this important comment. We acknowledge that, on certain datasets, our model does not achieve the best performance in terms of AUC when compared with some competing methods.

We would like to clarify that our claim regarding overall performance is based on a comprehensive evaluation across multiple datasets and metrics, with particular emphasis on AUPR, which is more appropriate for highly imbalanced link prediction tasks. Specifically, our model consistently demonstrates strong or competitive performance in terms of AUPR, which better reflects the ability to identify true positive associations under class imbalance. In contrast, while AUC measures overall ranking performance, it may be less sensitive to the positive class in highly imbalanced settings. Furthermore, across most datasets, our method exhibits stable and robust performance under multiple evaluation metrics.

In the revised manuscript, we will make the following changes to improve clarity and rigor. First, we will explicitly report and discuss cases where our method underperforms in terms of AUC. Second, we will avoid overly generalized claims such as “overall superiority” and instead adopt more precise and balanced descriptions.

We appreciate the reviewer’s suggestion, which helps us present a more balanced and rigorous analysis.

Reviewer #3:

This paper proposes GHF-ACL, a multi-order graph contrastive learning framework for herb–disease association prediction, which jointly models low-order functional similarities and high-order herb–chemical interactions through graph and hypergraph structures. It also introduces HData, a standardized benchmark dataset integrating herbal properties, chemical components, and disease associations, and uses an attention-guided interaction module together with hierarchical contrastive learning to align heterogeneous representations. Experimental results on one herb–disease dataset and several drug–disease benchmark datasets show that the proposed method achieves competitive or superior performance, particularly in AUPR, Recall, and F1 score, indicating its promise for imbalanced biomedical association prediction tasks.

To further strengthen the manuscript, it would be valuable to connect the proposed framework with more recent related studies and discuss how these advances could inform future methodological improvements.

**Comment** **1**: Several formulas in the Methods section lack complete variable definitions or clear derivations, which makes the method difficult to reproduce.

For example:

(1) In the disease semantic similarity formula (Eq. 3), the variable DV(d) is used but not explicitly defined before appearing in the equation.

(2) The degree matrices (D_r) and (\tilde{D}_d) in Eq. 4 are also not clearly explained.

(3) In the hypergraph formulation (A_{hyper} = HWH^T), the derivation of this adjacency representation (from hypergraph clique expansion or spectral hypergraph theory) is not sufficiently described.

**Response**: Thank you for your careful reading and insightful comments. We acknowledge that several equations in the Methods section previously lacked complete variable definitions and sufficient explanatory detail. In the revised manuscript, we have thoroughly addressed these issues and provided the necessary clarifications. Specifically (lines 234-240):

(1) We have introduced a clear definition of $D_{V}(d)$ prior to Eq. (3), where
$D_{V}(d)=\sum_{n\in N(d)} C_{d}(n)$denotes the total semantic contribution of disease $d$ within the disease DAG.

(2) We have systematically refined and standardized the notation throughout the manuscript. In particular, we explicitly define the degree matrices associated with the herb similarity matrix $S_{h}$and the disease similarity matrix $S_{d}$as:

$D_{h}(i,i)=\sum_{j} S_{h}(i,j)$,$D_{d}(i,i)=\sum_{j} S_{d}(i,j)$.

(3) In the original manuscript, the expression $A_{\text{hyper}}=HWH^{T}$was introduced to offer an intuitive understanding of the hypergraph structure. This formulation corresponds to a clique expansion perspective, in which herbs sharing common chemical components are interconnected with weights of $\frac{1}{d_{k}}$. It should be emphasized that this does not imply the direct construction of a hypergraph adjacency matrix. In practice, hypergraph convolution in our model is implemented via the normalized hypergraph Laplacian, as defined in the revised Eq. (7). This formulation follows the standard HyperGCN spectral convolution framework and explicitly captures higher-order relationships, rather than reducing the hypergraph to pairwise interactions. To avoid potential confusion, we have added detailed explanations in Section 2.2.1 to clearly distinguish between these concepts.

**Comment** **2**: The local contrastive loss function (Eq. 14) appears to contain a formulation error.

(1) The denominator of the loss currently uses the same similarity term as the numerator: $\sum_{k} \exp\left( \frac{\mathrm{sim}(z_{g},z_{h})}{\tau} \right)$

This formulation does not correctly represent contrastive learning because it does not compare the positive pair against negative samples.

(2) A correct InfoNCE-style formulation should include similarities with all other negative samples, e.g.: $\sum_{k} \exp\left( \frac{\mathrm{sim}(z_{g},z_{h})}{\tau} \right)$

**Response**: We would like to thank the reviewers for highlighting a key issue in the local contrast loss function. Upon careful examination, we have confirmed that the formulation of the denominator in the original manuscript was indeed somewhat imprecise, which may have led to an unclear expression of the contrastive learning objective.

(1) Regarding the issue with the denominator term:
We agree with the reviewer’s comment that, in the original formulation, the denominator incorrectly reused the similarity term of the positive pair, failing to properly reflect the core idea of contrasting positive and negative samples. In the revised manuscript (lines 399-407), we have corrected this formulation to follow the standard InfoNCE paradigm. Specifically, for each herb node $i$, we take its graph-view representation $z_{i}^{\left( g \right)}$as the anchor and its hypergraph-view representation $z_{i}^{\left( h \right)}$as the positive sample, while representations of other nodes are treated as negative samples. The formulation is defined as follows:

$$L_{con}=-\sum_{i} \log\frac{\exp(sim(z_{i}^{\left( g \right)},z_{i}^{\left( h \right)})/\tau)}{\exp(sim(z_{i}^{\left( g \right)},z_{i}^{\left( h \right)})/\tau)+\sum_{k\neq i} \exp(sim(z_{i}^{\left( g \right)},z_{k}^{\left( h \right)})/\tau)}$$

In particular, the denominator includes the current node’s positive samples as well as all negative samples, thereby enabling effective discrimination between positive and negative sample pairs.

(2) Regarding the construction of negative samples:
In implementation, we adopt a cross-view negative sampling strategy, where negative samples are drawn from the hypergraph-view representations of other nodes, i.e., $z_{k}^{\left( h \right)}$with $k\neq i$. During training, negative samples are dynamically sampled from the current mini-batch to approximate the full set of contrastive candidates. As the composition of mini-batches varies throughout training, the set of negative samples is continuously refreshed, which helps alleviate the bias that may arise from using a fixed negative set.

With this revised formulation, the model is able to effectively pull positive pairs closer in the embedding space while pushing negative samples farther apart, thereby facilitating robust alignment between representations across different structural views.

**Comment** **3**: The task is treated as a binary classification problem for herb–disease associations. However, the manuscript does not describe how negative samples are generated, which is crucial in link prediction tasks.

Important details missing include:

whether all unknown pairs are treated as negative samples

whether random negative sampling is used

whether the dataset is balanced or imbalanced during training

Since the model uses weighted binary cross-entropy, the computation of (w^+) and (w^-) should also be clearly explained.

Without this information, the experiment cannot be reproduced.

**Response**: Thank you for the reviewer’s valuable comments. We apologize for missing these critical implementation details, which are essential for reproducibility. In the revised manuscript, we have supplemented a clear and complete description of the negative sampling strategy and the weighted binary cross-entropy loss.

First, regarding negative sample generation:

We treat the task as binary classification for herb–disease associations. Not all unknown pairs are used as negative samples; instead, we perform random negative sampling during training. For each positive (herb–disease) pair, we randomly select a fixed number of negative pairs from unobserved associations to construct the training set. This strategy avoids extreme imbalance and reduces the risk of false negatives. The training set therefore remains naturally imbalanced rather than being artificially balanced.

Second, regarding the weighted binary cross-entropy loss:

w+ and w− denote the weights for positive and negative samples, respectively. They are computed based on the inverse ratio of the number of positive and negative samples in the training set, which alleviates the bias caused by class imbalance and stabilizes model optimization.

All these details have been added to the revised manuscript to ensure full reproducibility (lines 281-332).

**Comment** **4**: Given that the proposed method is based on contrastive graph learning and hypergraph modeling, it should be compared with more recent frameworks such as: recent hypergraph neural networks (i.e., 10.1093/bib/bbaf399), graph contrastive learning models, transformer-based graph models for biomedical networks.

**Response**: We thank the reviewer for the constructive suggestion regarding baseline comparison. We greatly appreciate the recommendation of recent advances in hypergraph learning and graph contrastive learning for biomedical networks.

In the revised manuscript, we have strengthened the comparison by adding two recent, representative, and highly competitive state-of-the-art models:

(1) GeNNius, a powerful graph neural network-based method for biomedical interaction prediction;

(2) HDCTI, a state-of-the-art hypergraph learning framework for modeling high-order complex relations in biomedical data.

These newly added baselines cover the mainstream advanced techniques in hypergraph modeling and graph representation learning, consistent with the research direction suggested by the reviewer. All models are evaluated under the same unified experimental settings to ensure fairness.

The updated results demonstrate that our method still outperforms these recent competitive baselines, validating the effectiveness of our approach (lines 491-506).

**Comment** **5:** The case study presents predicted herb–disease associations such as: Niuxi → Skin Neoplasms. However, the biological evidence provided is mostly indirect literature interpretation, rather than strong validation. To better support the practical value of the model, the authors should consider: validating top predictions using external biomedical databases; performing pathway enrichment analysis; verifying predicted associations using recent pharmacological studies.

**Response**: Thank you for your insightful comment. We agree that relying solely on indirect evidence from literature is insufficient to fully validate the predicted associations between herbs and diseases. We would like to clarify that our model is designed to directly predict herb–disease associations, without explicitly modeling intermediate biological entities such as targets. Therefore, instead of adopting target-based validation strategies, we employ a post hoc biological validation approach to support the predicted associations. Specifically, we have strengthened the case study from the following aspects:

(1) Structural analysis:
We further analyzed the embedding space and found that Niuxi is located near several herbs known to be associated with skin-related diseases or antitumor effects. Notably, these herbs do not exhibit strong similarity with Niuxi in the first-order graph, nor do they share identical chemical components. Instead, they demonstrate consistent pharmacological properties (e.g., promoting blood circulation), suggesting that the model captures higher-order functional patterns rather than relying on direct similarity. From a hypergraph perspective, Niuxi is closer to herbs such as Yuanhua, Haifeng Teng, and Xinyi in the high-order embedding space compared to the first-order relational space. Previous studies have reported that these herbs exhibit therapeutic effects on skin-related diseases or tumor-associated pathological processes, providing additional support for the predicted association.

(2) Literature evidence:
We further examined existing biomedical databases and recent studies. Multiple reports have demonstrated the antitumor and anti-inflammatory properties of these herbs, which are closely related to tumor progression. These findings provide indirect yet meaningful biological support for the predicted results.

It is also worth emphasizing that none of the top 15 predicted associations appear in the training dataset, which further demonstrates the model’s ability to uncover novel herb–disease associations beyond observed data (lines 651-658).

Minor Revision Comments

**Comment** **1**: English grammar and wording need improvement. Several sentences contain minor grammatical or stylistic issues. Examples include:incorrect phrasing such as “denote to”; unnecessary adverbs such as “Simultaneously”; inconsistent use of singular/plural forms.

**Response**: We appreciate the reviewer’s comment regarding language clarity. The manuscript has been thoroughly proofread, and all identified grammatical and stylistic issues—including incorrect phrasing, unnecessary adverbs, and singular/plural inconsistencies—have been corrected.

**Comment** **2**: Figure captions and diagrams require clearer explanations. Some figures are insufficiently described. For example: Figure 1 (model architecture) includes several modules but does not clearly explain the data flow between them; The hypergraph construction process could be illustrated more clearly.

**Response**: Thank you for your valuable suggestion. We agree that the original presentation of Figure 1 lacked sufficient clarity in terms of data flow and module interactions. In the revised manuscript, we have substantially improved both the figure and its accompanying description as follows:

(1) Refined visualization of the model architecture:
We have redesigned Figure 1 to provide a clearer and more intuitive illustration of the data flow across modules, including the herb feature encoder, disease feature encoder, hypergraph construction module, contrastive learning alignment, attention-based fusion, and the final prediction component. Arrows and module boundaries are now explicitly labeled to clearly depict the flow of information throughout the model.

(2) Step-by-step procedural description:
We have added a detailed, stepwise explanation describing how the input data—namely herb features, disease features, association matrices, and hypergraph structures—are processed and transformed at each stage of the model.

The overall workflow of the proposed model can be summarized in a dimension-consistent manner as follows (lines 368-380):

Step 1: Input construction.
The model takes two types of inputs: a standard feature matrix of size (2033,2033) and a hypergraph-based feature matrix of size (2033,7279).

Step 2: GCN branch.
The standard feature matrix (2033,2033) is projected to (2033,128) through multiple graph convolution layers. The outputs of different layers are aggregated to obtain the final GCN embedding (2033,128).

Step 3: Hypergraph branch.
The hypergraph feature matrix (2033,7279) is first transformed into (2033,128), followed by hypergraph convolution, producing the hypergraph embedding (2033,128).

Step 4: Cross-view alignment and fusion.
The two embeddings (2033,128) and (2033,128) are used to compute a similarity matrix of size (2033,2033) for contrastive learning. After alignment, the embeddings are fused into a unified representation of size (2033,128).

Step 5: Prediction layer.
The fused representation (2033,128) is divided into herb embeddings (321,128) and disease embeddings (1712,128), which are used to generate a prediction matrix of size (321,1712).

(3) Clarification of hypergraph construction:
We have further elaborated on the hypergraph construction process. Specifically, each hyperedge corresponds to a set of herbs sharing common molecular components. The incidence matrix is constructed based on herb–component relationships, and the hyperedge weights are defined as the inverse frequency of the corresponding components.

These revisions significantly enhance the clarity, readability, and interpretability of the proposed model architecture.

**Comment** **3**: Hyperparameter settings lack justification. The manuscript reports the use of parameters such as: embedding dimension = 128; learning rate = 0.0001; dropout rates = 0.7 and 0.5; training epochs = 5000. However, the rationale for these choices is not discussed.

**Response**: Thank you for highlighting the importance of hyperparameter transparency and reproducibility. We have revised the manuscript to provide a more detailed description of hyperparameter selection and sensitivity analysis (lines 604-620).

Specifically, the main hyperparameters were determined based on commonly adopted ranges in graph representation learning, combined with preliminary tuning experiments. In the revised manuscript, we explicitly report both the search space and the final selected values: the embedding dimension is chosen from {64, 128, 256}, with 128 as the final setting; the learning rate is selected from {0.001,0.005,0.01}, with 0.01 adopted; the dropout rates are set as $\text{hyperdp}\in\{0.3, 0.5, 0.7\}$ and $\text{dp}\in\{0.3, 0.5\}$, with final values of 0.7 and 0.5, respectively; the similarity loss weight is chosen from {1, 3, 6, 10}, with 6 selected; and the number of training epochs is set to 700 to ensure sufficient convergence.

In addition, we conducted sensitivity analyses on key hyperparameters, such as the embedding dimension and the similarity loss weight. The results indicate that model performance remains relatively stable within reasonable ranges, demonstrating the robustness of the proposed method. To further ensure reproducibility, all experiments were conducted with a fixed random seed (seed=42).

**Comment** **4:** Dataset construction details should be clarified. The manuscript introduces a new dataset HData, which integrates herbs, diseases, and chemical components. However, some dataset preparation steps remain unclear: how duplicate herb names were resolved; how chemical component identifiers were standardized; how missing data were handled.

**Response**: We fully agree that the construction of a benchmark dataset requires a more comprehensive and transparent description. In the revised manuscript, we have added a dedicated section detailing the data preprocessing procedures (lines 78-96).

This new section elaborates on the data sources and the key steps involved in dataset construction, including the standardization of herb nomenclature, validation and deduplication of chemical components, and alignment of disease terminologies. To facilitate a clearer comparison with existing datasets, we have also introduced a comparative table summarizing key characteristics and functionalities across multiple datasets.

Most existing datasets are derived from a single database and typically contain only a limited number of natural compounds, with the majority being synthetic small-molecule drugs. Moreover, the drug-related data in these datasets predominantly correspond to single-molecule entities. In contrast, the proposed Hdata dataset is constructed with *Chinese Materia Medica* as the core reference and integrates herb–disease association data from multiple traditional Chinese medicine databases. It exclusively includes natural herbal data and further incorporates curated and activity-filtered chemical components derived from herbs.

These enhancements significantly improve the transparency, completeness, and scientific value of the constructed dataset.

**Comment** **5**: Some evaluation metrics are redundant or insufficiently discussed. The paper reports several metrics, including: Accuracy, Precision, Recall, F1 score, AUC, AUPR. However, in highly imbalanced link prediction tasks, AUPR is usually more informative than accuracy.

**Response**: Thank you for this insightful comment. We fully agree that, in highly imbalanced link prediction tasks, the Area Under the Precision–Recall Curve (AUPR) is often more informative than accuracy, as it better reflects model performance on the positive class.

In our study, we employ a comprehensive set of evaluation metrics, including accuracy, precision, recall, F1-score, AUC, and AUPR, to provide a holistic assessment of model performance. In particular, precision, recall, and F1-score help elucidate the trade-off between false positives and false negatives.

In the revised manuscript, we have made the following improvements:
(1) We explicitly emphasize AUPR as the primary evaluation metric;
(2) We reduce redundancy by streamlining the presentation of secondary metrics;
(3) We provide a more detailed discussion on metric selection and its relevance to imbalanced link prediction tasks.

We sincerely appreciate the reviewer’s suggestion, which has helped improve the clarity and rigor of our evaluation framework.

**Comment** **6**: The authors may consider incorporating insights from recent studies such as 10.1016/j.csbj.2024.06.032, 10.1093/bib/bbac384, 10.1109/TCBBIO.2025.3610881, 10.1002/advs.202512453, and 10.1109/JBHI.2024.3383591, which present advanced graph-based or biomedical representation learning approaches for association prediction tasks. Integrating the methodologies or comparative analyses from these works in future research could further enhance the robustness, generalization ability, and biomedical interpretability of the proposed GHF-ACL framework.

**Response**: Thank you for your valuable and insightful suggestion. We fully agree that recent advances in graph-based biomedical representation learning provide important guidance for association prediction tasks. These studies highlight several key directions, including heterogeneous information network modeling, structure-aware representation learning, and geometric deep learning frameworks for integrating biomedical data, all of which have demonstrated strong capabilities in capturing complex biological relationships.

Our proposed GHF-ACL framework aligns well with these emerging trends by integrating heterogeneous structural information and multi-order representation learning. In particular, the combination of a graph encoder and a hypergraph encoder enables the model to simultaneously capture pairwise relationships and higher-order interactions.

We also acknowledge that further improvements could be achieved by incorporating more advanced strategies inspired by these studies, such as geometric or spatial representation learning to better model underlying biological structures. In the revised manuscript, we will include a more detailed discussion of these research directions to better position our work within the context of recent developments (lines 647-685).

We sincerely appreciate the reviewer’s suggestion, which helps enhance both the relevance of our framework and its potential for future extensions.
