# Supplementary material for: GHF-ACL: A novel contrastive learning framework with multi-order graph structures for herb-disease association prediction
Source: PLoS Comput Biol. 2026 Jun 29;22(6):e1014461. doi: 10.1371/journal.pcbi.1014461 (PMC13327522; doi:10.1371/journal.pcbi.1014461)
Supplement: Attachment — Submitted filename: Response_to_Reviewers_auresp_2.docx [file pcbi.1014461.s003.docx]

Dear Editor and Reviewers,

We sincerely thank you and the reviewers for the careful evaluation of our revised manuscript and for the constructive comments. We are grateful that the reviewers recognized the improvements made in the previous revision, including the expanded methodological explanations, clarified mathematical notation, enhanced dataset description, stronger baseline comparisons, and additional node-level evaluation.

In this revision, we have carefully addressed the remaining concerns raised by Reviewer #2. Specifically, we further clarified the methodological contribution of GHF-ACL, provided a more transparent description of the data construction process, discussed the potential influence of preprocessing on graph connectivity and task difficulty, distinguished the negative sampling strategies used for supervised learning, contrastive learning, and evaluation, refined the description of the adaptive gating-based fusion module, expanded the node-level evaluation of baseline models, and moderated the claims regarding model superiority across evaluation metrics.

We hope that the revised manuscript and the accompanying responses adequately address the reviewers’ concerns. We sincerely appreciate the editor’s and reviewers’ time and valuable suggestions.

Sincerely,
The Authors

**Reviewer #1:**

We sincerely thank the reviewer for the careful evaluation of our revised manuscript and for the positive recognition of the improvements made in this version. We greatly appreciate the reviewer’s constructive comments in the previous round, which helped us improve the clarity, rigor, and presentation of the manuscript.

**Reviewer #2:**

We sincerely thank the reviewer for the careful evaluation of our revised manuscript and for acknowledging the improvements made in the previous revision. We are grateful for the constructive comments, which have helped us further clarify the methodological contribution, data construction process, evaluation protocol, and limitations of the proposed framework. We have carefully considered each concern and revised the manuscript accordingly. Detailed responses are provided below.

**Comment 1**: The revised manuscript explains that the contribution lies in the integration of heterogeneous graph learning, hypergraph modeling, attention-based fusion, and contrastive learning for herb–disease association prediction. However, the explanation still largely frames the method as a task-specific combination of existing components rather than clearly establishing a fundamentally new methodological contribution. The authors should more explicitly distinguish their technical contribution from prior graph contrastive learning, hypergraph learning, and biomedical association prediction models, ideally by explaining which part of the architecture or learning objective is not directly inherited from existing frameworks.

**Response**:

We sincerely thank the reviewer for this insightful comment. We agree that the previous manuscript did not sufficiently distinguish the methodological contribution of GHF-ACL from a straightforward combination of existing graph learning components. In the revised manuscript, we have strengthened the discussion of novelty in both the Introduction and Section 2.2.

We would like to clarify that the contribution of GHF-ACL does not lie in proposing entirely new graph operators, but in the development of a problem-driven framework specifically designed for herb–disease association prediction, where three mutually dependent components are introduced to address complementary limitations of existing approaches.

First, unlike conventional graph-based methods that model only pairwise herb–disease interactions, GHF-ACL explicitly captures both pairwise therapeutic relationships and higher-order herb–component sharing patterns through a dual-view graph–hypergraph architecture. The higher-order structural information carried by shared chemical components cannot be adequately represented in ordinary graphs without information loss.

Second, the proposed hypergraph construction is tailored to the characteristics of Traditional Chinese Medicine data. To reduce the influence of ubiquitous chemical components that contribute limited discriminative information, we introduce an inverse-degree weighting strategy during hypergraph construction. Consequently, rare and functionally specific components contribute more strongly to herb similarity than widely shared components. This design is motivated by the compositional characteristics of herbal medicines and differs from standard hypergraph formulations that treat hyperedges uniformly.

Third, the graph and hypergraph encoders generate representations from fundamentally different structural perspectives. Instead of directly fusing these embeddings, we introduce a cross-view contrastive alignment mechanism to encourage consistency between pairwise therapeutic semantics and higher-order chemical semantics before downstream prediction. In contrast to conventional graph contrastive learning frameworks, which typically operate on augmented views of the same graph, our contrastive objective is designed to reconcile heterogeneous structural views derived from different biological relationships.

Finally, we employ an adaptive node-level gating mechanism to dynamically balance information from the graph and hypergraph representations. This module allows the model to selectively integrate complementary information from both structural views while reducing the influence of potentially inconsistent features. Additional ablation experiments further demonstrate that this adaptive fusion strategy consistently outperforms simpler alternatives such as direct concatenation.

Taken together, the novelty of GHF-ACL lies not in any single component individually, but in the unified framework that jointly models pairwise therapeutic associations, higher-order chemical relationships, cross-view semantic consistency, and adaptive structural fusion. We have revised the manuscript accordingly to make these methodological distinctions and design motivations more explicit. (Lines 59-64 and lines 141-143)

**Comment 2**: The authors now describe standardization, deduplication, disease-term alignment, bioactivity screening, and manual spot-checking, but the manuscript still does not provide enough quantitative evidence about the reliability of the associations. Given the unusually dense herb–disease relationship structure, the authors should provide clearer statistics on the original number of records, removed duplicates, excluded noisy entries, retained associations, and the proportion of associations supported by multiple databases or literature sources. Without this, it remains difficult to judge whether the high density reflects real biological knowledge or possible construction bias.

**Response**:

We thank the reviewer for this important request and apologize for the insufficient quantitative description in the previous version. We provide the following detailed statistics on the data construction process.

For herb data, a total of 19,595 molecule–herb records were initially collected from TCMSP and PubChem. After applying ADME-based filtering criteria (oral bioavailability OB ≥ 30% and drug-likeness DL ≥ 0.18), 7,279 unique chemical compounds were retained, corresponding to a retention rate of approximately 37.1%. Herbs for which the number of retained active compounds fell below a predefined confidence threshold were excluded, reducing the herb set from 499 candidate entries to the final 321 herbs included in HData.

For herb–disease associations, a total of 124,583 raw ingredient–disease association records were collected from TCM-ID, NPACT, the Chinese TCM Database, and NMPA. After standardizing disease nomenclature using MeSH IDs, deduplicating redundant entries, and aligning herb names to the authoritative Chinese Materia Medica standard, 65,536 herb–disease associations were retained across 321 herbs and 1,712 diseases, representing a retention rate of approximately 52.6%. The relatively high density of the resulting dataset (sparsity 11.93%) compared to conventional drug–disease datasets reflects the multi-target, multi-component nature of herbal medicines, in which each herb is typically associated with a broad spectrum of therapeutic indications documented across multiple independent sources.

We acknowledge that the high association density warrants transparency. The retained associations are supported by records from at least one authoritative database, and the multi-source integration strategy — combining TCM-ID, NPACT, the Chinese TCM Database, and NMPA — increases the likelihood that retained associations reflect genuine biological relationships rather than single-source annotation artifacts. We have added these statistics to Section 2.1 of the revised manuscript. (Lines 100-110)

**Comment 3:** The authors state that isolated nodes were removed and that HData is a high-confidence subset, but this explanation does not fully resolve the concern that filtering may artificially increase connectivity and make the prediction task easier. The authors should report how performance changes before and after removing isolated or weakly connected nodes, or at least discuss how this preprocessing choice affects task difficulty and model evaluation.

**Response**：We thank the reviewer for this valuable observation.

We agree that removing isolated nodes may increase graph connectivity and potentially reduce task difficulty to some extent. However, isolated nodes do not participate in any herb–disease associations and therefore contain no relational information that can be exploited by graph-based representation learning methods. Since both the graph encoder and hypergraph encoder in GHF-ACL rely on neighborhood aggregation and message passing mechanisms, meaningful representations cannot be effectively learned for completely isolated nodes.

The purpose of removing isolated nodes was therefore not to improve predictive performance, but to ensure that all retained nodes contain at least minimal structural information required for graph learning. This preprocessing strategy is commonly adopted in graph representation learning studies, particularly when evaluating models based on node interactions and topological information.

Nevertheless, we acknowledge that filtering isolated nodes may slightly increase overall graph connectivity and therefore reduce the difficulty of the prediction task. To improve transparency, we have added an explicit discussion of this issue in the revised manuscript. Future work will investigate more challenging settings involving sparsely connected and isolated nodes. (Lines 111-115)

**Comment 4:** The negative sampling strategy remains confusing and somewhat inconsistent across the manuscript and response. In one part, the authors describe random negative sampling from unobserved herb–disease pairs at a 1:10 positive-to-negative ratio, while in the contrastive learning section they describe dynamic mini-batch negative sampling using other herb-node representations. These are different forms of negative sampling for different objectives, but the manuscript should separate them much more clearly. The authors should explicitly define negative samples for the supervised link-prediction loss, negative samples for the contrastive loss, and negative samples used during evaluation. They should also discuss the risk that randomly selected unobserved herb–disease pairs may include unknown positives.
**Response：**

We sincerely apologize for the confusion caused by the ambiguous description in the previous version. We fully agree with the reviewer’s constructive suggestion. To address this issue, we have thoroughly revised the manuscript and formally distinguished, defined, and standardized the different negative sampling mechanisms adopted at different stages of the framework:

We clarified two independent negative sampling schemes used for training and evaluation, respectively:

1. For supervised training (link prediction loss)
   During training, we randomly sampled unobserved herb–disease pairs as negative samples at a positive-to-negative ratio of 1:10. This moderate sampling ratio ensures training stability while reducing the bias caused by severe class imbalance.
2. For contrastive learning (alignment loss)
   Following the standard InfoNCE framework, negative samples are dynamically constructed within each mini-batch using the representations of other herb nodes. These negative samples are independent of herb–disease association labels and are used only for cross-view representation alignment.
3. Model evaluation stage (testing stage)
   During evaluation, all unobserved herb–disease pairs in the test set are directly regarded as negative samples, without additional sampling. This setting reflects the data imbalance in real-world scenarios and enables fair computation of global ranking metrics, including AUROC and AUPR.

To ensure rigorous evaluation, we adopted two splitting schemes:

Standard five-fold cross-validation (edge-level split): used to evaluate the overall link prediction capability.

Node-level (cold-start) split: used to test the generalization ability for unseen herbs/diseases, which represents a more realistic and more challenging scenario.

Under both schemes, the negative samples used for evaluation remain consistent: all unobserved test pairs are used.

Regarding the risk of unknown positive samples, we acknowledge that, because the herb–disease network is inherently incomplete, randomly selected unobserved pairs may contain true but unverified therapeutic associations. In our framework, the cross-view contrastive learning module can mitigate this risk. By optimizing global and local semantic clustering in the representation space, the contrastive objective ensures that semantically similar herbs are placed closer to each other, thereby preventing potential hidden positive samples from being overly penalized solely by the supervised link prediction loss.

We have accordingly updated Section 3.2 of the experimental settings and the Discussion section to ensure mathematical rigor and consistency. (Lines 434-443)

**Comment 5:** The authors justify binary encoding and Jaccard similarity as appropriate for categorical TCM properties, but this remains a very simple representation of complex pharmacological relationships. The authors should either provide empirical validation showing that these similarity measures correlate with known functional or therapeutic categories, or perform a sensitivity analysis using alternative similarity definitions. Otherwise, the choice remains insufficiently justified.
**Response：**

We thank the reviewer for raising this important methodological question. We would like to clarify that the choice of binary encoding combined with Jaccard similarity is not arbitrary, but is specifically motivated by the nature of TCM pharmacological data, and is consistent with established practices in the field.

First, the pharmacological properties of Chinese herbal medicines — including the Four Natures, Five Flavors, Meridian Tropism, and Toxicity — are inherently discrete categorical attributes. These properties do not possess a natural numerical ordering or continuous magnitude; for instance, there is no meaningful quantitative distance between "pungent" and "bitter" flavors, or between "heart" and "lung" meridian tropism. Binary encoding is therefore the most appropriate representation, as it faithfully preserves the categorical nature of these attributes without introducing spurious ordinal relationships that continuous-valued encodings would impose.

Second, Jaccard similarity is particularly well-suited for sparse binary feature vectors of this type. Since each herb typically possesses only a small subset of all possible attributes, the resulting feature vectors are highly sparse. In such settings, Jaccard similarity — which measures the ratio of shared attributes to the union of all attributes — is more robust than alternatives such as cosine similarity, which can be disproportionately influenced by the large number of shared zero entries in sparse vectors.

Third, this encoding and similarity strategy is consistent with established practices in computational TCM research. Prior works including HGHDA and HTInet, which represent the closest related methods in this domain, adopt equivalent or closely analogous attribute encoding strategies for herbal property representation. The adoption of Jaccard similarity for binary TCM property vectors therefore reflects a field-wide consensus rather than an ad hoc design choice.

For these reasons, we believe that binary encoding combined with Jaccard similarity constitutes a well-justified and domain-appropriate choice for quantifying herb similarity based on pharmacological properties. We have added a more explicit justification of this design choice in Section 2.2.1 of the revised manuscript. (Lines 171-176)

**Comment 6:** The attention-based structural alignment module is now more dimensionally consistent, but the motivation remains limited. The revised formulation appears to be a node-level gating mechanism between graph and hypergraph embeddings rather than a true attention or cross-attention mechanism. This is acceptable if clearly stated, but the manuscript should avoid overstating it as semantic alignment unless additional evidence is provided. The authors should compare this gating mechanism against simpler fusion strategies such as concatenation, averaging, and fixed weighted fusion to demonstrate that the proposed module is actually necessary.

**Response:** We thank the reviewer for this precise and constructive comment.

We agree that the proposed module is more accurately characterized as a node-level scalar gating mechanism rather than a full attention or cross-attention mechanism. Accordingly, we have revised the manuscript to avoid overstating its role as a semantic alignment module and now describe it as an adaptive gating-based fusion strategy.

Regarding comparisons with alternative fusion methods, our ablation study already includes a direct comparison between the proposed gating mechanism and simple concatenation (the “w/o Structural Interaction Alignment” variant in Table 9), which represents one of the most widely used fusion strategies in graph representation learning. The results show that replacing the adaptive gating mechanism with direct concatenation leads to a noticeable degradation in predictive performance, indicating that adaptive balancing of graph-view and hypergraph-view representations is beneficial for association prediction.

We acknowledge that comparisons with averaging and fixed-weight fusion could provide additional evidence. However, concatenation preserves information from both views without imposing predefined weighting assumptions and is therefore commonly adopted as a strong baseline for representation fusion. The observed performance gap between concatenation and the proposed gating mechanism suggests that adaptive node-level weighting provides additional benefits beyond simple feature aggregation.

Following the reviewer’s suggestion, we have revised Sections 2.2.3 and 3.4 to explicitly describe this component as a lightweight scalar gating mechanism and removed statements that could be interpreted as implying a full attention or cross-attention architecture.

**Comment 7:** The manuscript continues to rely heavily on random five-fold cross-validation, which may overestimate performance in link prediction. The new node-level split is useful, but the authors should report the full results clearly and compare all major baselines under the same node-level or cold-start setting. It is not sufficient to state that the proposed method remains robust unless the same stricter evaluation is applied transparently to competing methods.
**Response**:

We sincerely appreciate the reviewer’s critical and constructive comments. We have re-run all major baseline models under the same node-level (cold-start) evaluation protocol as GHF-ACL.

We have expanded Table 7 to include complete performance results of all competing methods under the node-level split. The updated results clearly show that our GHF-ACL model still achieves significant and consistent improvements over all baselines in AUPR, Recall, and F1-score, which are the most reliable metrics for imbalanced biomedical data, further validating the effectiveness and robustness of our model in real-world cold-start scenarios.

We have also revised the relevant descriptions in the manuscript to unify the evaluation protocol and avoid misleading statements about negative sampling during the evaluation phase. All models are evaluated under completely consistent settings to ensure fairness and reproducibility. (Lines 562-563)

**Comment 8:** The discussion of AUC versus AUPR is more balanced than before, but the manuscript still needs to avoid overly broad claims of superiority. In some datasets, the proposed method has lower AUC than competing methods, while the abstract and results emphasize AUPR, recall, or F1 improvements. The authors should consistently state that the method is strongest mainly in AUPR and recall-oriented evaluation under imbalance, rather than claiming general superiority across metrics.

**Response：**

We thank the reviewer for this precise observation and fully agree that the performance claims in the abstract and results section should be more carefully qualified. We acknowledge that GHF-ACL does not achieve the highest AUC across all datasets; specifically, DD-HGNN+ obtains higher AUC values on Cdata and Ldata. We have revised the abstract, results section, and conclusion to consistently reflect that the primary advantage of GHF-ACL lies in AUPR, Recall, and F1 Score — metrics that are more reliable indicators of model performance under severe class imbalance — rather than claiming general superiority across all metrics. (Lines 505-508 and lines 728-731)
