# Supplementary material for: GHF-ACL: A novel contrastive learning framework with multi-order graph structures for herb-disease association prediction
Source: PLoS Comput Biol. 2026 Jun 29;22(6):e1014461. doi: 10.1371/journal.pcbi.1014461 (PMC13327522; doi:10.1371/journal.pcbi.1014461)
Supplement: Peer review history

# Peer review: Decision Letter 0

**DOI:** 10.1371/journal.pcbi.1014461.r001

**Authors:** Mark Alber, Lun Hu

11 Mar 2026

-->PCOMPBIOL-D-26-00332

GHF-ACL: A Novel Contrastive Learning Framework with Multi-order Graph Structures for Herb-Disease Association Prediction

PLOS Computational Biology

Dear Dr. Zhang,

Thank you for submitting your manuscript to PLOS Computational Biology. After careful consideration, we feel that it has merit but does not fully meet PLOS Computational Biology's publication criteria as it currently stands. Therefore, we invite you to submit a revised version of the manuscript that addresses the points raised during the review process.

Please submit your revised manuscript by May 11 2026 11:59PM. If you will need more time than this to complete your revisions, please reply to this message or contact the journal office at ploscompbiol@plos.org. When you're ready to submit your revision, log on to https://www.editorialmanager.com/pcompbiol/ and select the 'Submissions Needing Revision' folder to locate your manuscript file.

Please include the following items when submitting your revised manuscript:

* A letter that responds to each point raised by the editor and reviewer(s). You should upload this letter as a separate file labeled 'Response to Reviewers'. This file does not need to include responses to formatting updates and technical items listed in the 'Journal Requirements' section below.

* A marked-up copy of your manuscript that highlights changes made to the original version. You should upload this as a separate file labeled 'Revised Manuscript with Track Changes'.

* An unmarked version of your revised paper without tracked changes. You should upload this as a separate file labeled 'Manuscript'.

If you would like to make changes to your financial disclosure, competing interests statement, or data availability statement, please make these updates within the submission form at the time of resubmission. Guidelines for resubmitting your figure files are available below the reviewer comments at the end of this letter

We look forward to receiving your revised manuscript.

Kind regards,

Lun Hu

Academic Editor

PLOS Computational Biology

Mark Alber

Section Editor

PLOS Computational Biology

**Additional Editor Comments:**

I received three review reports and all reviewers found merit in this work. However, they also raised several critical concerns to improve the quality of this work, such as the justification of novelty, the details of experiment setup, and the extension of validation. Due to these issues, I would like to ask authors to revise their manuscript for major revision.

**Journal Requirements:**

1) Please ensure that the CRediT author contributions listed for every co-author are completed accurately and in full.

At this stage, the following Authors/Authors require contributions: yunmeng Zhang, Meiling Liu, Qiutong Wang, Lin Shi, and Guohua Wang. Please ensure that the full contributions of each author are acknowledged in the "Add/Edit/Remove Authors" section of our submission form.

The list of CRediT author contributions may be found here: https://journals.plos.org/ploscompbiol/s/authorship#loc-author-contributions

2) We ask that a manuscript source file is provided at Revision. Please upload your manuscript file as a .doc, .docx, .rtf or .tex. If you are providing a .tex file, please upload it under the item type u2018LaTeX Source Fileu2019 and leave your .pdf version as the item type u2018Manuscriptu2019.

3) Please upload all main figures as separate Figure files in .tif or .eps format. For more information about how to convert and format your figure files please see our guidelines:

https://journals.plos.org/ploscompbiol/s/figures

4) Some material included in your submission may be copyrighted. According to PLOSu2019s copyright policy, authors who use figures or other material (e.g., graphics, clipart, maps) from another author or copyright holder must demonstrate or obtain permission to publish this material under the Creative Commons Attribution 4.0 International (CC BY 4.0) License used by PLOS journals. Please closely review the details of PLOSu2019s copyright requirements here: PLOS Licenses and Copyright. If you need to request permissions from a copyright holder, you may use PLOS's Copyright Content Permission form.

Please respond directly to this email and provide any known details concerning your material's license terms and permissions required for reuse, even if you have not yet obtained copyright permissions or are unsure of your material's copyright compatibility. Once you have responded and addressed all other outstanding technical requirements, you may resubmit your manuscript within Editorial Manager.

Potential Copyright Issues:

- Figure 1: Please confirm whether you drew the images / clip-art within the figure panels by hand. If you did not draw the images, please provide (a) a link to the source of the images or icons and their license / terms of use; or (b) written permission from the copyright holder to publish the images or icons under our CC BY 4.0 license. Alternatively, you may replace the images with open source alternatives. See these open source resources you may use to replace images / clip-art:

- https://commons.wikimedia.org

- https://openclipart.org/.

5) Please amend your detailed Financial Disclosure statement. This is published with the article. It must therefore be completed in full sentences and contain the exact wording you wish to be published.

State the initials, alongside each funding source, of each author to receive each grant. For example: "This work was supported by the National Institutes of Health (####### to AM; ###### to CJ) and the National Science Foundation (###### to AM).".

If you did not receive any funding for this study, please simply state: u201cThe authors received no specific funding for this work.u201d

**Reviewers' comments:**

Reviewer's Responses to Questions

**Comments to the Authors:**

**Please note here if the review is uploaded as an attachment.**

Reviewer #1: 1. the primary contribution of this work is framed around the introduction of a new dataset, hdata, yet the manuscript fails to provide a rigorous justification for its necessity or a comparative analysis against existing resources beyond basic statistical sparsity metrics reported in table 2 you must substantially expand the dataset description to include the sources and preprocessing steps for each data type (herbal properties, chemical compositions, and disease associations) with far greater detail additionally a direct quantitative and qualitative comparison against the existing datasets used in the baselines (e.g., lrsl, cdata, edata) is required to demonstrate that hdata offers unique challenges or information not already captured this is not merely a matter of curation but of establishing a new benchmark standard you need to clearly articulate what specific limitations in existing data hdata overcomes and ideally provide a side-by-side feature comparison table to justify its creation and adoption by the community

2. the core methodological novelty of your multi-order graph structure is undermined by a lack of clarity and, in some cases, what appears to be mathematical inconsistency in its formulation when you define the unified adjacency matrix a_h in equation 4 you combine normalized similarity matrices with the association matrix a however the notation for the disease side uses ~d^{-1/2}_d s_d ~d^{-1/2}_d without defining ~d_d or its relationship to the previously defined degree matrices this ambiguity makes it impossible to reproduce the construction of this critical input furthermore the description of the hypergraph construction in section 2.2.1 states that the hypergraph adjacency matrix a_hyper is obtained via a_hyper = h w h^t but then introduces figure 2 and text discussing clique expansion with uniform edge weighting it is imperative that you clarify whether a_hyper is used directly as a weighted graph adjacency matrix for a standard gcn or if this is the first step in a true hypergraph convolution you must provide the exact mathematical form of the input to the hypergcn module and reconcile it with the propagation rule defined later in equations 7 and 8

3. your description of the hypergraph convolution operation in section 2.2.2 contains serious technical inaccuracies and oversimplifications regarding spectral-based methods you state that the laplacian l = i - d_v^{-1/2} h w d_e^{-1} h^t d_v^{-1/2} is used to perform low-pass filtering in the fourier domain to capture global community structure and that the weight matrix w is adjusted by structural bias in accordance with the tf-idf principle this is a vague and misleading explanation first the equation provided is a specific formulation for a hypergraph laplacian but calling it a spectral convolution without defining the actual fourier basis or explaining how the filtering is achieved beyond a single layer operation is insufficient second the analogy to tf-idf is not mathematically defined within the equation or its description you must provide a clear, step-by-step mathematical derivation of how the forward pass of your hypergcn layer works starting from the incidence matrix h, defining the propagation rule explicitly (e.g., z^{(l+1)} = σ( d_v^{-1/2} h w d_e^{-1} h^t d_v^{-1/2} z^{(l)} θ^{(l)} )) and then explaining how this specific formulation achieves the claimed effect of attenuating high-frequency components or weighting hyperedges by their uniqueness the current description is not computationally reproducible

4. the attention-guided semantic interaction module described in section 2.2.3 and equations 9-12 raises significant concerns about its intended functionality and the dimensional consistency of its operations you define an attention score ~α_i = w^t · tanh(w1 h^g_i + w2 h^h_i + b) where h^g_i and h^h_i are the final layer embeddings from the gcn and hypergcn respectively for a single herb node i the output ~α_i is then passed through sigmoid and softmax to produce a final scalar weight α_i used to fuse the representations this appears to calculate a single, global attention weight per node based on its own features, which is not a standard cross-attention mechanism between the two views you must clarify what the vectors w, w1, and w2 are and how their dimensions align to produce a scalar from the concatenated or summed input features furthermore the application of softmax over a neighborhood n_i in equation 11 for a scalar value derived from a single node is conceptually confusing you need to provide a much more detailed explanation of this mechanism, possibly with a diagram showing the tensor dimensions at each step, to demonstrate that it is not simply learning a node-specific bias but is truly aligning the two structural representations

5. the hierarchical contrastive learning section introduces both cluster-level (binary cross-entropy) and local-level (contrastive) losses but the interaction and purpose of these two levels are not clearly delineated you refer to the binary cross-entropy in equation 13 as "cluster-level" contrast, but this is simply the standard supervised loss for the prediction task and is not a contrastive loss in the traditional sense (like info-nce) this conflates the objectives and misrepresents the architecture you should rename these components to avoid confusion for instance, calling l_bce the "supervised prediction loss" and l_local the "cross-view contrastive loss" would be far more accurate furthermore, the formulation of l_local in equation 14 is for a single positive pair but it is unclear how negative samples are constructed within a batch you must specify whether negative sampling is performed across nodes within the same view, across views, or both, and how this sampling strategy impacts the learned representations

6. the experimental comparison against baseline models is severely flawed by the inclusion of two variants of the same method (htinet-knn and htinet-rf) which are not state-of-the-art deep learning models but rather simple classifiers applied to node2vec features including these weak baselines artificially inflates the perceived performance of your model, as evidenced by their extremely poor precision and f1 scores in table 5 you must replace these with more recent and competitive baselines for graph-based association prediction such as mgat (multi-channel graph attention network), dgedti (dual-graph ensemble learning), or other relevant heterogeneous graph transformers a fair comparison should pit your complex, multi-order framework against other complex, end-to-end deep learning models, not against a two-year-old method using a random forest classifier this is a critical requirement for a valid experimental setup in a top-tier journal

7. the analysis of the visualization results in section 3.3, particularly the t-sne plots in figure 7, is superficial and does not substantiate the claims made about the model's capabilities you state that the clustering patterns "underscore the model’s capacity to capture structural information" and "offer further interpretive support," but without any quantitative metric like silhouette score or a clear demonstration of how the clusters correspond to known pharmacological categories (e.g., herbs used for heat-clearing clustering together), these plots are merely qualitative and subjective you must provide a more rigorous analysis of the embedding space for example, you could select a few known functional classes of herbs and diseases and calculate the intra-class vs. inter-class distances in the embedding space to quantitatively demonstrate that the model has indeed learned meaningful semantic groupings this would transform a weak visual anecdote into a strong piece of evidence for representation quality

8. the case study presented in section 4, while interesting, suffers from a logical gap that weakens its validity as evidence for your model's predictive power you predict that niuxi is associated with skin neoplasms and then cite two separate studies one showing that niuxi polysaccharides target braf/nras in thyroid cancer and another showing a causal link between thyroid dysfunction and melanoma the logical connection you make is that because niuxi targets genes also mutated in melanoma, it may treat melanoma however, this is a post-hoc rationalization based on known literature, not a novel discovery validated by your model the model merely ranked the pair highly to prove your model's utility, you must demonstrate that this specific association (niuxi-skin neoplasms) is not explicitly present in the training data and is not trivially inferred from simple first-order similarities you should perform a deeper analysis perhaps showing that other herbs sharing similar chemical components with niuxi are also predicted to treat skin-related conditions, thereby providing a multi-evidenced, mechanistic hypothesis rooted in your model's high-order structure

9. the ablation study results presented in table 6 contain numerical inconsistencies that cast doubt on the reliability of the experimental setup for the "w/o high-order heterogeneous structure" variant on the lrsl dataset, you report an accuracy of 0.99877 and a loss of 1.09994 it is highly unusual, if not indicative of a problem, for a model to have such high accuracy while simultaneously having a loss value above 1, especially with a binary cross-entropy loss which typically ranges from 0 to ~0.7 for well-fitted models you must investigate and explain this discrepancy is the model severely overconfident and wrong on certain samples? is the loss calculated on a different scale? this requires immediate clarification and correction as it undermines the credibility of your entire ablation analysis and suggests potential issues with overfitting, numerical instability, or a bug in the evaluation code

10. the manuscript suffers from significant issues in scholarly presentation, including a garbled and nonsensical block of text on page 7 that appears to be a formatting error with repeated "1 1 1" sequences and a figure reference that is out of place such technical errors in a submitted manuscript are unacceptable and create a strong impression of carelessness furthermore, there is a clear inconsistency in the abstract where you report a +4.8% improvement on "lrsl" but in the results section, table 4, the baseline model for comparison on this dataset is not specified with an improvement value you must meticulously proofread the entire document to correct all formatting errors and ensure that every claim made in the abstract, introduction, and conclusion is directly and accurately supported by a specific table or figure in the results section the current state of the manuscript does not meet the professional standards expected for publication

Reviewer #2: The paper proposes a graph and hypergraph based contrastive learning model for predicting herb–disease associations in Traditional Chinese Medicine. Although the topic is interesting and the model integrates several modern graph learning techniques, the manuscript has several methodological, experimental, and presentation issues that significantly limit the clarity, reproducibility, and scientific rigor of the work. The following major concerns should be addressed before the manuscript can be considered for publication.

- The novelty of the proposed framework is not sufficiently justified because the model mainly combines already established components such as graph convolutional networks, hypergraph neural networks, attention based fusion, and contrastive learning without clearly demonstrating what fundamentally new methodological contribution is introduced beyond integrating existing techniques.

- The manuscript claims the construction of a new dataset named HData, yet the process of data collection, cleaning, integration, and validation is insufficiently described, particularly regarding how herb properties, chemical components, and disease associations were verified and how potential noise or inconsistent entries were handled.

- The dataset statistics appear questionable given that only a few hundred herbs and a limited number of diseases generate a very large number of confirmed herb–disease associations, which suggests an unusually dense relationship structure that may not reflect realistic biological knowledge and raises concerns about possible data leakage or artificially constructed associations.

- The procedure used to generate negative samples for the herb–disease association prediction task is not clearly explained, even though the learning objective assumes that all unobserved pairs are negative, which is a problematic assumption in biological interaction prediction where many unknown pairs may actually be undiscovered positives.

- The description of similarity calculations for herbs and diseases lacks sufficient justification and validation, particularly the use of simple binary encoding of medicinal properties and the Jaccard similarity measure, which may oversimplify complex pharmacological relationships.

- The construction of the heterogeneous adjacency matrix combining herb similarity, disease similarity, and association matrices is insufficiently explained, and the normalization procedure and its impact on graph structure and training stability are not analyzed.

- The hypergraph modeling strategy based on herb–chemical relationships is conceptually interesting but lacks discussion regarding the biological validity of clique expansion and the assumption that shared chemical components directly imply functional similarity between herbs.

-The model architecture description is fragmented and difficult to follow, as the roles of the graph encoder, hypergraph encoder, attention fusion module, and contrastive learning objectives are not clearly explained in a coherent pipeline.

-bThe attention based structural alignment module is insufficiently motivated because the manuscript does not demonstrate why this specific attention formulation is appropriate for aligning graph and hypergraph embeddings.

The paper does not provide sufficient information about hyperparameter tuning, sensitivity analysis, or the rationale behind choices such as embedding dimension, dropout rate, and learning rate, which raises concerns about reproducibility.

- The evaluation methodology relies exclusively on random five fold cross validation, which is known to produce overly optimistic results in link prediction tasks when edges are randomly split instead of using more realistic evaluation strategies such as node level or cold start splits.

- The comparative experiments with baseline methods are not sufficiently fair or transparent because there is no indication that the baseline models were properly tuned or implemented under identical experimental conditions.

The AUC values reported for some datasets are lower than competing methods while the paper still claims overall superiority, suggesting that the conclusions may selectively emphasize certain metrics while ignoring contradictory results.

Reviewer #3: This paper proposes GHF-ACL, a multi-order graph contrastive learning framework for herb–disease association prediction, which jointly models low-order functional similarities and high-order herb–chemical interactions through graph and hypergraph structures. It also introduces HData, a standardized benchmark dataset integrating herbal properties, chemical components, and disease associations, and uses an attention-guided interaction module together with hierarchical contrastive learning to align heterogeneous representations. Experimental results on one herb–disease dataset and several drug–disease benchmark datasets show that the proposed method achieves competitive or superior performance, particularly in AUPR, Recall, and F1 score, indicating its promise for imbalanced biomedical association prediction tasks.

To further strengthen the manuscript, it would be valuable to connect the proposed framework with more recent related studies and discuss how these advances could inform future methodological improvements.

1. Several formulas in the Methods section lack complete variable definitions or clear derivations, which makes the method difficult to reproduce.

For example:

(1) In the disease semantic similarity formula (Eq. 3), the variable DV(d) is used but not explicitly defined before appearing in the equation.

(2) The degree matrices (D_r) and (\tilde{D}_d) in Eq. 4 are also not clearly explained.

(3) In the hypergraph formulation (A_{hyper} = HWH^T), the derivation of this adjacency representation (from hypergraph clique expansion or spectral hypergraph theory) is not sufficiently described.

2. The local contrastive loss function (Eq. 14) appears to contain a formulation error.

(1) The denominator of the loss currently uses the same similarity term as the numerator: \sum_k \exp(sim(z_g,z_h)/\tau)

This formulation does not correctly represent contrastive learning because it does not compare the positive pair against negative samples.

(2) A correct InfoNCE-style formulation should include similarities with all other negative samples, e.g.: \sum_k \exp(sim(z_g,z_k)/\tau)

3. The task is treated as a binary classification problem for herb–disease associations. However, the manuscript does not describe how negative samples are generated, which is crucial in link prediction tasks.

Important details missing include:

whether all unknown pairs are treated as negative samples

whether random negative sampling is used

whether the dataset is balanced or imbalanced during training

Since the model uses weighted binary cross-entropy, the computation of (w^+) and (w^-) should also be clearly explained.

Without this information, the experiment cannot be reproduced.

4. Given that the proposed method is based on contrastive graph learning and hypergraph modeling, it should be compared with more recent frameworks such as: recent hypergraph neural networks (i.e., 10.1093/bib/bbaf399), graph contrastive learning models, transformer-based graph models for biomedical networks.

5. The case study presents predicted herb–disease associations such as: Niuxi → Skin Neoplasms. However, the biological evidence provided is mostly indirect literature interpretation, rather than strong validation. To better support the practical value of the model, the authors should consider: validating top predictions using external biomedical databases; performing pathway enrichment analysis; verifying predicted associations using recent pharmacological studies

Minor Revision Comments

1. English grammar and wording need improvement. Several sentences contain minor grammatical or stylistic issues. Examples include:incorrect phrasing such as “denote to”; unnecessary adverbs such as “Simultaneously”; inconsistent use of singular/plural forms

2. Figure captions and diagrams require clearer explanations. Some figures are insufficiently described. For example: Figure 1 (model architecture) includes several modules but does not clearly explain the data flow between them; The hypergraph construction process could be illustrated more clearly.

3. Hyperparameter settings lack justification. The manuscript reports the use of parameters such as: embedding dimension = 128; learning rate = 0.0001; dropout rates = 0.7 and 0.5; training epochs = 5000. However, the rationale for these choices is not discussed.

4. Dataset construction details should be clarified. The manuscript introduces a new dataset HData, which integrates herbs, diseases, and chemical components. However, some dataset preparation steps remain unclear: how duplicate herb names were resolved; how chemical component identifiers were standardized; how missing data were handled.

5. Some evaluation metrics are redundant or insufficiently discussed. The paper reports several metrics, including: Accuracy, Precision, Recall, F1 score, AUC, AUPR. However, in highly imbalanced link prediction tasks, AUPR is usually more informative than accuracy.

6. The authors may consider incorporating insights from recent studies such as 10.1016/j.csbj.2024.06.032, 10.1093/bib/bbac384, 10.1109/TCBBIO.2025.3610881, 10.1002/advs.202512453, and 10.1109/JBHI.2024.3383591, which present advanced graph-based or biomedical representation learning approaches for association prediction tasks. Integrating the methodologies or comparative analyses from these works in future research could further enhance the robustness, generalization ability, and biomedical interpretability of the proposed GHF-ACL framework.

**********

**Have the authors made all data and (if applicable) computational code underlying the findings in their manuscript fully available?**

The PLOS Data policy requires authors to make all data and code underlying the findings described in their manuscript fully available without restriction, with rare exception (please refer to the Data Availability Statement in the manuscript PDF file). The data and code should be provided as part of the manuscript or its supporting information, or deposited to a public repository. For example, in addition to summary statistics, the data points behind means, medians and variance measures should be available. If there are restrictions on publicly sharing data or code —e.g. participant privacy or use of data from a third party—those must be specified.

Reviewer #1: None

Reviewer #2: None

Reviewer #3: None

**********

PLOS authors have the option to publish the peer review history of their article (what does this mean?). If published, this will include your full peer review and any attached files.

If you choose “no”, your identity will remain anonymous but your review may still be made public.

**Do you want your identity to be public for this peer review?**  For information about this choice, including consent withdrawal, please see our Privacy Policy.

Reviewer #1: **Yes:** Mohammad Hossein Alizadeh Roknabadi

Reviewer #2: No

Reviewer #3: No

[NOTE: If reviewer comments were submitted as an attachment file, they will be attached to this email and accessible via the submission site. Please log into your account, locate the manuscript record, and check for the action link "View Attachments". If this link does not appear, there are no attachment files.]

**Figure resubmission:**

While revising your submission, we strongly recommend that you use PLOS’s NAAS tool (https://ngplosjournals.pagemajik.ai/artanalysis) to test your figure files. NAAS can convert your figure files to the TIFF file type and meet basic requirements (such as print size, resolution), or provide you with a report on issues that do not meet our requirements and that NAAS cannot fix.-->

After uploading your figures to PLOS’s NAAS tool - https://ngplosjournals.pagemajik.ai/artanalysis, NAAS will process the files provided and display the results in the "Uploaded Files" section of the page as the processing is complete. If the uploaded figures meet our requirements (or NAAS is able to fix the files to meet our requirements), the figure will be marked as "fixed" above. If NAAS is unable to fix the files, a red "failed" label will appear above. When NAAS has confirmed that the figure files meet our requirements, please download the file via the download option, and include these NAAS processed figure files when submitting your revised manuscript.

**Reproducibility:**

To enhance the reproducibility of your results, we recommend that authors of applicable studies deposit laboratory protocols in protocols.io, where a protocol can be assigned its own identifier (DOI) such that it can be cited independently in the future. Additionally, PLOS ONE offers an option to publish peer-reviewed clinical study protocols. Read more information on sharing protocols at https://plos.org/protocols?utm_medium=editorial-email&utm_source=authorletters&utm_campaign=protocols-->

# Author comment: Author response to Decision Letter 1

**DOI:** 10.1371/journal.pcbi.1014461.r002

19 Apr 2026

Attachment Submitted filename: Response to Reviewers.docx

# Peer review: Decision Letter 1

**DOI:** 10.1371/journal.pcbi.1014461.r003

**Authors:** Mark Alber, Lun Hu, Mark Alber, Lun Hu

1 Jun 2026

PCOMPBIOL-D-26-00332R1

GHF-ACL: A Novel Contrastive Learning Framework with Multi-order Graph Structures for Herb-Disease Association Prediction

PLOS Computational Biology

Dear Dr. Zhang,

Thank you for submitting your manuscript to PLOS Computational Biology. After careful consideration, we feel that it has merit but does not fully meet PLOS Computational Biology's publication criteria as it currently stands. Therefore, we invite you to submit a revised version of the manuscript that addresses the points raised during the review process.

Please submit your revised manuscript by Aug 01 2026 11:59PM. If you will need more time than this to complete your revisions, please reply to this message or contact the journal office at ploscompbiol@plos.org. When you're ready to submit your revision, log on to https://www.editorialmanager.com/pcompbiol/ and select the 'Submissions Needing Revision' folder to locate your manuscript file.

Please include the following items when submitting your revised manuscript:

* A letter that responds to each point raised by the editor and reviewer(s). You should upload this letter as a separate file labeled 'Response to Reviewers'. This file does not need to include responses to formatting updates and technical items listed in the 'Journal Requirements' section below.

* A marked-up copy of your manuscript that highlights changes made to the original version. You should upload this as a separate file labeled 'Revised Manuscript with Track Changes'.

* An unmarked version of your revised paper without tracked changes. You should upload this as a separate file labeled 'Manuscript'.

If you would like to make changes to your financial disclosure, competing interests statement, or data availability statement, please make these updates within the submission form at the time of resubmission. Guidelines for resubmitting your figure files are available below the reviewer comments at the end of this letter.

As the corresponding author, your ORCID iD is verified in the submission system and will appear in the published article. PLOS supports the use of ORCID, and we encourage all coauthors to register for an ORCID iD and use it as well. Please encourage your coauthors to verify their ORCID iD within the submission system before final acceptance, as unverified ORCID iDs will not appear in the published article. Only the individual author can complete the verification step; PLOS staff cannot verify ORCID iDs on behalf of authors.

We look forward to receiving your revised manuscript.

Kind regards,

Lun Hu

Academic Editor

PLOS Computational Biology

Mark Alber

Section Editor

PLOS Computational Biology

**Journal Requirements:**

1) Some material included in your submission may be copyrighted. According to PLOS’s copyright policy, authors who use figures or other material (e.g., graphics, clipart, maps) from another author or copyright holder must demonstrate or obtain permission to publish this material under the Creative Commons Attribution 4.0 International (CC BY 4.0) License used by PLOS journals. Please closely review the details of PLOS’s copyright requirements here: PLOS Licenses and Copyright. If you need to request permissions from a copyright holder, you may use PLOS's Copyright Content Permission form.

Please respond directly to this email and provide any known details concerning your material's license terms and permissions required for reuse, even if you have not yet obtained copyright permissions or are unsure of your material's copyright compatibility. Once you have responded and addressed all other outstanding technical requirements, you may resubmit your manuscript within Editorial Manager.

Potential Copyright Issues:

i) Figure 1 . Thank you for stating that "some graphical elements are adapted from Wikimedia Commons". Please provide a direct link to the source of the images. The link you provided is a generic one.

2) Please ensure that the figures are uploaded in the file inventory as (Fig1.tif; Fig2.tif, etc). Please make sure that the file description matches the file name in the file inventory when uploading them. Please re-upload the files accordingly. (e.g. description: Fig1, file name: Fig 1.tif).

Note: If the reviewer comments include a recommendation to cite specific previously published works, please review and evaluate these publications to determine whether they are relevant and should be cited. There is no requirement to cite these works unless the editor has indicated otherwise.

**Reviewers' comments:**

Reviewer's Responses to Questions

Reviewer #1: none

Reviewer #2: The revised manuscript has improved in several areas compared with the previous version, particularly by adding more methodological explanations, clarifying some mathematical notation, expanding the description of HData, introducing stronger baseline models, and adding additional evaluation under a node-level split. Although the paper is more complete and better organized than the previous version, but there are still several important concerns that need to be fixed before the work can be considered for publication.

The revised manuscript explains that the contribution lies in the integration of heterogeneous graph learning, hypergraph modeling, attention-based fusion, and contrastive learning for herb–disease association prediction. However, the explanation still largely frames the method as a task-specific combination of existing components rather than clearly establishing a fundamentally new methodological contribution. The authors should more explicitly distinguish their technical contribution from prior graph contrastive learning, hypergraph learning, and biomedical association prediction models, ideally by explaining which part of the architecture or learning objective is not directly inherited from existing frameworks.

The authors now describe standardization, deduplication, disease-term alignment, bioactivity screening, and manual spot-checking, but the manuscript still does not provide enough quantitative evidence about the reliability of the associations. Given the unusually dense herb–disease relationship structure, the authors should provide clearer statistics on the original number of records, removed duplicates, excluded noisy entries, retained associations, and the proportion of associations supported by multiple databases or literature sources. Without this, it remains difficult to judge whether the high density reflects real biological knowledge or possible construction bias.

The authors state that isolated nodes were removed and that HData is a high-confidence subset, but this explanation does not fully resolve the concern that filtering may artificially increase connectivity and make the prediction task easier. The authors should report how performance changes before and after removing isolated or weakly connected nodes, or at least discuss how this preprocessing choice affects task difficulty and model evaluation.

The negative sampling strategy remains confusing and somewhat inconsistent across the manuscript and response. In one part, the authors describe random negative sampling from unobserved herb–disease pairs at a 1:10 positive-to-negative ratio, while in the contrastive learning section they describe dynamic mini-batch negative sampling using other herb-node representations. These are different forms of negative sampling for different objectives, but the manuscript should separate them much more clearly. The authors should explicitly define negative samples for the supervised link-prediction loss, negative samples for the contrastive loss, and negative samples used during evaluation. They should also discuss the risk that randomly selected unobserved herb–disease pairs may include unknown positives.

The authors justify binary encoding and Jaccard similarity as appropriate for categorical TCM properties, but this remains a very simple representation of complex pharmacological relationships. The authors should either provide empirical validation showing that these similarity measures correlate with known functional or therapeutic categories, or perform a sensitivity analysis using alternative similarity definitions. Otherwise, the choice remains insufficiently justified.

The attention-based structural alignment module is now more dimensionally consistent, but the motivation remains limited. The revised formulation appears to be a node-level gating mechanism between graph and hypergraph embeddings rather than a true attention or cross-attention mechanism. This is acceptable if clearly stated, but the manuscript should avoid overstating it as semantic alignment unless additional evidence is provided. The authors should compare this gating mechanism against simpler fusion strategies such as concatenation, averaging, and fixed weighted fusion to demonstrate that the proposed module is actually necessary.

The manuscript continues to rely heavily on random five-fold cross-validation, which may overestimate performance in link prediction. The new node-level split is useful, but the authors should report the full results clearly and compare all major baselines under the same node-level or cold-start setting. It is not sufficient to state that the proposed method remains robust unless the same stricter evaluation is applied transparently to competing methods.

The discussion of AUC versus AUPR is more balanced than before, but the manuscript still needs to avoid overly broad claims of superiority. In some datasets, the proposed method has lower AUC than competing methods, while the abstract and results emphasize AUPR, recall, or F1 improvements. The authors should consistently state that the method is strongest mainly in AUPR and recall-oriented evaluation under imbalance, rather than claiming general superiority across metrics.

**********

**Have the authors made all data and (if applicable) computational code underlying the findings in their manuscript fully available?**

The PLOS Data policy requires authors to make all data and code underlying the findings described in their manuscript fully available without restriction, with rare exception (please refer to the Data Availability Statement in the manuscript PDF file). The data and code should be provided as part of the manuscript or its supporting information, or deposited to a public repository. For example, in addition to summary statistics, the data points behind means, medians and variance measures should be available. If there are restrictions on publicly sharing data or code —e.g. participant privacy or use of data from a third party—those must be specified.

Reviewer #1: None

Reviewer #2: Yes

**********

PLOS authors have the option to publish the peer review history of their article (what does this mean?). If published, this will include your full peer review and any attached files.

If you choose “no”, your identity will remain anonymous but your review may still be made public.

**Do you want your identity to be public for this peer review?**  For information about this choice, including consent withdrawal, please see our Privacy Policy.

Reviewer #1: **Yes:** Mohammad Hossein Alizadeh Roknabadi

Reviewer #2: No

[NOTE: If reviewer comments were submitted as an attachment file, they will be attached to this email and accessible via the submission site. Please log into your account, locate the manuscript record, and check for the action link "View Attachments". If this link does not appear, there are no attachment files.]

**Figure resubmission:**

-->While revising your submission, we strongly recommend that you use PLOS’s NAAS tool (https://ngplosjournals.pagemajik.ai/artanalysis) to test your figure files. NAAS can convert your figure files to the TIFF file type and meet basic requirements (such as print size, resolution), or provide you with a report on issues that do not meet our requirements and that NAAS cannot fix.-->

After uploading your figures to PLOS’s NAAS tool - https://ngplosjournals.pagemajik.ai/artanalysis, NAAS will process the files provided and display the results in the "Uploaded Files" section of the page as the processing is complete. If the uploaded figures meet our requirements (or NAAS is able to fix the files to meet our requirements), the figure will be marked as "fixed" above. If NAAS is unable to fix the files, a red "failed" label will appear above. When NAAS has confirmed that the figure files meet our requirements, please download the file via the download option, and include these NAAS processed figure files when submitting your revised manuscript.

**Reproducibility:**

To enhance the reproducibility of your results, we recommend that authors of applicable studies deposit laboratory protocols in protocols.io, where a protocol can be assigned its own identifier (DOI) such that it can be cited independently in the future. Additionally, PLOS ONE offers an option to publish peer-reviewed clinical study protocols. Read more information on sharing protocols at https://plos.org/protocols?utm_medium=editorial-email&utm_source=authorletters&utm_campaign=protocols

# Author comment: Author response to Decision Letter 2

**DOI:** 10.1371/journal.pcbi.1014461.r004

10 Jun 2026

Attachment Submitted filename: Response_to_Reviewers_auresp_2.docx

# Peer review: Decision Letter 2

**DOI:** 10.1371/journal.pcbi.1014461.r005

**Authors:** Mark Alber, Lun Hu, Mark Alber, Lun Hu, Mark Alber, Lun Hu

16 Jun 2026

Dear Miss Zhang,

We are pleased to inform you that your manuscript 'GHF-ACL: A Novel Contrastive Learning Framework with Multi-order Graph Structures for Herb-Disease Association Prediction' has been provisionally accepted for publication in PLOS Computational Biology.

Before your manuscript can be formally accepted you will need to complete some formatting changes, which you will receive in a follow up email. A member of our team will be in touch with a set of requests.

Please note that your manuscript will not be scheduled for publication until you have made the required changes, so a swift response is appreciated.

IMPORTANT: The editorial review process is now complete. PLOS will only permit corrections to spelling, formatting or significant scientific errors from this point onwards. Requests for major changes, or any which affect the scientific understanding of your work, will cause delays to the publication date of your manuscript.

Should you, your institution's press office or the journal office choose to press release your paper, you will automatically be opted out of early publication. We ask that you notify us now if you or your institution is planning to press release the article. All press must be co-ordinated with PLOS.

Thank you again for supporting Open Access publishing; we are looking forward to publishing your work in PLOS Computational Biology.

Best regards,

Lun Hu

Academic Editor

PLOS Computational Biology

Mark Alber

Section Editor

PLOS Computational Biology

***********************************************************

All reviewers were satisfied with the changes made in this revised version of the manuscript.

Reviewer's Responses to Questions

**Comments to the Authors:**

**Please note here if the review is uploaded as an attachment.**

Reviewer #2: I have carefully reviewed the revised manuscript as well as the authors' detailed response to the comments raised during the previous rounds of review. I appreciate the considerable effort the authors have invested in addressing the concerns and suggestions provided by the reviewers.

The authors have thoroughly and satisfactorily responded to all of my previous comments. The revisions made to the manuscript are appropriate, comprehensive, and well integrated into the paper. In particular, the authors have clarified the methodological aspects, strengthened the presentation of the results, improved the overall organization of the manuscript, and addressed the issues that were identified during the earlier review rounds. The quality, clarity, and scientific rigor of the manuscript have consequently improved substantially.

After examining both the revised manuscript and the point-by-point response document, I find that my major concerns have been fully resolved. The authors have demonstrated a clear commitment to improving the work and have provided convincing explanations and revisions wherever necessary.

At this stage, I do not have any further substantive comments regarding the technical content, methodology, analysis, or conclusions presented in the paper. The remaining issues are limited to minor editorial matters, such as proofreading for occasional grammatical errors, typographical mistakes, stylistic inconsistencies, and a small amount of redundancy in certain sections of the text. These minor issues can be addressed during the final editorial and production process.

I believe that the manuscript is now scientifically sound, well presented, and suitable for publication. Therefore, I recommend acceptance of the manuscript for publication, subject only to routine proofreading and editorial corrections.

**********

**Have the authors made all data and (if applicable) computational code underlying the findings in their manuscript fully available?**

The PLOS Data policy requires authors to make all data and code underlying the findings described in their manuscript fully available without restriction, with rare exception (please refer to the Data Availability Statement in the manuscript PDF file). The data and code should be provided as part of the manuscript or its supporting information, or deposited to a public repository. For example, in addition to summary statistics, the data points behind means, medians and variance measures should be available. If there are restrictions on publicly sharing data or code —e.g. participant privacy or use of data from a third party—those must be specified.

Reviewer #2: Yes

**********

PLOS authors have the option to publish the peer review history of their article (what does this mean?). If published, this will include your full peer review and any attached files.

If you choose “no”, your identity will remain anonymous but your review may still be made public.

**Do you want your identity to be public for this peer review?**  For information about this choice, including consent withdrawal, please see our Privacy Policy.

Reviewer #2: No

# Editor report: Acceptance letter

**DOI:** 10.1371/journal.pcbi.1014461.r006

**Authors:** Mark Alber, Lun Hu, Mark Alber, Lun Hu, Mark Alber, Lun Hu

PCOMPBIOL-D-26-00332R2

GHF-ACL: A Novel Contrastive Learning Framework with Multi-order Graph Structures for Herb-Disease Association Prediction

Dear Dr Zhang,

I am pleased to inform you that your manuscript has been formally accepted for publication in PLOS Computational Biology. Your manuscript is now with our production department and you will be notified of the publication date in due course.

The corresponding author will soon be receiving a typeset proof for review, to ensure errors have not been introduced during production. Please review the PDF proof of your manuscript carefully, as this is the last chance to correct any errors. Please note that major changes, or those which affect the scientific understanding of the work, will likely cause delays to the publication date of your manuscript.

Soon after your final files are uploaded, unless you have opted out, the early version of your manuscript will be published online. The date of the early version will be your article's publication date. The final article will be published to the same URL, and all versions of the paper will be accessible to readers.

For Research, Software, and Methods articles, you will receive an invoice from PLOS for your publication fee after your manuscript has reached the completed accept phase. If you receive an email requesting payment before acceptance or for any other service, this may be a phishing scheme. Learn how to identify phishing emails and protect your accounts at https://explore.plos.org/phishing.

Thank you again for supporting PLOS Computational Biology and open-access publishing. We are looking forward to publishing your work!

With kind regards,

Sharmila Kamatchi

PLOS Computational Biology | Carlyle House, Carlyle Road, Cambridge CB4 3DN | United Kingdom ploscompbiol@plos.org | Phone +44 (0) 1223-442824 | ploscompbiol.org | @PLOSCompBiol
